# Improved automated one-pot two-step radiosynthesis of (*S*)-[^18^F]FETrp, a radiotracer for PET imaging of indoleamine 2,3-dioxygenase 1 (IDO1)

**DOI:** 10.1186/s41181-024-00256-0

**Published:** 2024-04-02

**Authors:** Aurélie Maisonial-Besset, David Kryza, Klaus Kopka, Sophie Levesque, Emmanuel Moreau, Barbara Wenzel, Jean-Michel Chezal

**Affiliations:** 1 Université Clermont Auvergne, Inserm, Imagerie Moléculaire et Stratégies Théranostiques, UMR 1240, Clermont-Ferrand, F-63000 France; 2 Imthernat, LAGEPP, CNRS UMR 5007, Université de Lyon, Hospices Civils de Lyon, Lyon, F-69622 France; 3 Lumen Nuclear Medicine group, Hospices Civils de Lyon et Centre Léon Bérard, Lyon, F-69008 France; 4 Institute of Radiopharmaceutical Cancer Research, Helmholtz-Zentrum Dresden-Rossendorf, Research Site Leipzig, 04318 Leipzig, Germany; 5 School of Science, Faculty of Chemistry and Food Chemistry, Technische Universität Dresden, 01062 Dresden, Germany; 6 Department of Nuclear Medicine, Jean Perrin Comprehensive Cancer Centre, Clermont-Ferrand, F-63011 France

**Keywords:** [^18^F]FETrp, IDO1, Fluorine-18, Radiochemistry, Radiofluorination, Automation, Racemization-free radiolabelling, Circular dichroism detection

## Abstract

**Background:**

(*S*)-[^18^F]FETrp is a promising PET radiotracer for imaging IDO1 activity, one of the main enzymes involved in the tryptophan metabolism that plays a key role in several diseases including cancers. To date, the radiosynthesis of this tryptophan analogue remains highly challenging due to partial racemization occurring during the nucleophilic radiofluorination step. This work aims to develop a short, epimerization-free and efficient automated procedure of (*S*)-[^18^F]FETrp from a corresponding enantiopure tosylate precursor.

**Results:**

Enantiomerically pure (*S*)*-* and (*R*)-FETrp references as well as tosylate precursors (*S*)- and (*R*)-3 were obtained from corresponding *N*^*a*^-Boc-(L and D)-tryptophan in 2 and 4 steps, respectively. Manual optimisation of the radiolabelling conditions resulted in > 90% radiochemical conversion with more than 99% enantiomeric purity. Based on these results, the (*S*)-[^18^F]FETrp radiosynthesis was fully automated on a SynChrom R&D EVOI module to produce the radiotracer in 55.2 ± 7.5% radiochemical yield, 99.9% radiochemical purity, 99.1 ± 0.5% enantiomeric excess, and molar activity of 53.2 ± 9.3 GBq/µmol (*n* = 3).

**Conclusions:**

To avoid racemisation and complicated purification processes, currently encountered for the radiosynthesis of (*S*)-[^18^F]FETrp, we report herein significant improvements, including a versatile synthesis of enantiomerically pure tosylate precursor and reference compound and a convenient one-pot two-step automated procedure for the radiosynthesis of (*S*)-[^18^F]FETrp. This optimised and robust production method could facilitate further investigations of this relevant PET radiotracer for imaging IDO1 activity.

**Supplementary Information:**

The online version contains supplementary material available at 10.1186/s41181-024-00256-0.

## Background

Cancer immunotherapy based on immune checkpoint (IC) blockade has made impressive progress in recent years thanks to a better understanding of tumour immune escape mechanisms (Waldman et al. 2020; Guo et al. 2023). In particular, IC inhibitors (ICIs) targeting the programmed cell death protein-1/ligand-1 (PD-1/PDL-1) axis or cytotoxic T lymphocyte antigen 4 (CTLA-4), alone or in combination with further targeted- or chemo-therapies, have become the first-line treatments of numerous cancers such as advanced melanoma (Michielin et al. 2019), metastatic colorectal cancer (Morris et al. 2023), head and neck squamous cell carcinoma (Daste et al. 2024) and non-small cell lung cancer (Cortiula et al. 2022). Based on these promising therapeutic results, a broad range of ICIs targeting several immunosuppressive pathways are being developed or undergo clinical trials (Liu et al. 2022).

Indoleamine 2,3-dioxygenase 1 (IDO1) is an intracellular heme protein that catalyses along with two other enzymes, IDO2 and tryptophan 2,3-dioxygenase 2 (TDO2), the first and rate-limiting step of the tryptophan catabolism known as the kynurenine pathway (Badawy 2017). IDO1 is considered as an immune regulator that plays a pivotal role in numerous diseases such as central nervous system disorders, infections, autoimmune and inflammatory diseases, and cancers (Platten et al. 2019). Aberrant activation of IDO1 by cells located in tumours or found in the tumour microenvironment (TME) (e.g. dendritic cells, cancer-associated fibroblasts, and macrophages) is known to contribute to cancer immune escape through the combined effect of tryptophan depletion and accumulation of kynurenine and its metabolites in the tumour region (Cheong and Sun 2018). Moreover, high-level expression of IDO1 was found in a large variety of cancer types and correlated with tumour progression and poor prognosis of patients (Godin-Ethier et al. 2011). Based on these findings, IDO1 inhibitors have been designed and extensively studied alone or in combination with others IC drugs for solid tumours treatment (Le Naour et al. 2020).

However, such immunotherapies often induce adverse effects (Ibis et al. 2023), emergence of resistance (Guo et al. 2023; Pang et al. 2023) and/or low response rates (Haslam and Prasad 2019). Therefore, there is an urgent need to decipher the compensatory mechanisms that contribute to cancer resistance after IC immunotherapy (Charehjoo et al. 2023) and to identify relevant tools to improve the selection of eligible patients and/or predict the treatment response (Pilard et al. 2021). In addition and complementary to tissue and blood biomarkers under investigation (Pilard et al. 2021; Sankar et al. 2022), positron emission tomography (PET) imaging of IC and other key components of the TME are being studied intensively (Wierstra et al. 2019; van de Donk et al. 2020; Bouleau et al. 2021; Ge et al. 2022). Indeed, this non-invasive and quantitative molecular imaging methodology, allows the whole-body and repetitive assessment of the tumour and TME uptakes of specific and selective radiotracers that may provide valuable information regarding the spatiotemporal expression of IC before and during treatment, which in turn may guide the stratification of patients, predict the treatment response, thereby helping developing efficient combination therapies.

Regarding PET imaging of IDO1 activity, the main radiotracers investigated so far are ^11^C- or ^18^F-radiolabelled analogues of L- or D-tryptophan (Wierstra et al. 2019; John et al. 2020). Among them, ^18^F-labelled (*S*)-1-(2-fluoroethyl)tryptophan ((*S*)-[^18^F]FETrp), a substrate of the IDO1 enzyme (Henrottin et al. 2015), and currently under clinical investigation (NCT05556473), has demonstrated very promising preclinical results in terms of IDO1 positive-tumour cell uptake (Henrottin et al. 2016; Xin and Cai 2017; Xin et al. 2019) and pharmacokinetic profile in (patient-derived) tumour xenografts and transgenic tumour mouse models (Xin and Cai 2017; Xin et al. 2019; Xin et al. 2020; Michelhaugh et al. 2017). However, the widespread use of this radiotracer might be limited due to low radiochemical yields (RCYs) and long total radiosynthesis times of the procedures described so far (Henrottin et al. 2016; Xin and Cai 2017; Xin et al. 2019; Yue et al. 2020; Jiang et al. 2023). The latter are mainly caused by partial racemization occurring during the radiolabelling step and thus requiring lengthy and tedious chiral/reversedphase HPLC purification procedures.

Herein we describe simple and convenient syntheses of the standard compounds (*S*)- and (*R*)-FETrp and the corresponding tosylate precursors (*S*)- and (*R*)-3 with high enantiomeric excesses and the fully automated production of (*S*)-[^18^F]FETrp through a racemization-free one-pot two-step procedure.

## Methods

### General

All commercially available reagents and solvents were purchased at the following commercial suppliers: Sigma Aldrich, Alpha Aesar, ABX, Acros Organics, Fisher Scientific or Carlo Erba Reagents. *N*^*α*^-(*tert*-butoxycarbonyl)-L-tryptophan and *N*^*α*^-(*tert*-butoxycarbonyl)-D-tryptophan were purchased respectively from abcr GmbH (Karlsruhe, Germany) and Merck KGaA (Darmstadt, Germany). Whenever necessary, solvents were dried using common techniques (Armarego and Chai 2009). Unless otherwise noted, moisture-sensitive reactions were conducted under dry argon atmosphere. Temperatures indicated in the protocols correspond to the temperature of the oil bath. Analytical thin-layer chromatography (TLC) were performed on precoated silica gel 60 F_254_ plates (Merck or Macherey-Nagel) and visualised with UV light (254 nm) and/or developed with phosphomolybdic acid (8 wt%) in EtOH. Flash column chromatography was performed on silica gel 60 A normal phase, 35–70 μm (Merck or SDS). Uncorrected melting points (mp) were recorded on an electrothermal capillary digital melting point apparatus IA9100 (Bibby Scientific). Nuclear magnetic resonance (NMR) spectra (500.13 MHz for ^1^H and 125.76 MHz for ^13^C) were recorded on a Bruker Avance 500 instrument with chemical shift values (δ) expressed in parts *per* million (ppm) relative to residual solvent as standard and coupling constants (*J*) given in Hz. ^19^F NMR spectra (470.3 MHz) were recorded on a Bruker Avance 500 apparatus using trifluorotoluene as internal reference (δ -63.72 ppm). Infrared (IR) spectra were recorded in the range 4000 – 440 cm^− 1^ on a Nicolet IS10 (Fisher Scientific) with attenuated total reflectance accessory. Organic compounds were analysed by High-Resolution Mass Spectrometry (HRMS) in positive or negative mode (Waters Micromass Q-Tof micro Mass Spectrometer). Optical rotations were measured with a Jasco-P-2000 digital polarimeter at 20 °C and for concentrations (c) given in g/100 mL. Analytical chiral chromatographic measurements of the compounds were performed on a JASCO LC-4000 system incorporating a PU-4180-LPG pump, an AS-4050 autosampler (100 µL sample loop), and a UV diode array detector MD-4015 coupled with a circular dichroism (CD) chiral detector CD-4095 (monitoring at 230 nm). Data analysis was performed with the ChromNAV 2.3 C software (JASCO Deutschland GmbH, Pfungstadt, Germany). The CD spectra were recorded with the CD-4095 detector during the high-performance liquid chromatography (HPLC) run in stopped-flow mode, during which the flow through the detector cell was bypassed using a software-controlled switching valve. Each spectrum was obtained with a scanning speed of 10 nm/sec. The single enantiomers were dissolved in appropriate solvents and subjected to chiral separations, which were performed in isocratic mode at a flow rate of 1 mL/min on a CHIRALPAK IA column (250 × 4.6 mm, 5 μm, Daicel-Chiral Technologies Europe, France) for all intermediate compounds, or on a Reprosil Chiral-AA column (250 × 4.6 mm, 8 μm, Dr. Maisch, Germany) for the final compounds (*S*)- and (*R*)-FETrp. Mobile phase compositions and wavelength UV monitoring used for chiral HPLC analysis: method A: *n*-hexane/*i*-PrOH (90/10, v/v) at 280 nm, method B: *n*-hexane/*i*-PrOH/TFA (90/10/0.05, v/v/v) at 280 nm; method C: MeCN/H_2_O (82/18, v/v) at 280 nm; method D: *n*-hexane/EtOH (90/10, v/v) at 225 nm. The enantiomeric excess (*e.e.*) was determined according to a previously published procedure (Eliel 1997).

## Chemistry

### General procedure for the synthesis of (*S*)- and (*R*)-5

These compounds were obtained with slight modifications of the experimental protocol described by Kim et al. (Kim et al. 2019). To a solution of *N*-(*tert*-butoxycarbonyl)-(L or D)-tryptophan (1.50 g, 4.93 mmol) in anhydrous *N*,*N*-dimethylacetamide (DMA) (6 mL) were successively added under argon atmosphere at room temperature benzyltriethylammonium chloride (BTEAC) (1.13 g, 4.96 mmol), K_2_CO_3_ (3.41 g, 24.7 mmol) and *t*-BuBr (5.54 mL, 49.3 mmol). The reaction mixture was heated at 55 °C for 5.5 h. After cooling to room temperature, deionised H_2_O (50 mL) was added and the resulting solution was extracted with EtOAc (3 × 50 mL). The combined organic layers were successively washed with deionised H_2_O (2 × 50 mL) and brine (50 mL), dried over MgSO_4_, filtered and evaporated under reduced pressure. The residue was purified by column chromatography (SiO_2_, EtOAc/cyclohexane, 2/8, v/v) to give compound (*S*)- or (*R*)-5.

(*S*)-*tert*-Butyl 2-((*tert*-butoxycarbonyl)amino)-3-(1*H*-indol-3-yl)propanoate ((*S*)-5) was obtained according to the general method mentioned above as a white solid (1.10 g, 3.05 mmol). Yield: 62%. mp 143–144 °C (Lit.: 180–184 °C (Fujiwara et al. 2008); [α]^20^_D_ +18.8 (c 0.39, CHCl_3_); R_*f*_ = 0.26 (SiO_2_, EtOAc/cyclohexane, 2/8, v/v); IR (ATR accessory) ν 3324, 2977, 1736, 1697, 1502, 1364, 1226, 1162, 1141 cm^− 1^; ^1^H NMR (500.13 MHz, CDCl_3_) (mixture of cis-trans carbamate rotamers) δ 1.33 and 1.42 (s, 9H), 1.37 (s, 9H), 3.08 and 3.22 (dd, 1H, *J* = 14.7, 5.6 Hz), 3.08 and 3.28 (dd, 1H, *J* = 14.7, 5.7 Hz), 4.35 and 4.55 (m, 1H), 4.76 and 5.07 (d, 1H, *J* = 7.7 Hz), 7.02 (s, 1H), 7.12 (t, 1H, *J* = 7.6 Hz), 7.19 (t, 1H, *J* = 7.2 Hz), 7.35 (d, 1H, *J* = 8.1 Hz), 7.62 (d, 1H, *J* = 7.9 Hz), 8.11 (brs, 1H); ^13^C NMR (125.76 MHz, CDCl_3_) δ 28.0 (3C), 28.1, 28.4 (3C), 54.9, 79.7, 81.9, 110.5, 111.2, 119.1, 119.5, 122.1, 122.8, 128.0, 136.2, 155.4, 171.6; *e*.*e*.: >99% (chiral HPLC conditions: method A).

(*R*)-*tert*-Butyl 2-((*tert*-butoxycarbonyl)amino)-3-(1*H*-indol-3-yl)propanoate ((*R*)-5) was obtained according to the general method mentioned above as a white solid (1.00 g, 2.77 mmol). Yield: 56%. mp 140–142 °C; [α]^20^_D_ -21.4 (c 0.72, CHCl_3_); R_*f*_ = 0.26 (SiO_2_, EtOAc/cyclohexane, 2/8, v/v); IR (ATR accessory) ν 3324, 2977, 1736, 1697, 1502, 1365, 1226, 1162, 1141 cm^− 1^; ^1^H NMR (500.13 MHz, CDCl_3_) (mixture of cis-trans carbamate rotamers) δ 1.34 and 1.43 (s, 9H), 1.38 (s, 9H), 3.08 and 3.22 (dd, 1H, *J* = 14.7, 5.5 Hz), 3.08 and 3.28 (dd, 1H, *J* = 14.7, 5.5 Hz), 4.36 and 4.56 (m, 1H), 4.86 and 5.09 (d, 1H, *J* = 7.1 Hz), 7.00 (s, 1H), 7.12 (t, 1H, *J* = 7.6 Hz), 7.18 (t, 1H, *J* = 7.2 Hz), 7.34 (d, 1H, *J* = 8.1 Hz), 7.62 (d, 1H, *J* = 7.9 Hz), 8.27 (brs, 1H); ^13^C NMR (125.76 MHz, CDCl_3_) δ 28.0 (3C), 28.1, 28.4 (3C), 54.9, 79.7, 81.9, 110.4, 111.2, 119.1, 119.4, 122.0, 122.9, 128.0, 136.2, 155.4, 171.6; *e*.*e*.: >99% (chiral HPLC conditions: method A).

### General procedure for the synthesis of (*S*)- and (*R*)-4

To a solution of compound (*S*)- or (*R*)-5 (200 mg, 555 µmol) in anhydrous *N*,*N*-dimethylformamide (DMF) (2 mL) was added, under argon atmosphere at 0 °C, NaH (60 wt%, 27 mg, 675 µmol). After 5 min, 2-fluoroethyl tosylate (114 µL, 674 µmol), obtained according to the experimental protocol described by Wadsworth et al. (Wadsworth et al. 2010) (Additional file 1), was added and the reaction mixture was stirred at 0 °C for 50 min. The reaction was quenched with an aqueous saturated NH_4_Cl solution (15 mL) and extracted with EtOAc (3 × 15 mL). The combined organic layers were dried over MgSO_4_, filtered and evaporated under reduced pressure. The residue was purified by column chromatography (SiO_2_, EtOAc/cyclohexane, 2/8, v/v) to give compound (*S*)- or (*R*)-4.

(*S*)-*tert*-Butyl 2-((*tert*-butoxycarbonyl)amino)-3-(1-(2-fluoroethyl)-1*H*-indol-3-yl)propanoate ((*S*)-4) was obtained according to the general method mentioned above as a white solid (132 mg, 325 µmol). Yield: 59%. mp 111–113 °C; R_*f*_ = 0.30 (SiO_2_, EtOAc/cyclohexane, 2/8, v/v); IR (ATR accessory) ν 3500 − 3100, 2973, 2950 − 2800, 1723, 1701, 1507, 1466, 1391, 1232, 1146, 1087, 1046 cm^− 1^; ^1^H NMR (500.13 MHz, CDCl_3_) (mixture of cis-trans carbamate rotamers) δ 1.39 (s, 9H), 1.39 and 1.45 (s, 9H), 3.10 and 3.22 (dd, 1H, *J* = 14.8, 5.3 Hz), 3.10 and 3.28 (dd, 1H, *J* = 14.8, 5.5 Hz), 4.32 (dt, 2H, ^3^*J*_H−F_ = 25.8 Hz, *J* = 5.0 Hz), 4.33 and 4.56 (d, 1H, *J* = 7.5 Hz), 4.68 (dt, 2H, ^2^*J*_H−F_ = 47.0 Hz, *J* = 5.0 Hz), 4.87 and 5.09 (d, 1H, *J* = 7.5 Hz), 6.99 (s, 1H), 7.13 (t, 1H, *J* = 7.6 Hz), 7.23 (t, 1H, *J* = 7.3 Hz), 7.28 (d, 1H, *J* = 8.2 Hz), 7.63 (d, 1H, *J* = 7.9 Hz); ^13^C NMR (125.76 MHz, CDCl_3_) δ 28.0 (3C), 28.1, 28.4 (3C), 46.4 (d, 1C, ^2^*J*_C−F_ = 21.8 Hz), 54.8, 79.6, 81.9, 82.3 (d, 1C, ^1^*J*_C−F_ = 172.3 Hz), 109.0, 110.3, 119.5, 119.6, 122.0, 126.7, 128.8, 136.4, 155.4, 171.4; ^19^F NMR (470.59 MHz, CDCl_3_) δ -220.3 ppm; HRMS *m*/*z* calculated for C_22_H_32_FN_2_O_4_ [M + H]^+^: 407.2341, found: 407.2336; *e*.*e*.: 53.5% (chiral HPLC conditions: method A).

(*R*)-*tert*-Butyl 2-((*tert*-butoxycarbonyl)amino)-3-(1-(2-fluoroethyl)-1*H*-indol-3-yl)propanoate ((*R*)-4) was obtained according to the general method mentioned above as a white solid (175 mg, 431 µmol). Yield: 78%. mp 111–113 °C; R_*f*_ = 0.30 (SiO_2_, EtOAc/cyclohexane, 2/8, v/v); IR (ATR accessory) ν 3357, 2978, 2931, 1723, 1699, 1507, 1468, 1392, 1250, 1233, 1147 cm^− 1^; ^1^H NMR (500.13 MHz, CDCl_3_) (mixture of cis-trans carbamate rotamers) δ 1.39 (s, 9H), 1.39 and 1.45 (s, 9H), 3.11 and 3.22 (dd, 1H, *J* = 14.8, 5.5 Hz), 3.10 and 3.28 (dd, 1H, *J* = 14.8, 5.6 Hz), 4.35 (dt, 2H, ^3^*J*_H−F_ = 25.9 Hz, *J* = 5.0 Hz), 4.37 and 4.54 (m, 1H), 4.68 (dt, 2H, ^2^*J*_H−F_ = 47.0 Hz, *J* = 5.0 Hz), 4.80 and 5.09 (d, 1H, *J* = 8.0 Hz), 6.99 (s, 1H), 7.13 (t, 1H, *J* = 7.6 Hz), 7.23 (t, 1H, *J* = 8.1 Hz), 7.28 (d, 1H, *J* = 8.2 Hz), 7.63 (d, 1H, *J* = 7.9 Hz); ^13^C NMR (125.76 MHz, CDCl_3_) δ 28.0 (3C), 28.2, 28.4 (3C), 46.4 (d, 1C, ^2^*J*_C−F_ = 21.8 Hz), 54.8, 79.6, 81.9, 82.4 (d, 1C, ^1^*J*_C−F_ = 172.2 Hz), 109.0, 110.2, 119.4, 119.6, 122.0, 126.7, 128.8, 136.3, 155.4, 171.4; ^19^F NMR (470.59 MHz, CDCl_3_) δ -220.3 ppm; HRMS *m*/*z* calculated for C_22_H_32_FN_2_O_4_ [M + H]^+^: 407.2341, found: 407.2336; *e*.*e*.: 63.9% (chiral HPLC conditions: method A).

### General procedure for the synthesis of (*S*)- and (*R*)-6

To a solution of *N*-(*tert*-butoxycarbonyl)-(L or D)-tryptophan (1.00 g, 3.29 mmol) in anhydrous DMF (15 mL) was added under argon atmosphere at room temperature a solution of 1 M KO*t*Bu in anhydrous tetrahydrofuran (THF) (6.9 mL, 6.9 mmol). The resulting solution was cooled to 0 °C and 2-fluoroethyl tosylate (Wadsworth et al. 2010; additional file 1) (778 µL, 1.00 g, 4.6 mmol) was added. After stirring at 0 °C for 1.5 h, the reaction was quenched by successive addition of EtOAc (30 mL) and 1 M aqueous HCl solution (30 mL). The resulting mixture was decanted and the aqueous layer was extracted with EtOAc (3 × 30 mL). The combined organic layers were dried over MgSO_4_, filtered and evaporated under reduced pressure. The residue was purified by column chromatography (SiO_2_, EtOAc/cyclohexane/AcOH, 10/90/0.1 → 40/60/0.1, v/v/v), then (SiO_2_, CH_2_Cl_2_/EtOH/AcOH, 96/4/0.2, v/v/v) to give compound (*S*)- or (*R*)-6.

(*S*)-2-((*tert*-Butoxycarbonyl)amino)-3-(1-(2-fluoroethyl)-1*H*-indol-3-yl)propanoic acid ((*S*)-6) was obtained according to the general method mentioned above as a white solid (536 mg, 1.52 mmol). Yield: 47%. mp 72–74 °C; [α]^20^_D_ +4.3 (c 0.48, CHCl_3_); R_*f*_ = 0.15 (SiO_2_, EtOAc/cyclohexane/AcOH, 40/60/0.1, v/v/v); R_*f*_ = 0.27 (SiO_2_, CH_2_Cl_2_/EtOH/AcOH, 96/4/0.2, v/v/v); IR (ATR accessory) ν 3700 − 3200, 3200 − 2700, 1709, 1505, 1469, 1394, 1367, 1332, 1249, 1158, 1052, 1015 cm^− 1^; ^1^H NMR (500.13 MHz, CDCl_3_) (mixture of cis-trans carbamate rotamers) δ 1.25 and 1.43 (s, 9H), 3.08 and 3.33 (m, 2H), 4.30 (m, 2H), 4.46 and 4.70 (m, 1H), 4.64 (m, 2H), 5.06 and 6.12 (d, 1H, *J* = 7.2 Hz), 7.00 (s, 1H), 7.13 (t, 1H, *J* = 7.4 Hz), 7.23 (t, 1H, *J* = 7.1 Hz), 7.29 (d, 1H, *J* = 8.2 Hz), 7.61 (d, 1H, *J* = 7.8 Hz), 8.79 (brs, 1H); ^13^C NMR (125.76 MHz, CDCl_3_) (mixture of cis-trans carbamate rotamers) δ 27.7, 28.0 and 28.4 (3C), 46.5 (d, 1C, ^2^*J*_C−F_ = 21.7 Hz), 54.3 and 55.2 (1C), 80.3, 82.3 (d, 1C, ^1^*J*_C−F_ = 172.1 Hz), 109.3, 109.6, 119.3, 119.7, 122.2, 127.1, 128.6, 136.4, 155.7, 177.0; ^19^F NMR (470.59 MHz, CDCl_3_) (mixture of cis-trans carbamate rotamers) δ -220.2 and -220.4 ppm; HRMS *m*/*z* calculated for C_18_H_22_FN_2_O_4_ [M-H]^−^: 349.1569, found: 349.1569; *e*.*e*.: >99% (chiral HPLC conditions: method B).

(*R*)-2-((*tert*-Butoxycarbonyl)amino)-3-(1-(2-fluoroethyl)-1*H*-indol-3-yl)propanoic acid ((*R*)-6) was obtained according to the general method mentioned above as a white solid (0.87 g, 2.48 mmol). Yield: 75%. mp 72–74 °C; [α]^20^_D_ -6.0 (c 0.49, CHCl_3_); R_*f*_ = 0.15 (SiO_2_, EtOAc/cyclohexane/AcOH, 40/60/0.1, v/v/v); R_*f*_ = 0.27 (SiO_2_, CH_2_Cl_2_/EtOH/AcOH, 96/4/0.2, v/v/v); IR (ATR accessory) ν 3700 − 3200, 3200 − 2700, 1693, 1505, 1469, 1393, 1367, 1331, 1249, 1160, 1046 cm^− 1^; ^1^H NMR (500.13 MHz, CDCl_3_) (mixture of cis-trans carbamate rotamers) δ 1.25 and 1.43 (s, 9H), 3.08 and 3.33 (m, 2H), 4.31 (m, 2H), 4.46 and 4.68 (m, 1H), 4.64 (m, 2H), 5.06 and 6.13 (d, 1H, *J* = 7.4 Hz), 7.00 (s, 1H), 7.13 (t, 1H, *J* = 7.5 Hz), 7.23 (t, 1H, *J* = 7.2 Hz), 7.29 (d, 1H, *J* = 8.2 Hz), 7.61 (d, 1H, *J* = 7.9 Hz), 8.26 (brs, 1H); ^13^C NMR (125.76 MHz, CDCl_3_) (mixture of cis-trans carbamate rotamers) δ 27.7 and 28.7 (1C), 28.0 and 28.4 (3C), 46.5 (d, 1C, ^2^*J*_C−F_ = 21.5 Hz), 54.3 and 55.2 (1C), 80.3, 82.4 (d, 1C, ^1^*J*_C−F_ = 172.1 Hz), 109.3, 109.6, 119.3, 119.7, 122.2, 127.1, 128.6, 136.4, 155.7, 176.9; ^19^F NMR (470.59 MHz, CDCl_3_) (mixture of cis-trans carbamate rotamers) δ -220.2 and -220.4 ppm; HRMS *m*/*z* calculated for C_18_H_22_FN_2_O_4_ [M-H]^−^: 349.1569, found: 349.1567; *e*.*e*.: >99% (chiral HPLC conditions: method B).

### General procedure for the synthesis of hydrochloride salts of (*S*)- and (*R*)-FETrp

To a solution of (*S*)- or (*R*)-6 (200 mg, 571 µmol) in anhydrous CH_2_Cl_2_ (5 mL) was added under argon atmosphere at room temperature a solution of 2.5 M HCl in anhydrous Et_2_O (10 mL). After stirring at room temperature for 3 h, the reaction mixture was evaporated under reduced pressure. The residue was triturated with anhydrous Et_2_O (10 mL), filtered and the solid was dried overnight at 25 °C in a vacuum desiccator to give compound (*S*)- or (*R*)-FETrp.

(*S*)-2-Amino-3-(1-(2-fluoroethyl)-1*H*-indol-3-yl)propanoic acid, hydrochloride salt ((*S*)-FETrp) was obtained according to the general method mentioned above as a light grey solid (138 mg, 481 mmol). Yield: 84%. mp 222–224 °C; [α]^20^_D_ +1.8 (c 1.0, H_2_O/MeCN, 1/1, v/v); IR (ATR accessory) ν 3200 − 2700, 1758, 1590, 1500, 1470, 1346, 1192, 1182, 1112, 1089, 1037 cm^− 1^; ^1^H NMR (500.13 MHz, D_2_O) δ 3.35 (dd, 1H, *J* = 15.4, 7.1 Hz), 3.43 (dd, 1H, *J* = 15.4, 5.5 Hz), 4.29 (dd, 1H, *J* = 7.1, 5.5 Hz), 4.43 (dt, 2H, ^3^*J*_H−F_ = 28.8 Hz, *J* = 4.7 Hz), 4.73 (dt, 2H, ^2^*J*_H−F_ = 47.2 Hz, *J* = 4.7 Hz), 7.17 (t, 1H, *J* = 7.4 Hz), 7.26 (s, 1H), 7.28 (t, 1H, *J* = 7.4 Hz), 7.47 (d, 1H, *J* = 8.3 Hz), 7.63 (d, 1H, *J* = 8.0 Hz); ^13^C NMR (125.76 MHz, D_2_O) δ 25.6, 46.0 (d, 1C, ^2^*J*_C−F_ = 19.6 Hz), 53.4, 83.6 (d, 1C, ^1^*J*_C−F_ = 165.1 Hz), 106.4, 110.2, 118.6, 119.8, 122.3, 127.2, 128.7, 136.4, 172.0; ^19^F NMR (470.59 MHz, D_2_O) δ -221.0 ppm; HRMS *m*/*z* calculated for C_13_H_16_FN_2_O_2_ [M + H]^+^: 251.1190, found: 251.1187; *e*.*e*.: >99% (chiral HPLC conditions: method C).

(*R*)-2-Amino-3-(1-(2-fluoroethyl)-1*H*-indol-3-yl)propanoic acid, hydrochloride salt ((*R*)- FETrp) was obtained from (*R*)-6 (169 mg, 482 µmol) according to the general method mentioned above as a light grey solid (119 mg, 415 μmol). Yield: 86%. mp 222–224 °C; [α]^20^_D_ -2.9 (c 1.0, H_2_O/MeCN, 1/1, v/v); IR (ATR accessory) ν 3200 − 2700, 1758, 1587, 1500, 1469, 1346, 1244, 1192, 1182, 1112, 1089, 1037 cm^− 1^; ^1^H NMR (500.13 MHz, D_2_O) δ 3.35 (dd, 1H, *J* = 15.3, 7.1 Hz), 3.43 (dd, 1H, *J* = 15.3, 5.2 Hz), 4.30 (m, 1H), 4.43 (dt, 2H, ^3^*J*_H−F_ = 28.8 Hz, *J* = 4.2 Hz), 4.74 (m, 2H), 7.18 (t, 1H, *J* = 7.4 Hz), 7.26 (s, 1H), 7.29 (t, 1H, *J* = 7.3 Hz), 7.47 (d, 1H, *J* = 8.2 Hz), 7.65 (d, 1H, *J* = 7.8 Hz); ^13^C NMR (125.76 MHz, D_2_O) δ 25.6, 46.0 (d, 1C, ^2^*J*_C−F_ = 19.7 Hz), 53.4, 83.6 (d, 1C, ^1^*J*_C−F_ = 165.2 Hz), 106.4, 110.2, 118.6, 119.8, 122.3, 127.2, 128.7, 136.5, 172.0; ^19^F NMR (470.59 MHz, D_2_O) δ -221.1 ppm; HRMS *m*/*z* calculated for C_13_H_16_FN_2_O_2_ [M + H]^+^: 251.1190, found: 251.1188; *e*.*e*.: >99% (chiral HPLC conditions: method C).

### General procedure for the synthesis of (*S*)- and (*R*)-7

To a solution of *N*-(*tert*-butoxycarbonyl)-(L or D)-tryptophan (4.50 g, 14.8 mmol) in anhydrous DMF (75 mL) was added, under argon atmosphere and at room temperature, a solution of 1 M KO*t*Bu in anhydrous THF (31 mL, 31 mmol). The solution was cooled to 0 °C and benzyl 2-bromoethyl ether (3.27 mL, 20.7 mmol) was added. After stirring at 0 °C for 1 h, the reaction was quenched by successive additions of EtOAc (80 mL), an aqueous 1 M HCl solution (80 mL) and deionised H_2_O (80 mL). The mixture was decanted and the aqueous layer was extracted with EtOAc (3 × 100 mL). The combined organic layers were dried over MgSO_4_, filtered and evaporated under reduced pressure. The residue was purified by column chromatography (SiO_2_, EtOAc/cyclohexane/AcOH, 10/90/0.1 → 30/70/0.1, v/v/v) to give compound (*S*)- or (*R*)-7.

(*S*)-3-(1-(2-(Benzyloxy)ethyl)-1*H*-indol-3-yl)-2-((*tert*-butoxycarbonyl)amino)propanoic acid ((*S*)-7) was obtained according to the general method mentioned above as a white solid (4.50 g, 10.3 mmol). Yield: 69%. mp 48–50 °C; [α]^20^_D_ +3.4 (c 0.49, CHCl_3_); R_*f*_ = 0.16 (SiO_2_, EtOAc/cyclohexane/AcOH, 30/70/0.1, v/v/v); IR (ATR accessory) ν 3100 − 2800, 1709, 1496, 1468, 1453, 1393, 1367, 1333, 1248, 1159, 1104, 1057, 1027 cm^− 1^; ^1^H NMR (500.13 MHz, CDCl_3_) (mixture of cis-trans carbamate rotamers) δ 1.28 and 1.42 (s, 9H), 3.10 and 3.34 (m, 2H), 3.75 (t, 2H, *J* = 5.6 Hz), 4.26 (m, 2H), 4.44 (s, 2H), 4.44 and 4.66 (m, 1H), 5.05 and 5.80 (m, 1H), 7.02 (s, 1H), 7.12 (dt, 1H, *J* = 7.1, 0.8 Hz), 7.18 (m, 3H), 7.26 (m, 4H), 7.60 (d, 1H, *J* = 7.9 Hz), 8.06 (brs, 1H); ^13^C NMR (125.76 MHz, CDCl_3_) (mixture of cis-trans carbamate rotamers, 1 C missing) δ 27.7 and 28.9, 28.0 and 28.4 (3C), 46.3, 54.3 and 55.3 (1C), 69.0, 73.3, 80.2, 108.9, 109.5, 119.1, 119.4, 121.9, 127.5, 127.7 (2C), 127.8, 128.5 (2C), 136.5, 137.8, 155.7, 176.9; HRMS *m*/*z* calculated for C_25_H_29_N_2_O_5_ [M - H]^−^: 437.2082, found: 437.2085.

(*R*)-3-(1-(2-(Benzyloxy)ethyl)-1*H*-indol-3-yl)-2-((*tert*-butoxycarbonyl)amino)propanoic acid ((*R*)-7) was obtained from *N*-(*tert*-butoxycarbonyl)-D-tryptophan (2.00 g, 6.56 mmol) according to the general method mentioned above as a white solid (1.91 g, 4.36 mmol). Yield: 66%. mp 48–50 °C; [α]^20^_D_ -6.4 (c 0.55, CHCl_3_); R_*f*_ = 0.16 (SiO_2_, EtOAc/cyclohexane/AcOH, 30/70/0.1, v/v/v); IR (ATR accessory) ν 3100 − 2700, 1711, 1684, 1528, 1469, 1366, 1335, 1268, 1249, 1231, 1167 cm^− 1^; ^1^H NMR (500.13 MHz, CDCl_3_) (mixture of cis-trans carbamate rotamers) δ 1.25 and 1.43 (s, 9H), 3.09 and 3.34 (m, 2H), 3.75 (t, 2H, *J* = 5.5 Hz), 4.26 (m, 2H), 4.44 (s, 2H), 4.44 and 4.68 (m, 1H), 5.08 and 6.10 (d, 1H, *J* = 7.3 Hz), 7.04 (s, 1H), 7.12 (t, 1H, *J* = 7.5 Hz), 7.20 (m, 3H), 7.29 (m, 4H), 7.62 (d, 1H, *J* = 7.8 Hz), 9.54 (brs, 1H); ^13^C NMR (125.76 MHz, CDCl_3_) (mixture of cis-trans carbamate rotamers, 1 C missing) δ 27.7, 28.0 and 28.4 (3C), 46.3, 54.3 and 55.3 (1C), 69.0, 73.3, 80.2, 108.9, 109.5, 119.1, 119.4, 121.8, 127.5, 127.7 (2C), 127.8, 128.5 (2C), 136.5, 137.8, 155.7, 176.8; HRMS *m*/*z* calculated for C_25_H_29_N_2_O_5_ [M - H]^−^: 437.2082, found: 437.2083.

### General procedure for the synthesis of (*S*)- and (*R*)-8

To a solution of carboxylic acid (*S*)- or (*R*)-7 (4.50 g, 10.3 mmol) in anhydrous DMA (75 mL) were successively added, under argon atmosphere and at room temperature, BTEAC (2.34 g, 10.3 mmol) and anhydrous K_2_CO_3_ (35.46 g, 257 mmol). *t*-BuBr (55.5 mL, 492 mmol) was then slowly added dropwise over 15 min and the reaction was stirred at 55 °C for 5 h. After cooling to room temperature, the reaction mixture was poured into a mixture of ice-cold water (270 mL) and EtOAc (150 mL). The mixture was decanted and the aqueous layer was extracted with EtOAc (3 × 100 mL). The combined organic layers were washed with brine (3 × 100 mL), dried over MgSO_4_, filtered and evaporated under reduced pressure. The residue was purified by column chromatography (SiO_2_, EtOAc/cyclohexane, 1/9 → 2/8, v/v) to give compound (*S*)- or (*R*)-8.

(*S*)-*tert*-Butyl 3-(1-(2-(benzyloxy)ethyl)-1*H*-indol-3-yl)-2-((*tert*-butoxycarbonyl)amino)propanoate ((*S*)-8) was obtained according to the general method mentioned above as a colourless oil (4.61 g, 9.36 mmol). Yield: 91%. [α]^20^_D_ +14.2 (c 0.57, CHCl_3_); R_*f*_ = 0.41 (SiO_2_, EtOAc/cyclohexane, 2/8, v/v); IR (ATR accessory) ν 2976, 2931, 2849, 1708, 1495, 1468, 1454, 1392, 1365, 1249, 1145, 1059, 1027, 1014 cm^− 1^; ^1^H NMR (500.13 MHz, CDCl_3_) (mixture of cis-trans carbamate rotamers) δ 1.40 (s, 9H), 1.40 and 1.46 (s, 9H), 3.13 and 3.25 (dd, 1H, *J* = 14.8, 5.4 Hz), 3.13 and 3.31 (dd, 1H, *J* = 14.8, 5.4 Hz), 3.78 (t, 2H, *J* = 5.7 Hz), 4.29 (t, 2H, *J* = 5.7 Hz), 4.48 (s, 2H), 4.37 and 4.57 (m, 1H), 4.82 and 5.10 (d, 1H, *J* = 7.6 Hz), 7.04 (s, 1H), 7.14 (t, 1H, *J* = 7.4 Hz), 7.23 (m, 3H), 7.31 (m, 4H), 7.64 (d, 1H, *J* = 7.9 Hz); ^13^C NMR (125.76 MHz, CDCl_3_) δ 28.0, 28.1 (3C), 28.5 (3C), 46.3, 54.8, 69.1, 73.4, 79.6, 81.8, 109.3, 109.5, 119.1, 119.4, 121.7, 127.1, 127.7 (2C), 127.8, 128.5 (2C), 128.7, 136.4, 137.9, 155.4, 171.5; HRMS *m*/*z* calculated for C_29_H_39_N_2_O_5_ [M + H]^+^: 495.2854, found: 495.2845.

(*R*)-*tert*-Butyl 3-(1-(2-(benzyloxy)ethyl)-1*H*-indol-3-yl)-2-((*tert*-butoxycarbonyl)amino)propanoate ((*R*)-8) was obtained from (*R*)-7 (1.32 g, 3.01 mmol) according to the general method mentioned above as a colourless oil (1.38 g, 2.80 mmol). Yield: 93%. [α]^20^_D_ -18.7 (c 0.65, CHCl_3_); R_*f*_ = 0.41 (SiO_2_, EtOAc/cyclohexane, 2/8, v/v); IR (ATR accessory) ν 2976, 2931, 2849, 1708, 1495, 1468, 1454, 1392, 1365, 1249, 1223, 1145, 1059, 1027, 1014 cm^− 1^; ^1^H NMR (500.13 MHz, CDCl_3_) (mixture of cis-trans carbamate rotamers) δ 1.42 (s, 9H), 1.42 and 1.48 (s, 9H), 3.14 and 3.27 (dd, 1H, *J* = 14.7, 5.5 Hz), 3.14 and 3.33 (dd, 1H, *J* = 14.7, 5.5 Hz), 3.79 (t, 2H, *J* = 5.7 Hz), 4.30 (t, 2H, *J* = 5.7 Hz), 4.49 (s, 2H), 4.39 and 4.59 (m, 1H), 4.85 and 5.13 (d, 1H, *J* = 7.8 Hz), 7.05 (s, 1H), 7.16 (t, 1H, *J* = 7.4 Hz), 7.25 (m, 3H), 7.33 (m, 4H), 7.66 (d, 1H, *J* = 7.9 Hz); ^13^C NMR (125.76 MHz, CDCl_3_) δ 27.9, 28.0 (3C), 28.4 (3C), 46.3, 54.8, 69.0, 73.3, 79.5, 81.7, 109.3, 109.5, 119.1, 119.4, 121.7, 127.1, 127.6 (2C), 127.7, 128.4 (2C), 128.7, 136.4, 137.9, 155.3, 171.4; HRMS *m*/*z* calculated for C_29_H_39_N_2_O_5_ [M + H]^+^: 495.2854, found: 495.2849.

### General procedure for the synthesis of (*S*)- and (*R*)-9

To a degassed solution of benzyl derivative (*S*)- or (*R*)-8 (870 mg, 1.77 mmol) in MeOH (60 mL) was added Pd/C 10 wt% (564 mg). After stirring at room temperature for 2 h under hydrogen atmosphere, the suspension was filtered through a pad of celite^®^ 545 and the pad was washed with MeOH (2 × 10 mL). The filtrate was filtered over a 0.45 μm PTFE membrane filter and evaporated under reduced pressure to provide compound (*S*)- or (*R*)-9.

(*S*)-*tert*-Butyl 2-((*tert*-butoxycarbonyl)amino)-3-(1-(2-hydroxyethyl)-1*H*-indol-3-yl)propanoate ((*S*)-9) was obtained according to the general method mentioned above as a colourless oil (465 mg, 1.15 mmol), which was used in the next step without further purification. Yield: 65%. [α]^20^_D_ +14.9 (c 1.05, CHCl_3_); R_*f*_ = 0.47 (SiO_2_, EtOAc/cyclohexane, 5/5, v/v); IR (ATR accessory) ν 3600 − 3100, 2976, 2927, 1697, 1499, 1468, 1392, 1366, 1249, 1151, 1053, 1026 cm^− 1^; ^1^H NMR (500.13 MHz, CDCl_3_) (mixture of cis-trans carbamate rotamers) δ 1.27 and 1.31 (s, 9H), 1.42 (s, 9H), 1.88 (brs, 1H), 3.04 and 3.12 (dd, 1H, *J* = 14.6, 6.7 Hz), 3.28 (dd, 1H, *J* = 14.6, 5.0 Hz), 3.88 (q, 2H, *J* = 4.9 Hz), 4.23 (m, 2H), 4.33 and 4.49 (m, 1H), 4.72 and 5.08 (d, 1H, *J* = 8.1 Hz), 7.03 (s, 1H), 7.11 (t, 1H, *J* = 7.4 Hz), 7.19 (t, 1H, *J* = 7.5 Hz), 7.31 (d, 1H, *J* = 8.2 Hz), 7.59 (d, 1H, *J* = 7.9 Hz); ^13^C NMR (125.76 MHz, CDCl_3_) δ 28.1 (3C), 28.3 (3C), 28.4, 48.9, 55.4, 61.9, 79.6, 82.1, 109.4, 109.9, 119.3, 119.5, 121.8, 127.3, 129.1, 136.2, 155.3, 171.5; HRMS *m*/*z* calculated for C_22_H_33_N_2_O_5_ [M + H]^+^: 405.2384, found: 405.2378.

(*R*)-*tert*-Butyl 2-((*tert*-butoxycarbonyl)amino)-3-(1-(2-hydroxyethyl)-1*H*-indol-3-yl)propanoate ((*R*)-9) was obtained from (*R*)-8 (300 mg, 609 µmol) with a reaction time of 24 h according to the general method mentioned above as a colourless oil (100 mg, 247 µmol), which was used in the next step without further purification. Yield: 41%. [α]^20^_D_ -16.4 (c 1.1, CHCl_3_); R_*f*_ = 0.47 (SiO_2_, EtOAc/cyclohexane, 5/5, v/v); IR (ATR accessory) ν 3600 − 3100, 2977, 2932, 1698, 1499, 1469, 1392, 1366, 1249, 1145, 1053, 1027 cm^− 1^; ^1^H NMR (500.13 MHz, CDCl_3_) (mixture of cis-trans carbamate rotamers) δ 1.28 and 1.32 (s, 9H), 1.42 (s, 9H), 3.03 and 3.12 (dd, 1H, *J* = 14.6, 6.6 Hz), 3.27 (dd, 1H, *J* = 14.6, 5.1 Hz), 3.82 (t, 2H, *J* = 5.2 Hz), 4.18 (m, 2H), 4.33 and 4.49 (m, 1H), 4.80 and 5.11 (m, 1H), 7.01 (s, 1H), 7.10 (t, 1H, *J* = 7.4 Hz), 7.19 (t, 1H, *J* = 7.5 Hz), 7.30 (d, 1H, *J* = 8.2 Hz), 7.59 (d, 1H, *J* = 7.9 Hz); ^13^C NMR (125.76 MHz, CDCl_3_) (mixture of cis-trans carbamate rotamers) δ 28.1 (3C), 28.3 (3C), 28.7 and 29.8 (1C), 48.8, 55.3 and 55.7 (1C), 61.7, 79.6, 82.0, 109.4, 109.7, 119.3, 119.4, 121.8, 127.3, 129.0, 136.2, 155.3, 171.5; HRMS *m*/*z* calculated for C_22_H_33_N_2_O_5_ [M + H]^+^: 405.2384, found: 405.2378.

### General procedure for the synthesis of (*S*)- and (*R*)-3

To a solution of alcohol (*S*)- or (*R*)-9 (465 mg, 1.15 mmol) in anhydrous CH_2_Cl_2_ (30 mL) were successively added, under argon atmosphere and at 0 °C, triethylamine (TEA) (224 µL, 1.61 mmol), *N*,*N*-dimethylaminopyridine (DMAP) (16 mg, 131 µmol) and TsCl (377 mg, 1.96 mmol). After stirring at 0 °C for 30 min, the reaction mixture was warmed to room temperature, stirred for 20 h and evaporated under reduced pressure. The residue was purified by column chromatography (SiO_2_, EtOAc/cyclohexane 1/9→3/7, v/v) to give compound (*S*)- or (*R*)-3.

(*S*)-*tert*-Butyl 2-((*tert*-butoxycarbonyl)amino)-3-(1-(2-(tosyloxy)ethyl)-1*H*-indol-3-yl)propanoate ((*S*)-3) was obtained according to the general method mentioned above as a white solid (545 mg, 975 µmol). Yield: 85%. mp 51–53 °C; [α]^20^_D_ +19.7 (c 1.0, CHCl_3_); R_*f*_ = 0.37 (SiO_2_, EtOAc/cyclohexane, 3/7, v/v); IR (ATR accessory) ν 2977, 2928, 1709, 1495, 1468, 1364, 1250, 1191, 1171, 1151, 1000 cm^− 1^; ^1^H NMR (500.13 MHz, CDCl_3_) (mixture of cis-trans carbamate rotamers) δ 1.34 and 1.39 (s, 9H), 1.42 (s, 9H), 2.33 (s, 3H), 3.02 and 3.13 (dd, 1H, *J* = 14.8, 5.6 Hz), 3.02 and 3.22 (dd, 1H, *J* = 14.8, 5.4 Hz), 4.27 (m, 2H), 4.29 (m, 2H), 4.29 and 4.49 (m, 1H), 4.75 and 5.06 (d, 1H, *J* = 7.8 Hz), 6.83 (s, 1H), 7.10 (m, 5H), 7.46 (d, 2H, *J* = 8.0 Hz), 7.55 (d, 1H, *J* = 7.4 Hz); ^13^C NMR (125.76 MHz, CDCl_3_) δ 21.6, 27.8, 28.0 (3C), 28.4 (3C), 45.0, 54.7, 67.9, 79.6, 81.9, 108.8, 110.3, 119.4, 119.5, 122.0, 126.6, 127.6 (2C), 128.8, 129.7 (2C), 132.0, 135.9, 144.9, 155.3, 171.3; HRMS *m*/*z* calculated for C_29_H_39_N_2_O_7_S [M + H]^+^: 559.2473, found: 559.2468. *e*.*e*.: >99% (chiral HPLC conditions: method D).

(*R*)-*tert*-Butyl 2-((*tert*-butoxycarbonyl)amino)-3-(1-(2-(tosyloxy)ethyl)-1*H*-indol-3-yl)propanoate ((*R*)-3) was obtained from (*R*)-9 (167 mg, 413 µmol) according to the general method mentioned above as a white solid (126 mg, 226 µmol). Yield: 55%; mp 51–53 °C; [α]^20^_D_ -21.8 (c 1.0, CHCl_3_); R_*f*_ = 0.37 (SiO_2_, EtOAc/cyclohexane, 3/7, v/v); IR (ATR accessory) ν 2977, 2928, 1708, 1495, 1468, 1363, 1251, 1184, 1164, 1151, 999 cm^− 1^; ^1^H NMR (500.13 MHz, CDCl_3_) (mixture of cis-trans carbamate rotamers) δ 1.34 and 1.43 (s, 9H), 1.39 (s, 9H), 2.34 (s, 3H), 3.02 and 3.13 (dd, 1H, *J* = 14.8, 5.6 Hz), 3.02 and 3.22 (dd, 1H, *J* = 14.8, 5.5 Hz), 4.26 (m, 2H), 4.30 (m, 2H), 4.30 and 4.50 (d, 1H, *J* = 7.9 Hz), 4.73 and 5.05 (m, 1H), 6.83 (s, 1H), 7.10 (m, 5H), 7.47 (d, 1H, *J* = 8.1 Hz), 7.54 (d, 1H, *J* = 7.5 Hz); ^13^C NMR (125.76 MHz, CDCl_3_) δ 21.7, 27.9, 28.1 (3C), 28.4 (3C), 45.1, 54.8, 67.9, 79.7, 82.0, 108.9, 110.4, 119.4, 119.5, 122.1, 126.6, 127.7 (2C), 128.9, 129.8 (2C), 132.1, 135.9, 144.9, 155.4, 171.4; HRMS *m*/*z* calculated for C_29_H_39_N_2_O_7_S [M + H]^+^: 559.2473, found: 559.2469. *e*.*e*.: >99% (chiral HPLC conditions: method D).

## Radiochemistry

### General

No-carrier-added fluorine-18 was produced by Curium Pharma *via* the [^18^O(p, n)^18^F] nuclear reaction by irradiation of a 2.8 mL > 97%-enriched [^18^O]H_2_O target (Bruce Technology) on a PETtrace cyclotron (16 MeV proton beam, GE healthcare). The radio TLC plates (Macherey-Nagel, Precoated TLC sheets ALUGRAM^®^ Xtra SIL G/UV254) were developed with EtOAc/cyclohexane (3/7, v/v) and measured on a miniGITA Dual radio-TLC instrument (Elysia-Raytest). Analytical radio/UV-HPLC measurements were performed on a system consisting of an Agilent HP series 1100 (Hewlett Packard, Les Ulis, France) combined with a Flo-one A500 Radiomatic detector (Packard, Canberra, Australia). The analyses were carried out on a C-18 column (Agilent Zorbax Extend C18, 5 μm, 4.6 × 150 mm, equipped with a guard column) using the following solvent conditions: H_2_O containing 0.1% of trifluoroacetic acid (TFA) (solvent A) and MeCN containing 0.1% of TFA (solvent B); 0 to 15 min: gradient elution 90% → 70% A with a flow rate of 1 mL/min, λ = 290 nm. Analytical radio/chiral HPLC measurements were performed on a JASCO LC-4000 system incorporating a PU-4180-LPG pump, an AS-4050 autosampler (100 µL sample loop), an UV-4070 UV/Vis detector combined with a GabiNova Radio Flow monitor (mid energy probe 60–600 keV, Elysia-Raytest). Analyses were performed on a Reprosil Chiral-AA column (250 × 4.6 mm, 8 μm, Dr. Maisch, Germany) eluted with a mixture of H_2_O/MeCN in isocratic mode (55/45, v/v) at λ = 290 nm and with a flow rate of 1 mL/min.

Radiochemical syntheses and semi-preparative HPLC purifications were performed using a SynChrom R&D EVOI radiosynthesiser (Raytest). Sep-Pak^®^ Accell Plus QMA carbonate Plus Light cartridges (46 mg, 40 μm) and Oasis MCX plus short cartridge (225 mg, 60 μm) were purchased from Waters. Prior to use, Oasis MCX cartridges were washed with deionised H_2_O (5 mL), EtOH (5 mL) and deionised H_2_O (5 mL). Semi-preparative HPLC purifications were performed on a Symmetryprep C18 column (300 × 7.8 mm; 7 μm; Waters) eluted with a mixture of H_2_O/MeCN/TFA (90/10/0.01, v/v/v) in isocratic mode at l = 254 nm and with a flow rate of 1.5 mL/min for one min and then 2.5 mL/min. All radiolabelled compounds were analysed by radio-TLC and/or analytical radio/UV-HPLC and compared to the authentic non-radioactive materials. Radiochemical yield (RCY) refers to the measure of the proportion of a given radiotracer, isolated after purification, with respect to the starting radioactivity engaged in the radiosynthesis. The RCYs have been decay corrected. Radiochemical conversions (RCCs), used to describe the reaction efficiency, were obtained after radio-TLC analysis of an aliquot of the crude reaction and were given as the percent of activity of the integrated region of the desired product versus the activity of total strip.

### Preparation of [^18^F]fluoride solution for manual syntheses

The aqueous solution of [^18^F]fluoride in [^18^O]H_2_O was loaded on the anion exchange Sep-Pak^®^ QMA cartridge (46 mg). The trapped [^18^F]fluoride was then eluted to a glass reactor using a solution of tetra-*n*-butylammonium hydrogen carbonate (TBA-HCO_3_) in a mixture of H_2_O/MeCN (1 mL, 1/1, v/v). After elution, solvents were evaporated at 90 °C for 5 min under reduced pressure and a gentle stream of helium. A second azeotropic drying was repeated by addition of anhydrous MeCN (2.0 mL). After cooling to 25–30 °C, the dried tetra-*n*-butylammonium [^18^F]fluoride (TBA[^18^F]F) was diluted in the appropriate anhydrous solvent (MeCN or DMF) and divided into portions for the manual radiosynthesis optimisation tests described below. To note, these solutions have been used immediately after preparation to ensure high and reproducible RCC.

### Optimisation of radiolabelling: manual procedure

In a glass vial containing the precursor (*S*)-3 (1–8 mg) was added an aliquot of the TBA[^18^F]F stock solution prepared as described above (70–310 MBq). Whenever required, the solution was diluted with the appropriate solvent (i.e. anhydrous MeCN, DMF or *t*-BuOH) to reach a final volume of 500 µL. The vial was sealed and placed in a heating block preheated at 90–130 °C. After 5–10 min of heating, the vial was transferred in an ice bath and aliquots of the crude radiosynthesis mixture were analysed by radio TLC to determine RCC (Table 1). All experiments were performed in triplicate.

### Automated radiolabelling procedure

**Scheme 1:**
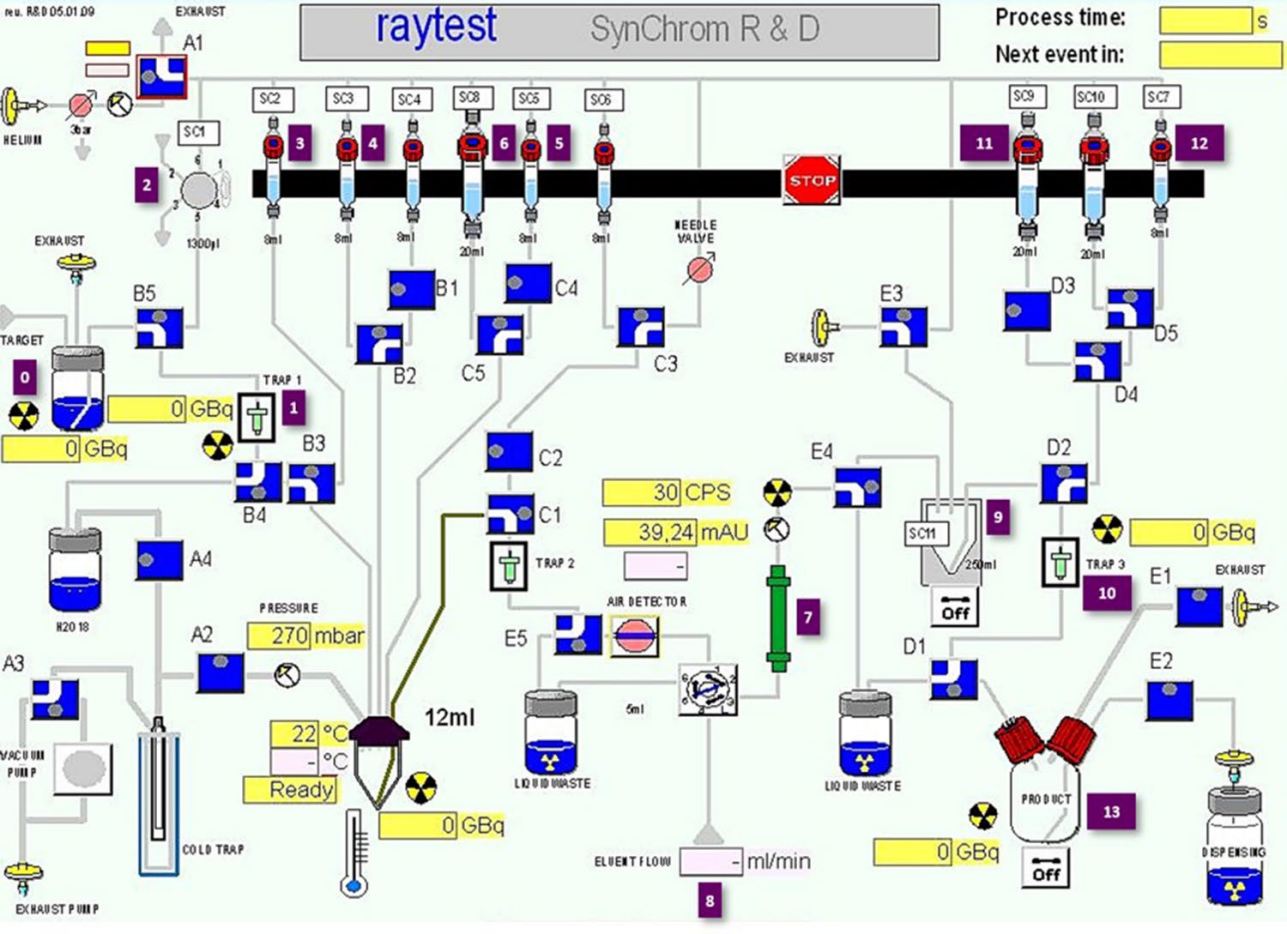
Raytest Synchrom R&D Evo I layout for the automated production of (*S*)-[^18^F]FETrp from precursor (*S*)-3. 0: aqueous solution of [^18^F]fluoride in [^18^O]H_2_O; 1: anion exchange Sep-Pak^®^ QMA cartridge (46 mg); 2: solution of TBA-HCO_3_ (4.4 mg, 14.5 μmol) in H_2_O/MeCN (1 mL, 1/1, v/v); 3: anhyd. MeCN (2 mL); 4: precursor (*S*)-3 (2 mg) in anhyd. DMF/*t*-BuOH, (1 mL, 9/1, v/v); 5: 6 M aqueous HBr solution (500 µL); 6: 1.1 M NaOAc buffer solution pH 5.5 (3.5 mL); 7: Symmetryprep C18 column (300 × 7.8 mm; 7 μm; Waters); 8: H_2_O/MeCN/TFA (90/10/0.01, v/v/v), isocratic mode, λ = 254 nm, flow rate = 1.5 mL/min for one min and then 2.5 mL/min; 9: collection vial pre-filled with deionised H_2_O (20 mL); 10: pre-conditioned Oasis MCX plus short cartridge; 11: deionised H_2_O (10 mL); 12: 5% NH_4_OH/EtOH solution (3 mL); 13: final product vial for formulation with saline (30 mL) and AcOH (135 µL)

The aqueous solution of [^18^F]fluoride in [^18^O]H_2_O, placed in the target vial (Scheme 1), was trapped on the anion exchange Sep-Pak^®^ QMA cartridge (46 mg, Trap 1). The radioactivity was eluted into the 12 mL reactor using a solution of TBA-HCO_3_ (4.4 mg, 14.5 µmol) in a mixture of H_2_O/MeCN (1 mL, SC1 loop). After 30 s of gentle helium bubbling (through needle valve), the solvents were evaporated at 90 °C under reduced pressure and helium flow (slow increase from 25 °C to 50 °C by steps of 10 °C over a 2 min period, increase to 90 °C in 1 min, heating at 90 °C for 3 min). After cooling to 25–30 °C within 2 min, anhydrous MeCN (2 mL, SC2) was added to the reactor. After 30 s of gentle helium bubbling (through needle valve), the azeotropic drying process under helium flow and reduced pressure was repeated (heating for 2 min at 35 °C, increase to 90 °C in 1 min, heating at 90 °C for 4 min). After cooling to 25–30 °C within 2–3 min, a freshly prepared solution of the precursor (*S*)-3 (2 mg) in a mixture of anhydrous DMF and *t*-BuOH, (1 mL, 9/1, v/v, SC3) was added to the dried TBA[^18^F]F. After 30 s of gentle helium bubbling (through needle valve), the reaction mixture was heated at 110 °C for 7 min (2 min of increase of temperature to reach 110 °C and further 5 min heating at 110 °C). The reactor was cooled to 30 °C before addition of a 6 M aqueous HBr solution (500 µL, SC5) under stirring. After 30 s of gentle helium bubbling (through needle valve), the reaction mixture was heated at 110 °C for 5 min. After cooling to 25–30 °C in 2–3 min, the crude reaction mixture was diluted with a 1.1 M NaOAc buffer solution pH 5.5 (3.5 mL, SC8). After 30 s of gentle helium bubbling (through needle valve), the solution containing the crude (*S*)-[^18^F]FETrp was transferred to the HPLC loop (5 mL) and purified *via* semi-preparative HPLC (conditions in general radiochemistry section). The collected fraction (*t*_R_ = 26–27 min) was diluted with deionised H_2_O (20 mL, loaded in SC11) and trapped on an Oasis MCX plus short cartridge (pre-conditioned as described in the general radiochemistry section, Trap 3). The cartridge was washed with deionised H_2_O (10 mL, SC9) and slowly eluted with 5% NH_4_OH (28–30% in H_2_O)/EtOH solution (3 mL, SC7). The final product was diluted with saline immediately after elution (30 mL, product vial) and the pH was adjusted to 5–7 using acetic acid (135 µL).

## Results

### Organic chemistry

**Fig. 1:**
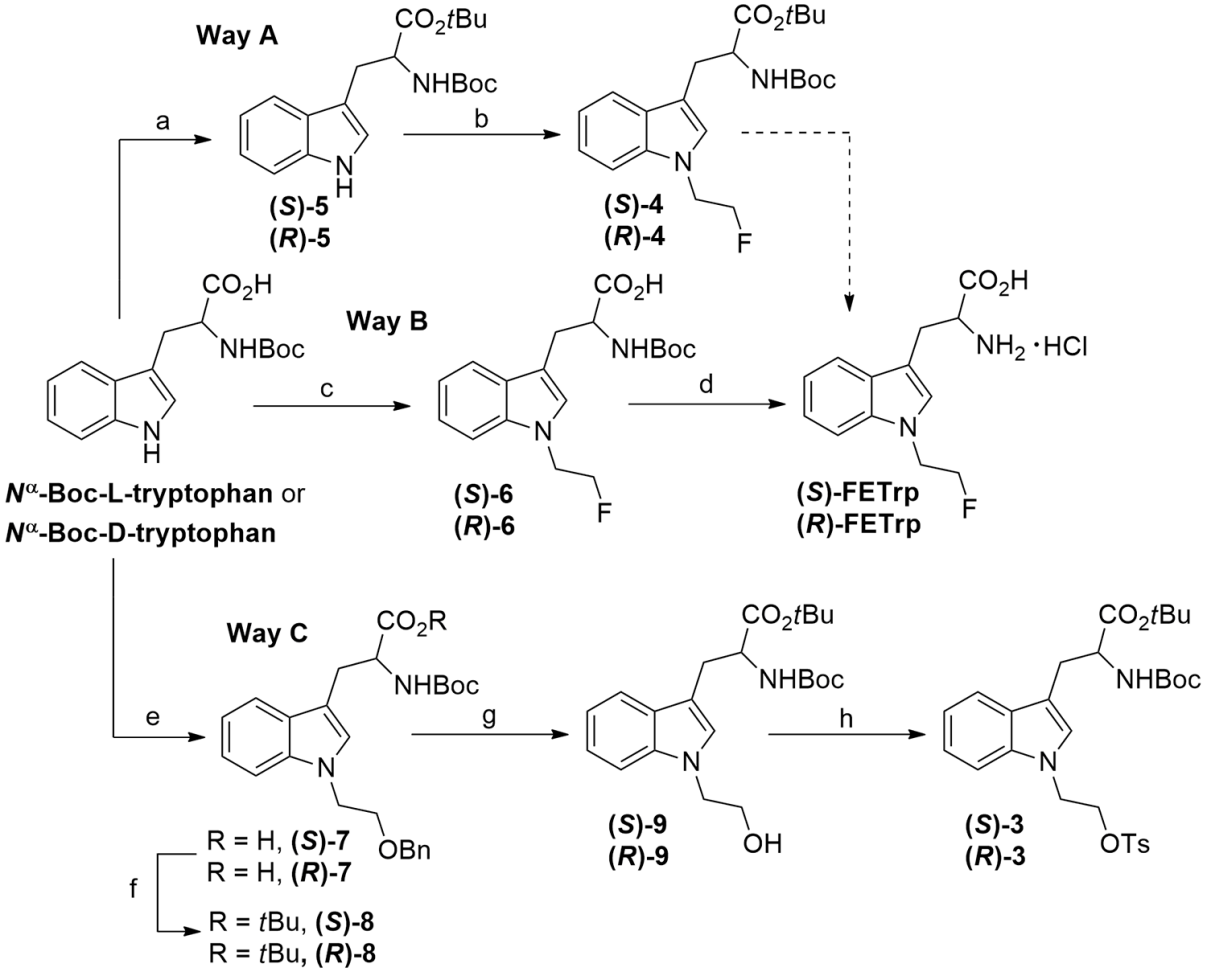
Synthesis of references (*S*)- and (*R*)-FETrp and corresponding precursors (*S*)- and (*R*)-3. (**a**) BTEAC, anhyd. K_2_CO_3_, *t*-BuBr, anhyd. DMA, 55 °C, 5.5 h; (**b**) (i) NaH 60 wt%, anhyd. DMF, 0 °C, 5 min; (ii) 2-fluoroethyl tosylate (1), 0 °C, 50 min; (**c**) (i) KO*t*Bu 1 M in anhyd. THF, anhyd. DMF, rt; (ii) 2-fluoroethyl tosylate (1), 0 °C, 1.5 h; (**d**) HCl 2.5 M in anhyd. Et_2_O, anhyd. CH_2_Cl_2_, rt, 3 h; (**e**) (i) KO*t*Bu 1 M in anhyd. THF, anhyd. DMF, rt; (ii) benzyl 2-bromoethyl ether, 0 °C, 1 h; (**f**) BTEAC, anhyd. K_2_CO_3_, *t*-BuBr, anhyd. DMA, 55 °C, 5 h; (**g**) H_2_, Pd/C 10 wt%, MeOH, rt, 2–24 h; (**h**) TsCl, TEA, DMAP, anhyd. CH_2_Cl_2_, 0 °C, 30 min then, rt, 20 h

The syntheses of enantiopure standard compounds (*S*)- and (*R*)-FETrp and precursors (*S*)- and (*R*)-FETrp are shown in Fig. 1.

Briefly, *N*^1^-alkylation of commercially available *N*^a^-Boc-L- and D-tryptophan derivatives with 2-fluoroethyltosylate (Fig. 1, Way B) or benzyl 2-bromoethyl ether (Fig. 1, Way C) in the presence of potassium *tert*-butylate gave the corresponding intermediates (*S*)- and (*R*)-6 or (*S*)- and (*R*)-7, respectively. Subsequent acidic cleavage of the *tert*-butyl ester function of (*S*)- and (*R*)-6 afforded standard references (*S*)- and (*R*)-FETrp, respectively, while the protection of the carboxylic acid function of (*S*)- and (*R*)-7 as a *tert*-butyl ester, followed by hydrogenolytic debenzylation and final tosylation gave the corresponding precursors (*S*)- and (*R*)-3.

### Radiochemistry

A broad set of different experimental conditions (i.e. amounts of base and precursor, solvent, reaction time and temperature) were manually screened with the aim to identify the key parameters that influence the RCC of (*S*)-[^18^F]4 from tosylate precursor (*S*)-3. The results are summarized in Table 1. The best RCCs (> 90% as estimated by radio-TLC of the crude reaction mixture) could be reached using low amounts of precursor (1–2 mg, 1.8–3.6 µmol), moderate reaction time (5 min) and temperature (110 °C) in MeCN or mixtures of MeCN or DMF with *t*-BuOH (Table 1, entries 11–15).

**Table 1:** Optimization of the experimental conditions for manual radiofluorination from (*S*)-3 leading to (*S*)-[^18^F]4 intermediate

Entry	precursor (mg)	TBA-HCO_3_ (µmol)	Anhyd. solvent(s) (v/v)*	Reaction temp. (°C)	Reaction time (min)	RCC** (%)
1	1	7.5	DMF	110	5	58 ± 6
2	1	7.5	DMF	110	10	54 ± 7
3	1	7.5	DMF	90	5	38 ± 3
4	1	7.5	DMF	90	10	39 ± 1
5	2	7.5	DMF	110	5	56 ± 7
6	4	7.5	DMF	110	5	40 ± 6
7	8	7.5	DMF	110	5	18 ± 4
8	1	3.75	DMF	110	5	27 ± 10
9	1	15	DMF	110	5	68 ± 10
10	1	15	DMF	130	5	58 ± 6
11	1	15	DMF/*t-*BuOH (9/1)	110	5	91 ± 2
12	1	15	DMF/*t-*BuOH (8/2)	110	5	91 ± 2
13	1	15	MeCN	110	5	97 ± 1
14	2	15	MeCN	110	5	98 ± 1
15	1	15	MeCN/*t-*BuOH (8/2)	110	5	90 ± 5

^*^ Final volume adjusted to 500 µL

^**^ RCC determined by radio-TLC of aliquots of the crude radiofluorination mixtures (*n* = 3)

Based on these findings, a fully automated radiosynthesis of (*S*)-[^18^F]FETrp on a Raytest Synchrom R&D Evo I radiosynthesizer was developed. The detailed synthesizer setup is described in Scheme 1.

The radiosynthesis of (*S*)-[^18^F]FETrp was completed in about 80–85 min in 55.2 ± 7.5% RCY (*n* = 3). Accordingly, (*S*)-[^18^F]FETrp could be obtained in excellent radiochemical purities (RCP, > 99%) and e.e. (> 99%).

## Discussion

**Fig. 2:**
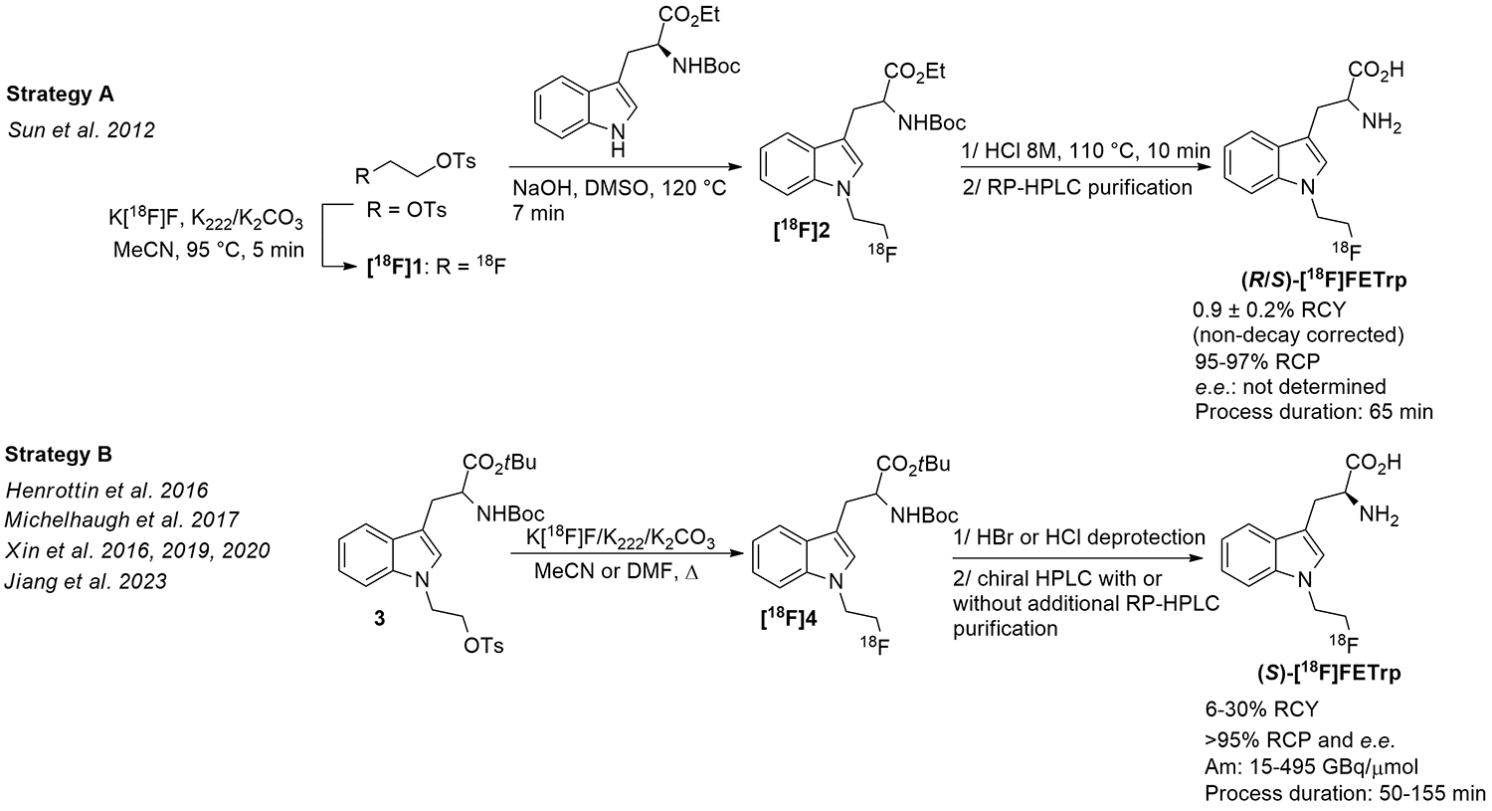
Main radiosynthetic routes available for the production of racemic (*R*/*S*)- or enantiopure (*S*)-[^18^F]FETrp

The first radiosynthesis of [^18^F]FETrp was reported by Sun et al. (Sun et al. 2012) according to a two-pot three-step procedure, including: (i) classical radiosynthesis of 2-[^18^F]fluoroethyl tosylate ([^18^F]1); (ii) *N*-alkylation of the precursor *N*^*α*^-Boc-L-tryptophan ethyl ester with [^18^F]1; and (iii) cleavage of the amino acid protecting groups of intermediate [^18^F]2 before final RP-HPLC purification (Fig. 2, strategy A).

Accordingly, [^18^F]FETrp was obtained through a 65 min radiosynthesis, in low RCY (0.9 ± 0.2%, non-decay corrected) and without final control of the radiotracer stereochemistry. Subsequently and taking into account that (*S*)-[^18^F]FETrp has demonstrated both in vitro and in vivo greater specificity for IDO1 than its enantiomer (*R*)-[^18^F]FETrp (Henrottin et al. 2016; Xin and Cai 2017), efforts have been done to develop a more efficient and asymmetric radiolabelling procedure for the production of (*S*)-[^18^F]FETrp. As shown in Fig. 2 (strategy B), all the radiosyntheses published so far have in common the nucleophilic substitution of the tosylate group of precursor 3 with the K[^18^F]F/K_222_/K_2_CO_3_ complex, followed by removal of the *tert*-butoxycarbonyl (Boc) and *tert*-butyl ester groups of intermediate [^18^F]4 in acidic conditions, giving access to the (*S*)-[^18^F]FETrp enantiomer after chiral semi-preparative HPLC purification. To fulfill good manufacturing practices requirements, additional semi-preparative RP-HPLC purification was required to remove chemical impurities co-eluting with (*S*)-[^18^F]FETrp during the chiral HPLC separation step and thus increasing procedure times (Yue et al. 2020; Jiang et al. 2023). Overall, these radiosynthesis strategies resulted in the production of (*S*)-[^18^F]FETrp with RCYs of 6–30%, excellent RCP and *e*.*e*. (both > 95%), and moderate to high molar activities (A_m_) (15 to 495 GBq/µmol). The process durations range between 50 and 155 min. The main drawback of these strategies remains the partial racemization that occurred during the nucleophilic [^18^F]fluoride substitution reaction, leading to [^18^F]4 in a 2:1 ratio of the (*S*):(*R*) enantiomers even when the optically pure tosylate precursor (*S*)-3 was used. As postulated by Xin et al. (Xin and Cai 2017), we assumed that this phenomenon was most likely due to the basic conditions of the radiofluorination step using the K[^18^F]F/K_222_/K_2_CO_3_ complex that are sufficient enough to partially deprotonate the α-C-H of the amino ester group. To confirm this hypothesis and find optimal radiolabelling conditions for a racemization-free radiofluorination of (*S*)-[^18^F]FETrp, it was first necessary to synthesise both enantiomers of FETrp and precursor 3 with high optical purities.

### Organic chemistry

Apart of chemical supplier sources, there is only one published asymmetric route to the tosylate precursor (*S*)-3 with assumed 78% *e*.*e*. (Xin and Cai 2017), while the synthesis of the pure enantiomer (*S*)-FETrp was reported in a single literature reference on a milligram scale using enzymatic kinetic resolution (Henrottin et al. 2016) (see Fig. S1, additional file 1).

**Fig. 3:**
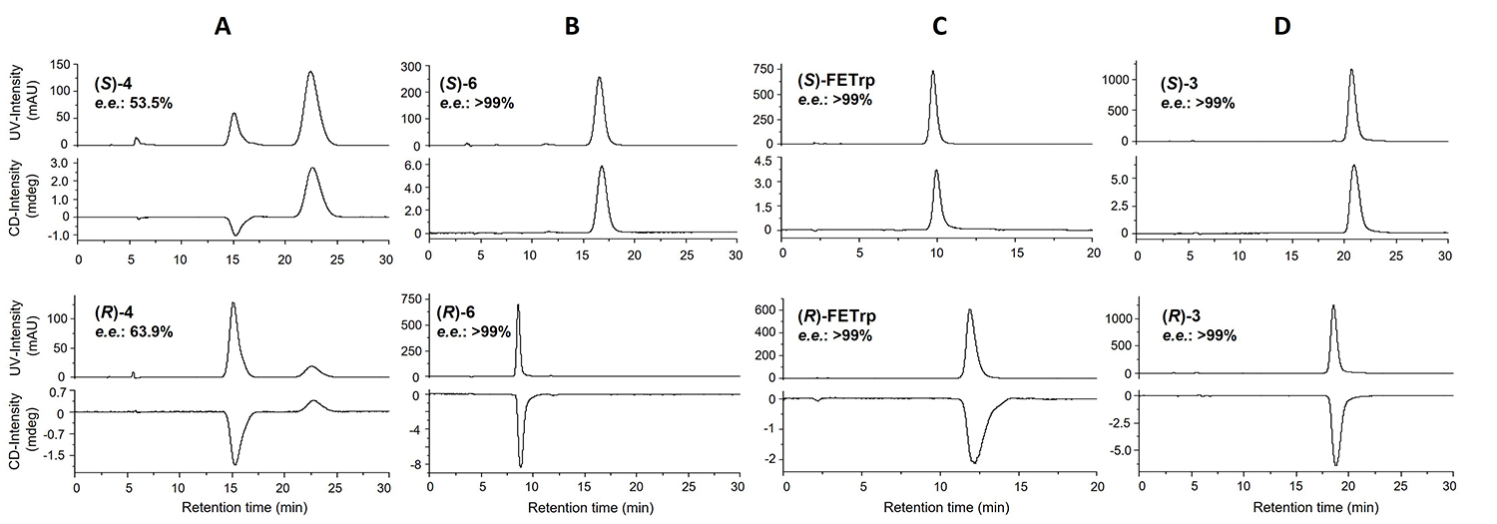
Representative chromatograms of the chiral UV-CD-HPLC analyses of compounds 4, 6, FETrp and 3. Conditions: CD detection at 230 nm, (**A**) CHIRALPAK IA, *n*-hexane/*i*-PrOH (90/10, v/v), UV detection at 280 nm; (**B**) CHIRALPAK IA, *n*-hexane/*i*-PrOH/TFA (80/20/0.05, v/v/v), UV detection at 280 nm; (**C**) Reprosil Chiral-AA, MeCN/H_2_O (82/18, v/v), UV detection at 280 nm; (**D**) CHIRALPAK IA, *n*-hexane/EtOH (90/10), UV detection at 225 nm

Based on the previously published procedures (Henrottin et al. 2016; Xin and Cai 2017), different conditions were screened to introduce the fluoroethyl group by *N*^1^-alkylation of the *N*^α^-Boc-L- or D-tryptophan *t-*Bu ester (*S*)- or (*R*)-5, obtained from corresponding *N*^α^-Boc-L- or D-tryptophan (Fig. 1, Way A) with > 99% *e*.*e*. (see Fig. S2, additional file 1). The reaction was conducted at 0 °C with reduced amount of NaH (1.2 eq) and shorter reaction time (50 min) than previously described. Unfortunately, these milder conditions led to partial racemization of the esters (*S*)-4 or (*R*)-4 revealed by chiral UV-CD-HPLC (53.5 or 63.9% *e*.*e*., respectively) (Fig. 3A).

The same trend was observed when lithium bis(trimethylsilyl)amide was employed as a base in THF at -78 °C according to a previously reported procedure for the synthesis of *N*^1^-prenylated *N*^α^-Boc-L-tryptophan methyl ester (Yu et al. 2020) (data not shown). To work around this problem, a recent method for the *N*^1^-alkylation of tryptophan derivatives was investigated (Junk et al. 2021). This interesting strategy requires the use of unprotected carboxylic acid tryptophan derivatives in the presence of 2.1 equivalents of KO*t*Bu, a weaker base compared to NaH. The first base equivalent leads to the formation of the corresponding carboxylate, which in turn decreases the acidity of the α-C-H, thus avoiding the enolate formation and epimerisation of the chiral centre. The esterification side reaction could also be reduced when the reaction was conducted in DMF at 0 °C. Accordingly, both enantiomers of the *N*^1^-fluoroethyl derivative 6 were obtained from *N*^α^-Boc-tryptophan by *N*^1^-alkylation with 2-fluoroethyl tosylate in good chemical yields (47–75%) (Fig. 1, Way B) and excellent *e*.*e*. (> 99%) (Fig. 3B). Removal of the Boc protecting group of (*S*)- and (*R*)-6 was then achieved by using HCl 2.5 M in anhydrous Et_2_O to yield standard references (*S*)- and (*R*)-FETrp, as hydrochloride salts, with high optical purities (> 99% *e*.*e*.) (Fig. 3C).

Based on these results, we then applied this efficient procedure to obtain the precursor (*S*)-3 and its enantiomer (*R*)-3 (Fig. 1, Way C). Briefly, L and D isomers of *N*^α^-Boc-tryptophan were alkylated with benzyl 2-bromoethyl ether to yield the *N*^1^-benzyloxyethyl derivatives (*S*)- and (*R*)-7. Then, *tert*-butyl esterification according to the literature procedure of Chevallet et al. (Chevallet et al. 1993), followed by Pd/C-catalysed hydrogenolysis of the benzyl protecting group of (*S*)- and (*R*)-8 gave the alcohols (*S*)- and (*R*)-9, which were converted into their corresponding tosylates (*S*)- and (*R*)-3, respectively. Following this procedure, high enantiomeric purities (up to 99% *e*.*e*. determined by chiral-HPLC analyses) were attained for both enantiomers of precursor 3 (Fig. 3D).

### Radiochemistry

With optically pure standard references and precursors in hands, aliphatic nucleophilic substitution of precursor (*S*)-3 using [^18^F]fluoride was then investigated. To achieve less basic radiofluorination conditions, we turned our attention to TBA[^18^F]F as an alternative to the K[^18^F]F/K_222_/K_2_CO_3_ complex (Haveman et al. 2023). First of all, we tested the elution efficiency of [^18^F]fluoride trapped on the QMA cartridge with increasing concentrations of TBA-HCO_3_ in a mixture of H_2_O/MeCN (1 mL, 1/1, v/v). We determined that a concentration ≥ 7.5 mM of TBA-HCO_3_ (i.e. amount of TBA-HCO_3_ ≥ 7.5 µmol) was required to recover at least 85% of [^18^F]fluoride in the reactor. We then manually explored a set of experimental conditions (molar amounts of base and precursor, solvent, reaction time and temperature) for the radiofluorination of the tosylate precursor (*S*)-3 (Table 1).In order to minimise the base content, the initial experiments were carried out with a fixed amount of 7.5 µmol of TBA-HCO_3_ (Table 1, entries 1–7). When using 1 mg (1.8 µmol) of precursor in anhydrous DMF at 110 °C, 58 ± 6% of the protected [^18^F]FETrp intermediate [^18^F]4 was obtained after 5 min. Lowering the temperature from 110 to 90 °C decreased the RCC (Table 1, entries 3 and 4), while prolonged reaction time had no significant impact on the radiolabelling efficiency (Table 1, entries 2 and 4). Increasing the amount of precursor from 1 to 8 mg led to a noticeable decrease of the RCC by 40%. The radiolabelling efficiency was also dependent on the amount of base used. Indeed, lowering the amount of TBA-HCO_3_ to 3.75 µmol reduced significantly the RCC, while doubling the amount of base to 15 µmol led to a slight increase of the RCC (Table 1, entries 8 and 9). At this stage and in order to verify that such large amounts of base did not lead to partial racemization, crude [^18^F]4, obtained from conditions of entry 9, was fully deprotected using 6 M aqueous HBr solution at 110 °C for 5 min according to the procedure of Henrottin et al. (Henrottin et al. 2016). After purification by semipreparative HPLC, high RCP as well as *e*.*e*. of (*S*)-[^18^F]FETrp (> 98% determined by radio-(chiral)-HPLC analyses) were obtained thus validating the choice of TBA-HCO_3_ as base for this radiosynthesis. Raising the reaction temperature to 130 °C did not give any further benefits (Table 1, entry 10). Next the effect of the solvent was investigated, while keeping constant the amount of base (i.e. 15 µmol). Running the reaction in anhydrous DMF in the presence of 10% and 20% of *t*-BuOH, known sometimes to greatly enhance the nucleophilic radiofluorination rates (Kim et al. 2006), significantly improved the RCC (91% for both) (Table 1, entries 11 and 12). When MeCN was used, the protected intermediate [^18^F]4, from 1 to 2 mg (1.8 or 3.6 µmol) of precursor (*S*)-3, was produced in near-quantitative RCC (Table 1, entries 13 and 14). In contrast to DMF, the addition of *t*-BuOH as co-solvent with MeCN was found to have a negative impact on the radiolabelling efficiency (Table 1, entry 15). Interestingly, in all conditions tested, apart from unreacted [^18^F]fluoride, only the protected derivative [^18^F]4 was detected using radio-HPLC and radio-TLC analyses of the crude reaction mixture.

**Fig. 4:**
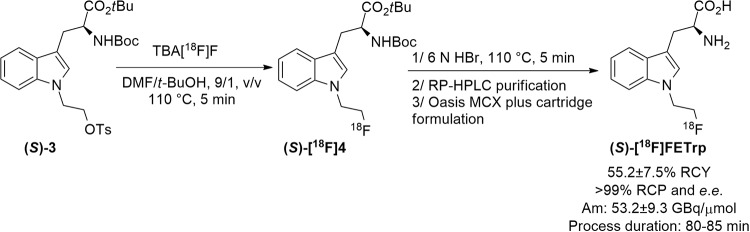
In-house developed enantiospecific one-pot two-step radiosynthesis of (*S*)-[^18^F]FETrp

**Fig. 5:**
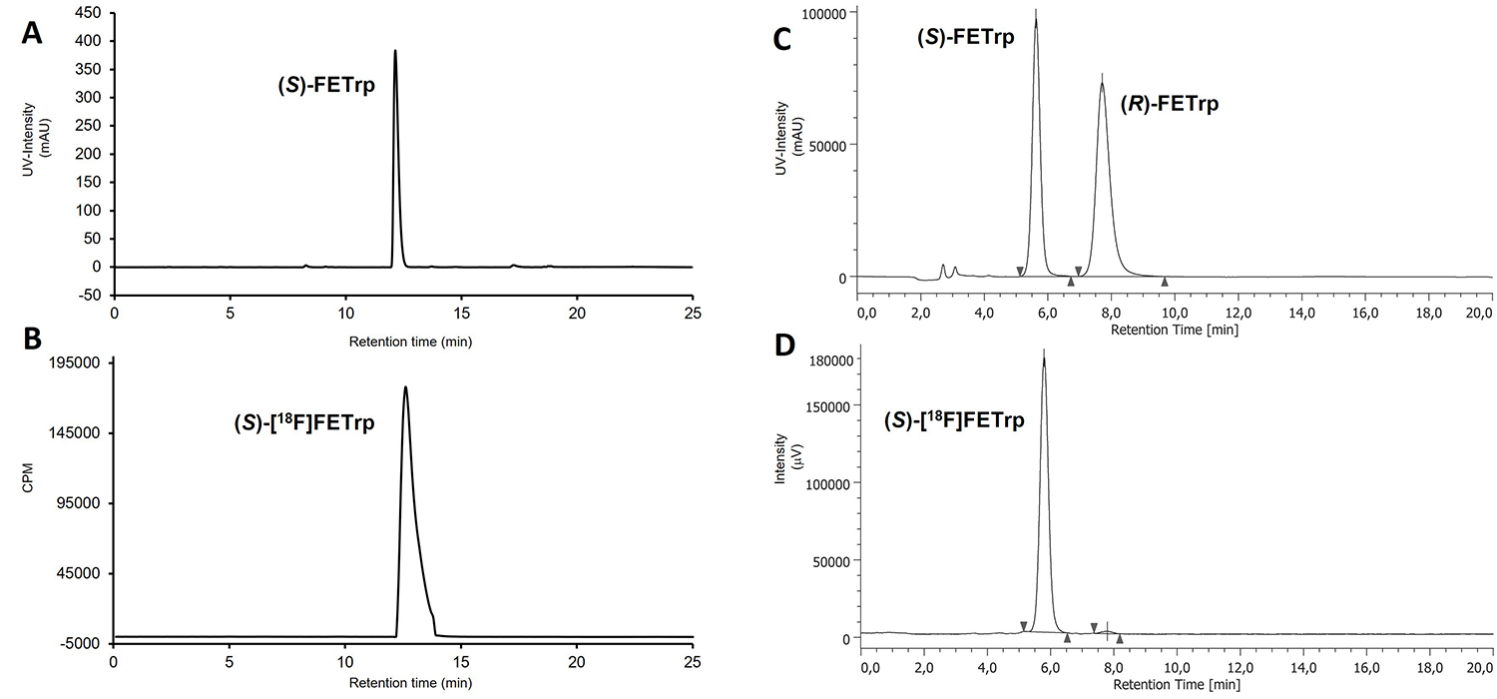
Representative HPLC chromatograms of formulated (*S*)-[^18^F]FETrp. Analytical HPLC chromatograms of (*S*)-[^18^F]FETrp (**B**) (radioactivity) co-injected with (*S*)-FETrp (**A**) (UV, λ at 290 nm) on Agilent Zorbax Extend C18 column. Chiral HPLC chromatograms of (*S*)-[^18^F]FETrp (**D**) (radioactivity) co-injected with (*S/R*)-FETrp (**C**) (UV, λ at 290 nm) on Reprosil Chiral-AA column. The radio-HPLC detectors were connected in series after the UV detectors accounting for the slight differences in retention times (∼0.3 min) observed between UV and radioactive signals

Based on these findings, the optimised reaction conditions obtained (Table 1, entry 13, RCC: 98 ± 1%) were then applied on a Raytest Synchrom R&D Evo I radiosynthesizer to develop a novel automated radiosynthesis of (*S*)-[^18^F]FETrp (Scheme 1). Briefly, after trapping [^18^F]fluoride (2.0-3.8 GBq) on the anion exchange cartridge, elution of the activity to the reaction vessel using a solution of TBA-HCO_3_ (15 µmol, elution efficiency: 97.8 ± 0.9%) and two successive azeotropic drying steps under helium stream and reduced pressure, the resulting anhydrous TBA[^18^F]F was allowed to react with the tosylate precursor (*S*)-3 in anhydrous MeCN at 110 °C for 5 min. The RCC was controlled by radio-TLC analysis of the crude reaction mixture to ensure that the automated process gave similar results than the manual radiosynthesis. Surprisingly, the RCC dropped to 64% and after the acidic deprotection step, the *e*.*e*. of the final radiotracer was also significantly impaired (87% *e*.*e*.). Having noticed that the pressure in the closed reaction vessel increased significantly during the heating at 110 °C, we hypothesized that the geometry and the total volume of the reaction vessel (12 mL in the automate vs. 7 mL for the manual process) induced the partial vaporization of the solvent (i.e. MeCN), increasing the concentrations of the precursor (*S*)-3 and the TBA-HCO_3_ base in the reaction media thus contributing to the significant decrease in RCC and partial racemization of (*S*)-[^18^F]FETrp. In consequence, lower amounts of TBA-HCO_3_ (7.5, 11.2, and 14.5 µmol) and/or larger volumes of MeCN (750, and 1000 µL) were then investigated. Under these conditions, only RCC of 15-47% could be achieved while the *e*.*e*. was still lower than 93%. Being unsuccessful in reproducing the manual conditions optimised with MeCN, we decided to explore the use of the DMF/*t*-BuOH mixture (9/1, v/v, 500 µL) depicted in Table 1 (entry 11, RCC: 91 ± 2%). Under these conditions, RCC of 50% could be achieved with significantly lower overpressure in the reaction vessel (1340 mbar vs. >2000 mbar with MeCN). However, the *e*.*e*. of the final (*S*)-[^18^F]FETrp obtained after acidic cleavage of the protective groups was still sub-optimal (87%). To address this challenge, the TBA-HCO_3_ concentration in the reaction media was reduced by half by adjusting the volume of the reaction mixture to 1 mL while maintaining the concentration of precursor (i.e. 2 mg/mL). Applying these conditions, higher RCC of 89.8 ± 5.8% (*n* = 4) could be achieved. Then, the deprotection of the intermediate [^18^F]4 using a 6 M aqueous HBr solution was performed and followed by purification of the crude product by semi-preparative RP-HPLC (see Fig. S3, additional file 1). The collected fraction was diluted with water and the final formulation of the radiotracer was performed by solid-phase extraction using an Oasis MCX plus cartridge. Elution under basic conditions, as described by Jiang et al. (Jiang et al. 2023) successfully afforded (*S*)-[^18^F]FETrp in 55.2 ± 7.5% RCY, 99.9% RCP, 99.1 ± 0.5% *e*.*e*. and A_m_ of 53.2 ± 9.3 GBq/µmol (*n* = 3) with a total process duration of 80–85 min (Figs. 4 and 5).

## Conclusion

In summary, we have completed the synthesis of enantiomerically pure (*S*)- and (*R*)-FETrp and tosylate precursor (*S*)-3. Manual radiolabelling screening was performed to find the optimal reaction conditions for the radiosynthesis of (*S*)-[^18^F]FETrp. The use of low amounts of precursor, short reaction times and TBA-HCO_3_ as the base was found to be important to achieve high RCC and *e*.*e*.

The transfer of the optimal manual labelling parameters to an automated radiosynthesizer (SynChrom R&D EVOI) required a few additional modifications regarding the total reaction volume in order to achieve high RCC in the crude reaction mixture (89.8 ± 5.8%, *n* = 4) while maintaining a very high e.e. (> 99%). A fully automated one-pot two-step radiosynthesis of (*S*)-[^18^F]FETrp could be finally achieved after acidic deprotection, semi-preparative RP-HPLC purification and final formulation *via* solid-phase extraction in 80–85 min. This simple and convenient procedure represents a significant improvement compared to the previously published protocols in terms of the purification process, the total radiosynthesis time and overall RCY (Fig. 4). We assume that this process could be easily implemented to other existing radiosynthesis modules and will broaden the use of (*S*)-[^18^F]FETrp for PET imaging of IDO1 activity with potential benefits in a wide range of physiological and pathophysiological applications.

### Electronic supplementary material

Below is the link to the electronic supplementary material.

Supplementary Material 1

## Data Availability

The datasets used and/or analysed during the current study are available from the corresponding author on reasonable request.

## Abbreviations

A_m_: Molar activity
Anhyd.: Anhydrous
Boc: *tert*-Butoxycarbonyl
BTEAC: Benzyltriethylammonium chloride
CD: Circular dichroism
DMF: *N*,*N*-dimethylformamide
DMA: *N*,*N*-dimethylacetamide
DMAP: *N*,*N*-dimethylaminopyridine
*e*.*e*.: Enantiomeric excess
FETrp: 1-(2-Fluoroethyl)tryptophan
HPLC: High-performance liquid chromatography
HRMS: High resolution mass spectrometry
IC: Immune checkpoint
ICI: Immune checkpoint inhibitor
IDO: Indolamine 2,3-dioxygenase
IR: Infrared
NMR: Nuclear magnetic resonance
PET: Positron emission tomography
PTFE: Polytetrafluoroethylene
ppm: Parts *per* million
QMA: Quaternary methyl ammonium
RCC: Radiochemical conversion
RCY: Radiochemical yield
RCP: Radiochemical purity
RP: Reverse phase
rt: Room temperature
TBA[^18^F]F: tetra-*n*-butylammonium [^18^F]fluoride
TEA: Triethylamine
TFA: Trifluoroacetic acid
THF: Tetrahydrofuran
TLC: Thin-layer chromatography
TME: Tumour microenvironment
*t*_R_: retention time
UV: Ultraviolet
