# Supplementary material for: Improved automated one-pot two-step radiosynthesis of (S)-[18F]FETrp, a radiotracer for PET imaging of indoleamine 2,3-dioxygenase 1 (IDO1)
Source: EJNMMI Radiopharm Chem. 2024 Apr 2;9:28. doi: 10.1186/s41181-024-00256-0 (PMC10987429; doi:10.1186/s41181-024-00256-0)
Supplement: Supplementary file 1 — Supplementary Material 1 [file 41181_2024_256_MOESM1_ESM.pdf]

Supporting Information

Improved automated one-pot two-step radiosynthesis of (*S*)-[^{18}F]FETrp, a radiotracer for PET imaging of indoleamine 2,3-dioxygenase 1 (IDO1)

Aurélie Maisonial-Besset[a], David Kryza[b,c], Klaus Kopka[d,e], Sophie Levesque[a,f], Emmanuel Moreau[a], Barbara Wenzel[d], Jean-Michel Chezal[a,*]

[a]Université Clermont Auvergne, Inserm, Imagerie Moléculaire et Stratégies Théranostiques, UMR 1240, F-63000 Clermont-Ferrand, France.

[b]Imthernat, LAGEPP, CNRS UMR 5007, Université de Lyon, Hospices Civils de Lyon, F-69622, Lyon, France.

[c]Lumen Nuclear Medicine group, Hospices Civils de Lyon et Centre Léon Bérard, F-69008 Lyon, France.

[d]Helmholtz-Zentrum Dresden-Rossendorf, Institute of Radiopharmaceutical Cancer Research, Research Site Leipzig, 04318 Leipzig, Germany.

[e]Technische Universität Dresden, School of Science, Faculty of Chemistry and Food Chemistry, 01062 Dresden, Germany.

[e]Department of Nuclear Medicine, Jean Perrin Comprehensive Cancer Centre, F-63011 Clermont-Ferrand, France.

*Corresponding author.
E-mail address: j-michel.chezal@uca.fr

Table of contents

1. Synthesis of 2-fluoroethyl tosylate (1) S3

2. Supplementary figures

Fig S1 Synthesis of **(*S*)-[^{18}F]FETrp** and precursor **(*S*)-3** according to previously published protocols S4

Fig S2 Representative chiral UV/circular dichroism-HPLC chromatograms of compound **5** S4

Fig S3 UV and radioactivity chromatograms of the semi-preparative purification of **(*S*)-[^{18}F]FETrp**. S5

3. ^{1}H, ^{19}F and ^{13}C NMR spectra of all synthesised compounds S6

4. References S26

1. Synthesis of 2-fluoroethyl tosylate (1)

This compound was obtained according to the experimental protocol described by Wadsworth et al. (Wadsworth et al. 2010). to a solution of 2-fluoroethanol (2.72 mL, 46.9 mmol) in anhydrous pyridine (45 mL) cooled to 0 °C was added portionwise over a period of 30 min and under argon atmosphere TsCl (19.64 g, 103 mmol) keeping the temperature < 5°C. After stirring the reaction mixture for 3 h at 0 °C, ice and then deionised H_2O (90 mL) were successively added and the resulting solution was extracted with EtOAc (3 x 90 mL). The combined organic layers were washed with deionised H_2O (90 mL) and then 1 M aqueous HCl solution (4 x 90 mL) until the pH of the aqueous layer became acidic. The organic layer was washed successively with 1 M aqueous Na_2CO_3 solution (3 x 90 mL) and brine (90 mL), dried over $MgSO_4$, filtered and evaporated under reduced pressure to give compound **1** as a colourless oil (10.17 g, 46.6 mmol). Yield: 99%. R_f = 0.59 (SiO_2, EtOAc/cyclohexane 3/7, v/v); IR (ATR accessory) ν 1355, 1190, 1174 1096, 1063, 1019 cm^{-1}; ^1H NMR (500.13 MHz, $CDCl_3$) δ 2.45 (s, 3H), 4.24 (m, 1H), 4.29 (m, 1H), 4.52 (m, 1H), 4.62 (m, 1H), 7.36 (d, 2H, J = 8.0 Hz), 7.80 (d, 2H, J = 8.3 Hz); ^{13}C NMR (125.76 MHz, $CDCl_3$) δ 21.6, 68.7 (d, 1C, $^2J_{C\text{-}F}$ = 20.7 Hz), 80.6 (d, 1C, $^1J_{C\text{-}F}$ = 173.3 Hz), 127.9 (2C), 130.0 (2C), 132.6, 145.3.

2. Supplementary figures

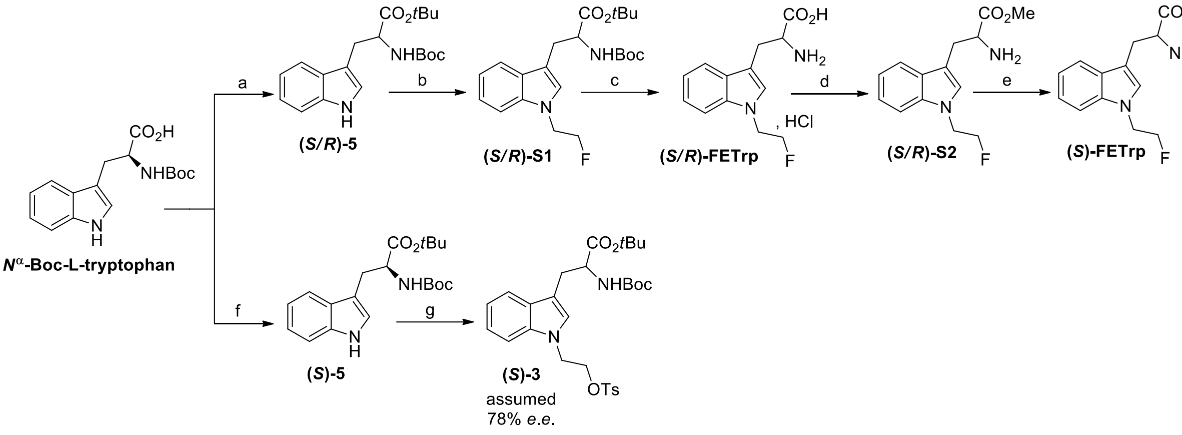

Fig. S1 Synthesis of reference **(*S*)-[^{18}F]FETrp** and precursor **(*S*)-3** according to the protocols of Henrottin et al. (Henrottin et al. 2015 and 2016) and Xin et al. (Xin et al. 2017), respectively. a) *t*-BuOH, DCC, DMAP, CH_2Cl_2, 0°C then rt, 20 h; b) (i) NaH 60 wt%, DMF, 0 °C, 20 min; (ii) 2-fluoroethyl tosylate (**1**), 0 °C, 2 h; c) HCl 6N, 1,4-dioxane, rt, 1.5 h; d) MeOH, TMSCl, rt, 24 h; e) α-chymotrypsin, ammonium formate buffer 100 mM, 37 °C, 5 h; f) (i) Kryptofix 222, anhyd. K_2CO_3, *t*-BuBr, anhyd. DMA, 60 °C, 6 h; (ii) *t*-BuBr, 60 °C, 3 h; g) (i) NaH 60 wt%, DMF, 0 °C, 30 min; (ii) ethylene ditosylate, 1 h, 0 °C then rt, 2 days.

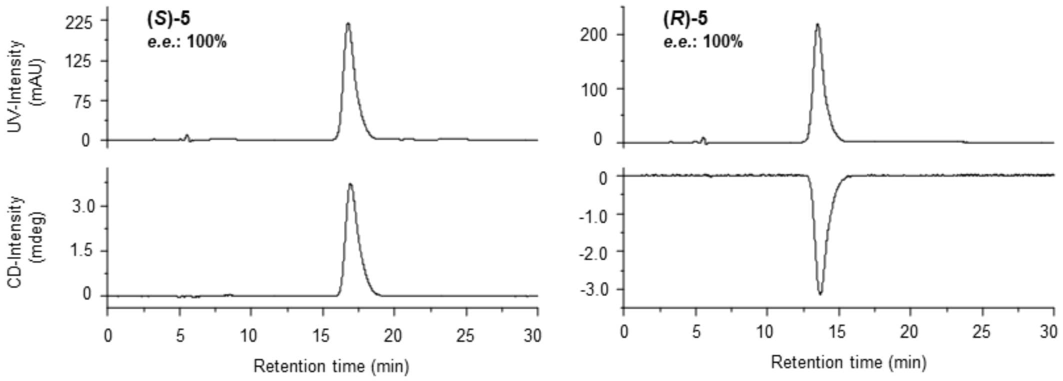

Fig. S2 Representative chromatograms of the chiral UV/circular dichroism-HPLC analyses of compound **5**. Column: CHIRALPAK IA; Mobile phase composition: *n*-hexane/*i*-PrOH (90/10, v/v); UV detection at λ = 280 nm; CD detection at λ = 230 nm.

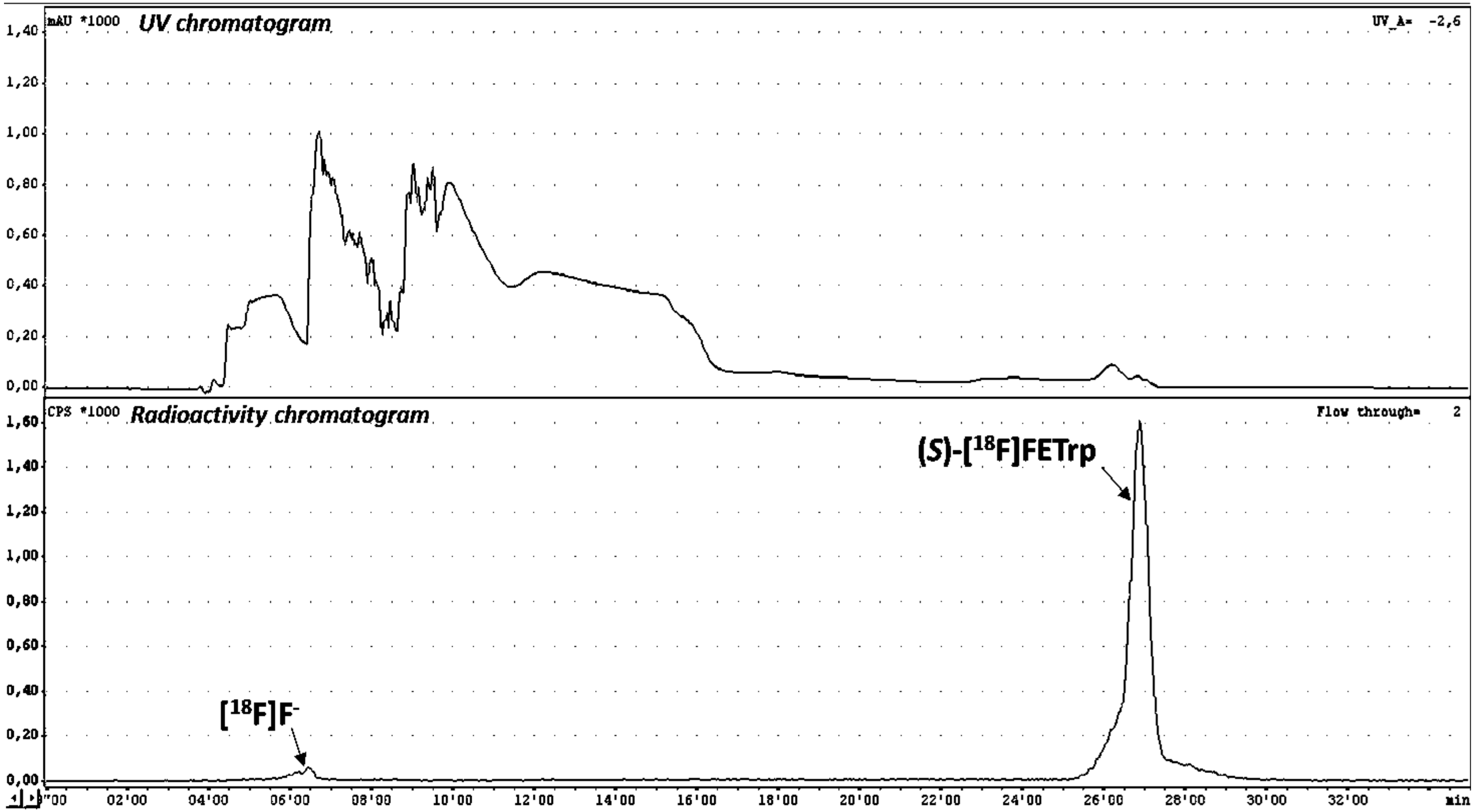

Fig. S3 UV and radioactivity chromatograms of the semi-preparative purification of **(S)-[^{18}F]FETrp**. Symmetryprep C18 column (300 x 7.8 mm; 7 µm; Waters) was eluted with a mixture of H_2O/MeCN/TFA (90/10/0.01, v/v/v) in isocratic mode at λ = 254 nm and with a flow rate of 1.5 mL/min for one minute and then 2.5 mL/min.

3. ^{1}H and ^{13}C NMR spectra of all synthesised compounds

^{1}H (top) and ^{13}C (down) NMR spectra of compound **1** in $CDCl_3$

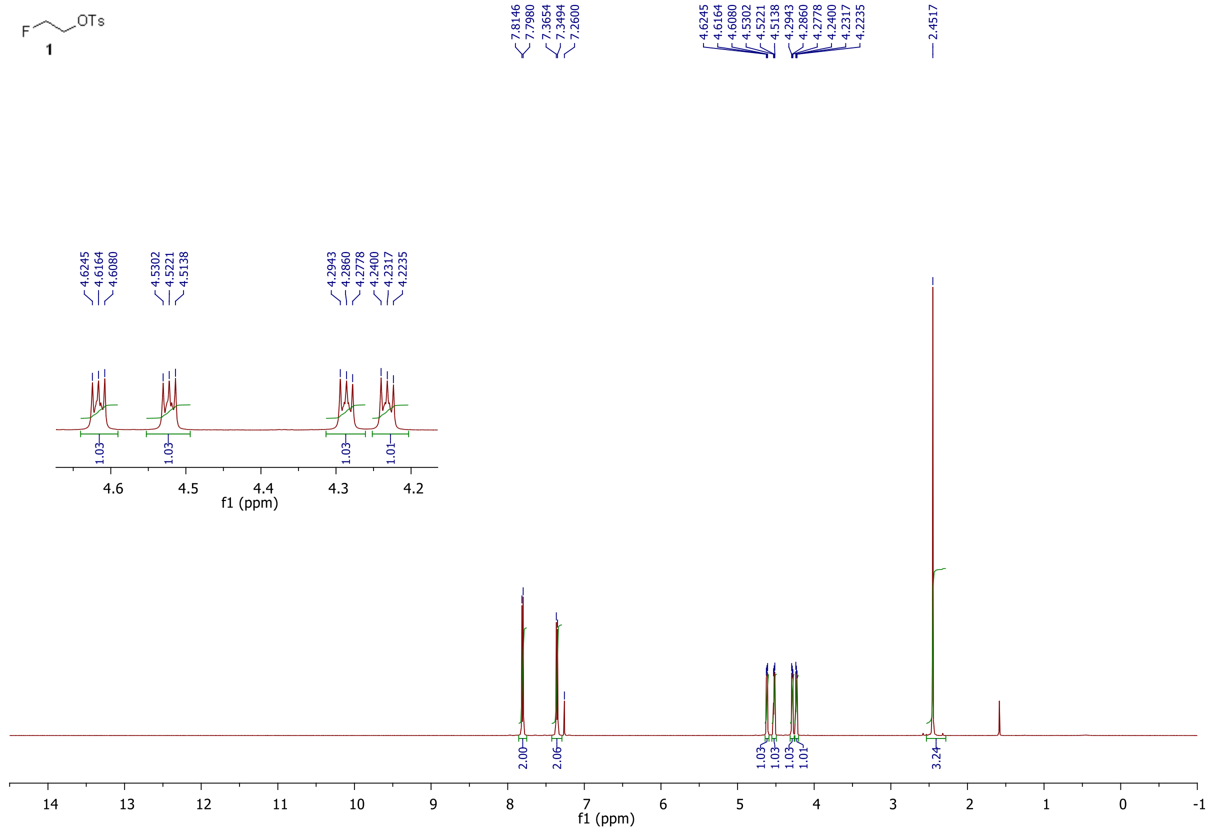

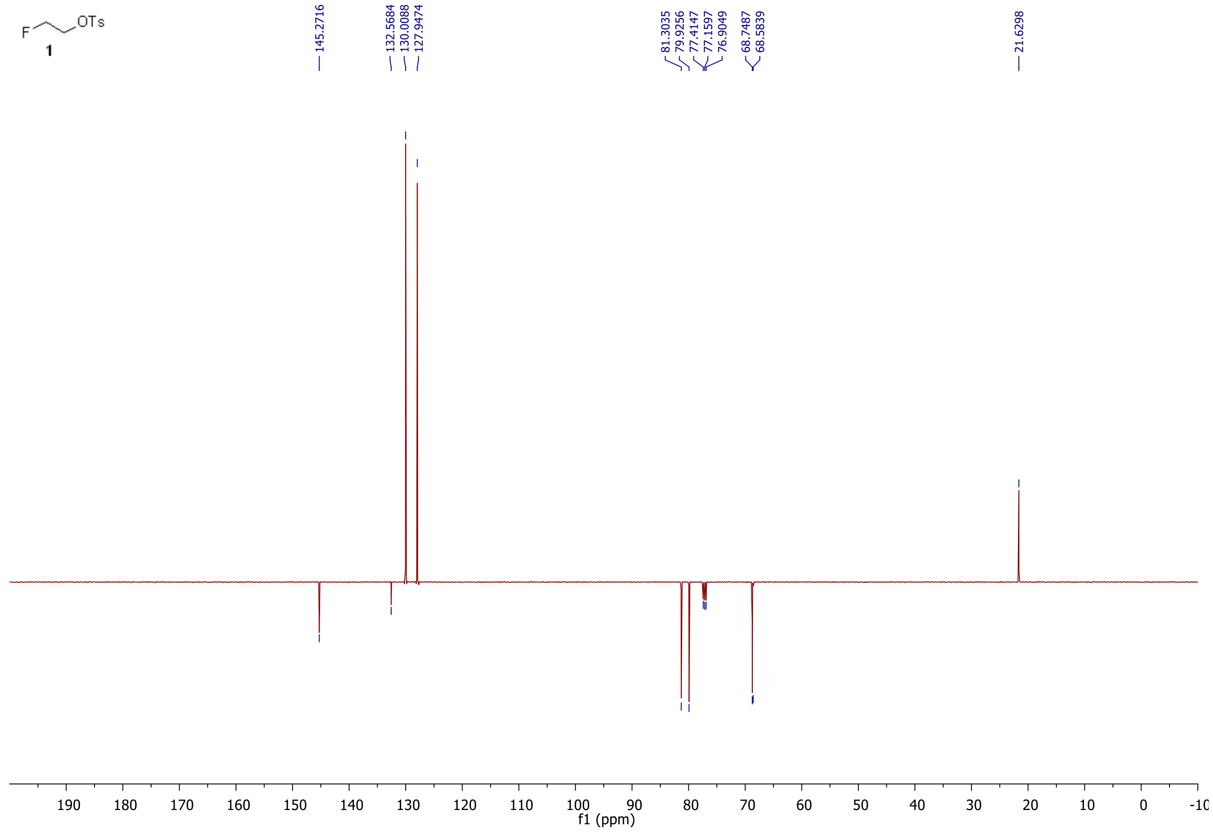

^{1}H (top) and ^{13}C (down) NMR spectra of compound **(*S*)-5** in $CDCl_3$

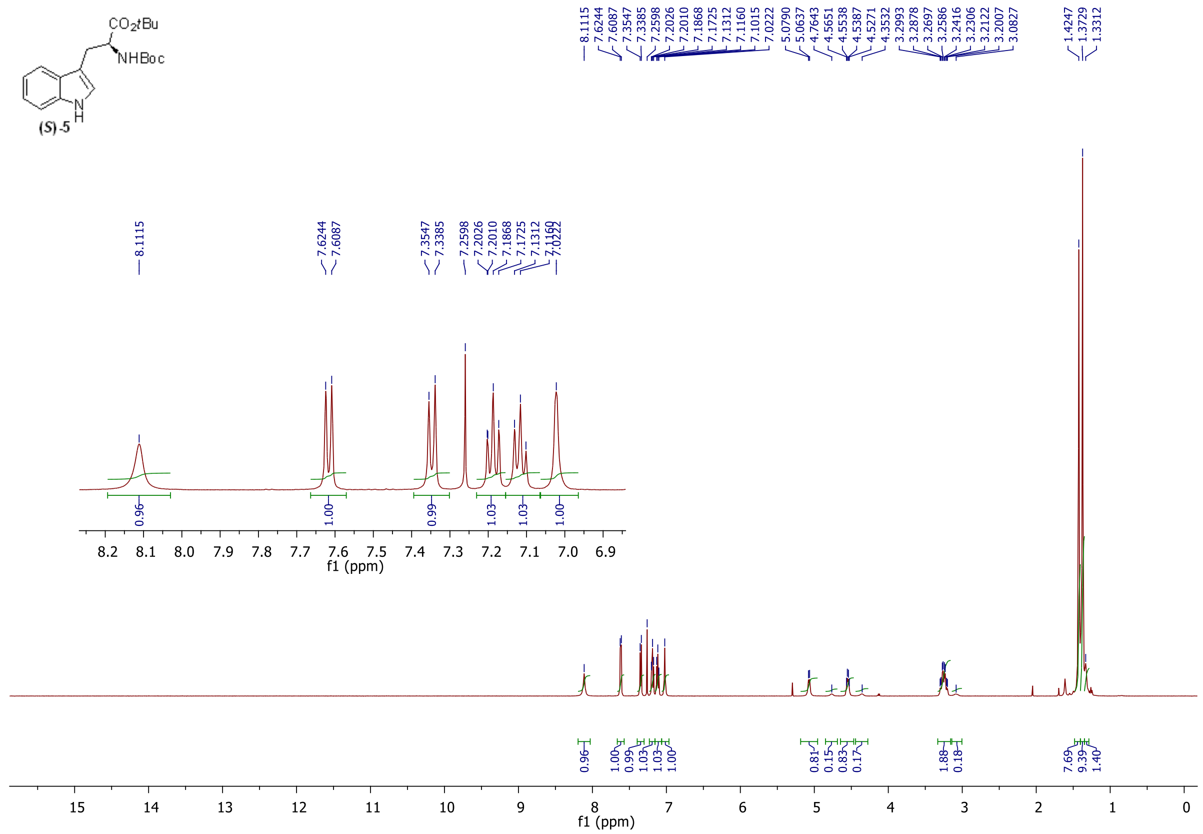

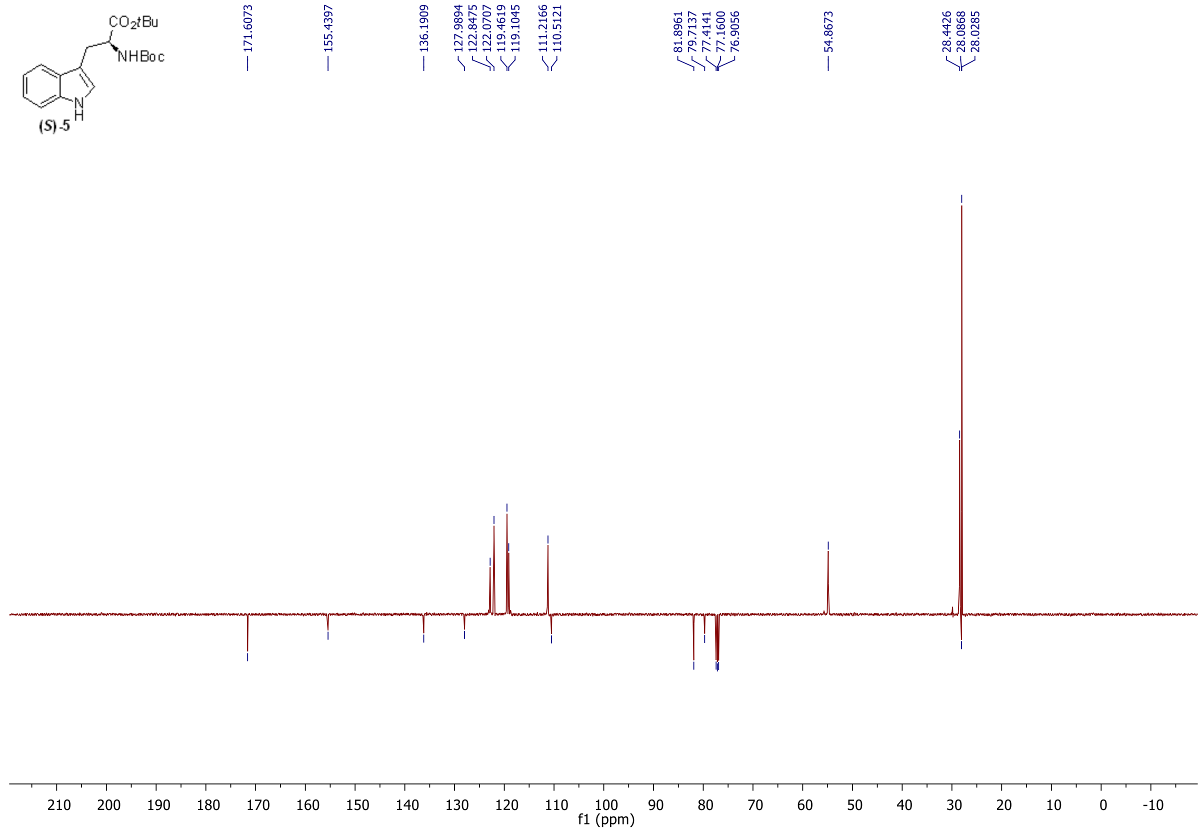

^{1}H (top) and ^{13}C (down) NMR spectra of compound **(*R*)-5** in $CDCl_3$

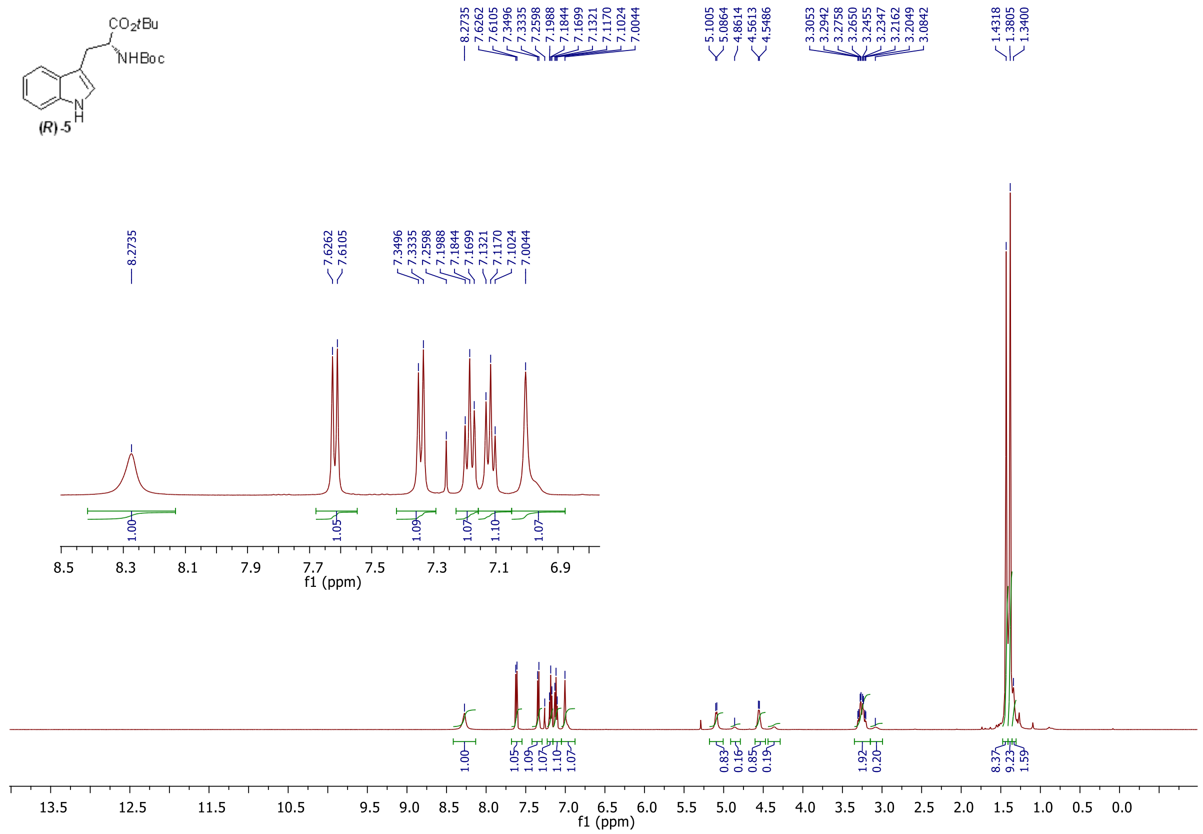

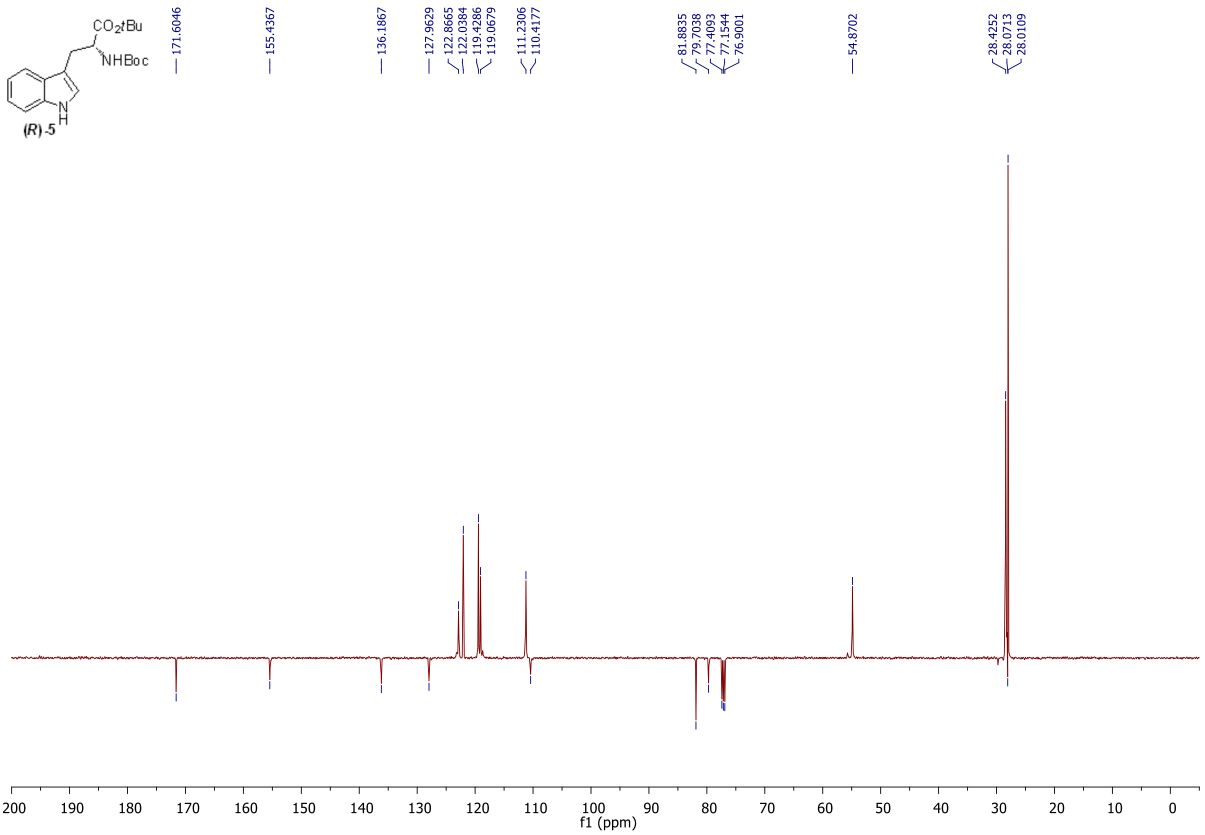

^{1}H (top) and ^{13}C (down) NMR spectra of compound **(*S*)-4** in $CDCl_3$

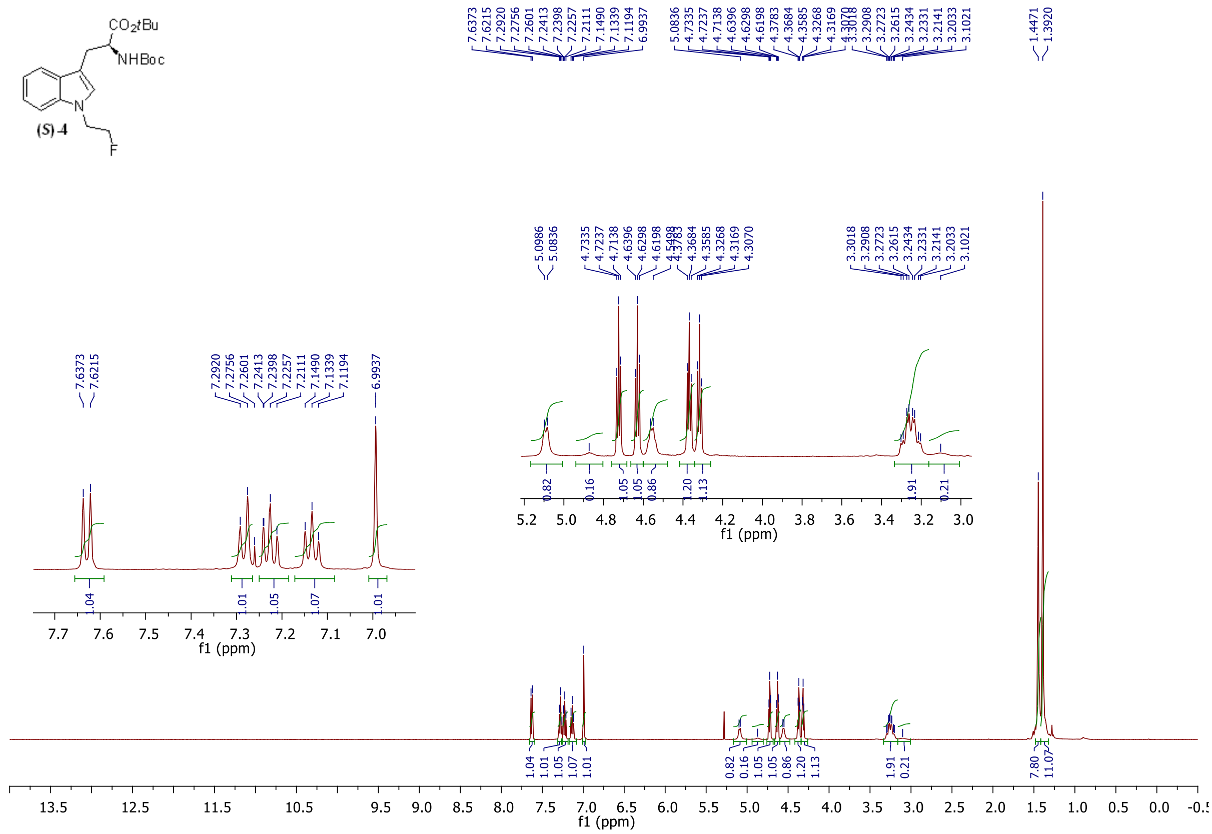

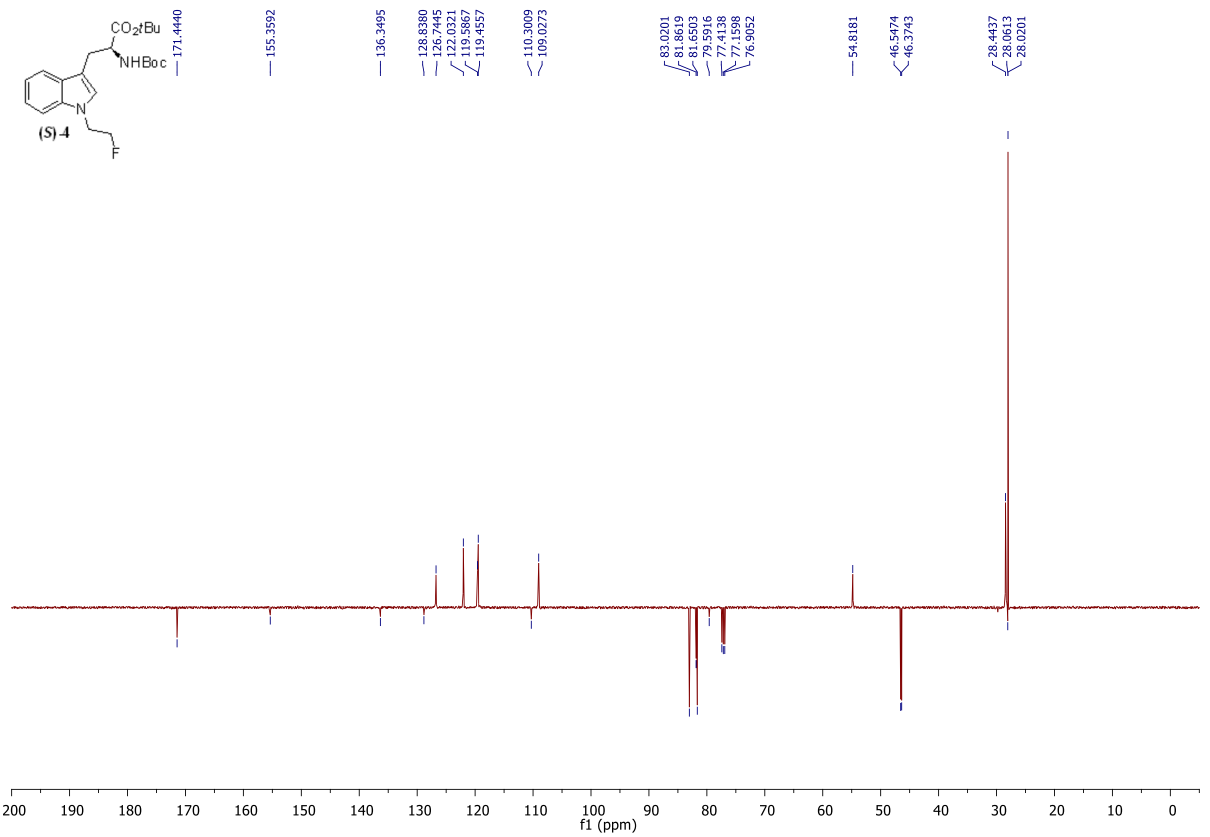

^{1}H (top) and ^{13}C (down) NMR spectra of compound **(*R*)-4** in $CDCl_3$

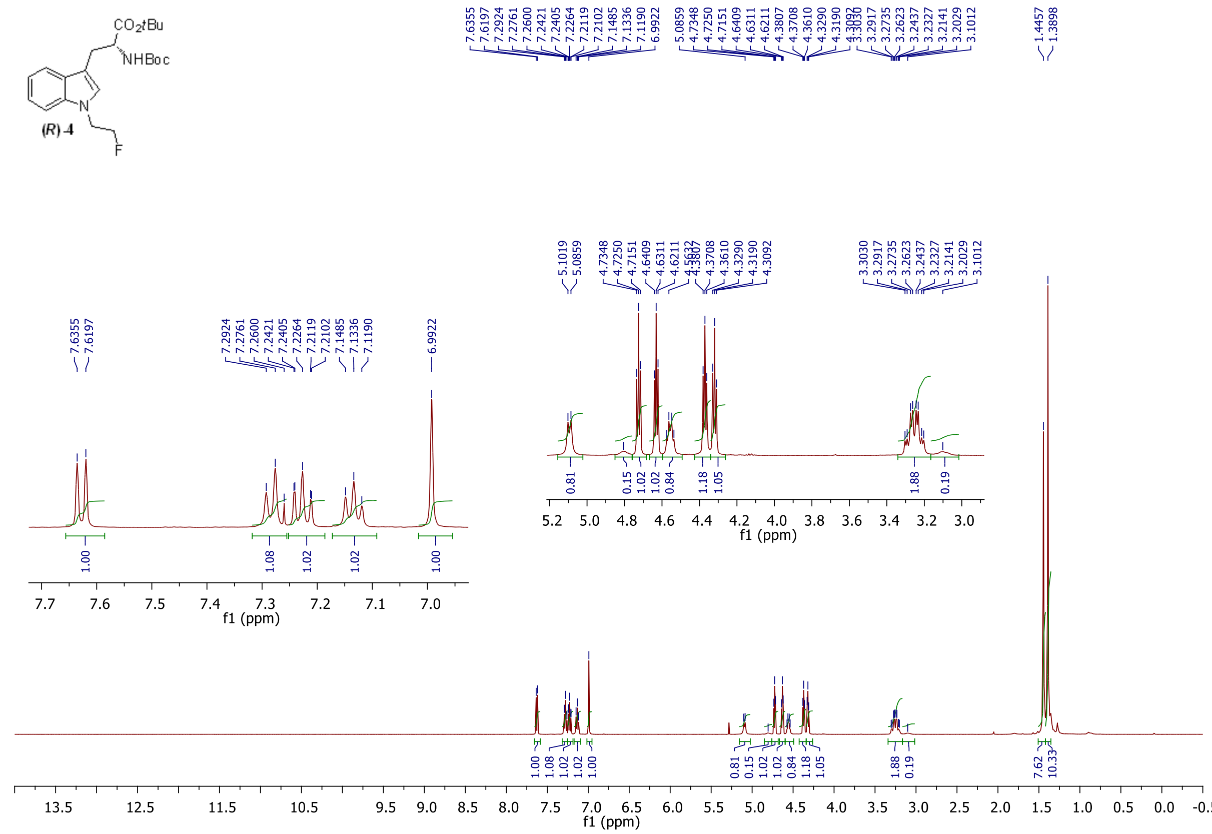

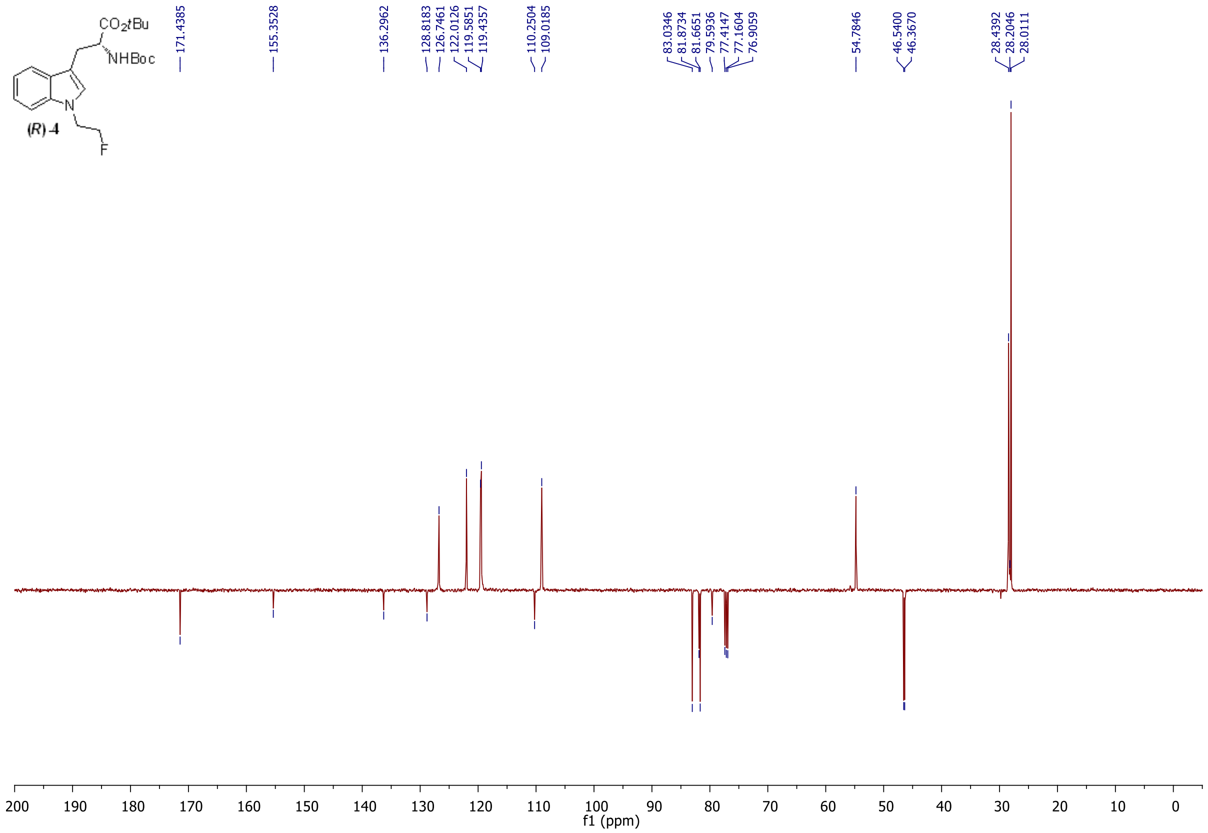

^{19}F NMR spectra of compounds **(*S*)-4** (top) and **(*R*)-4** (down) in $CDCl_3$ using trifluorotoluene as internal reference.

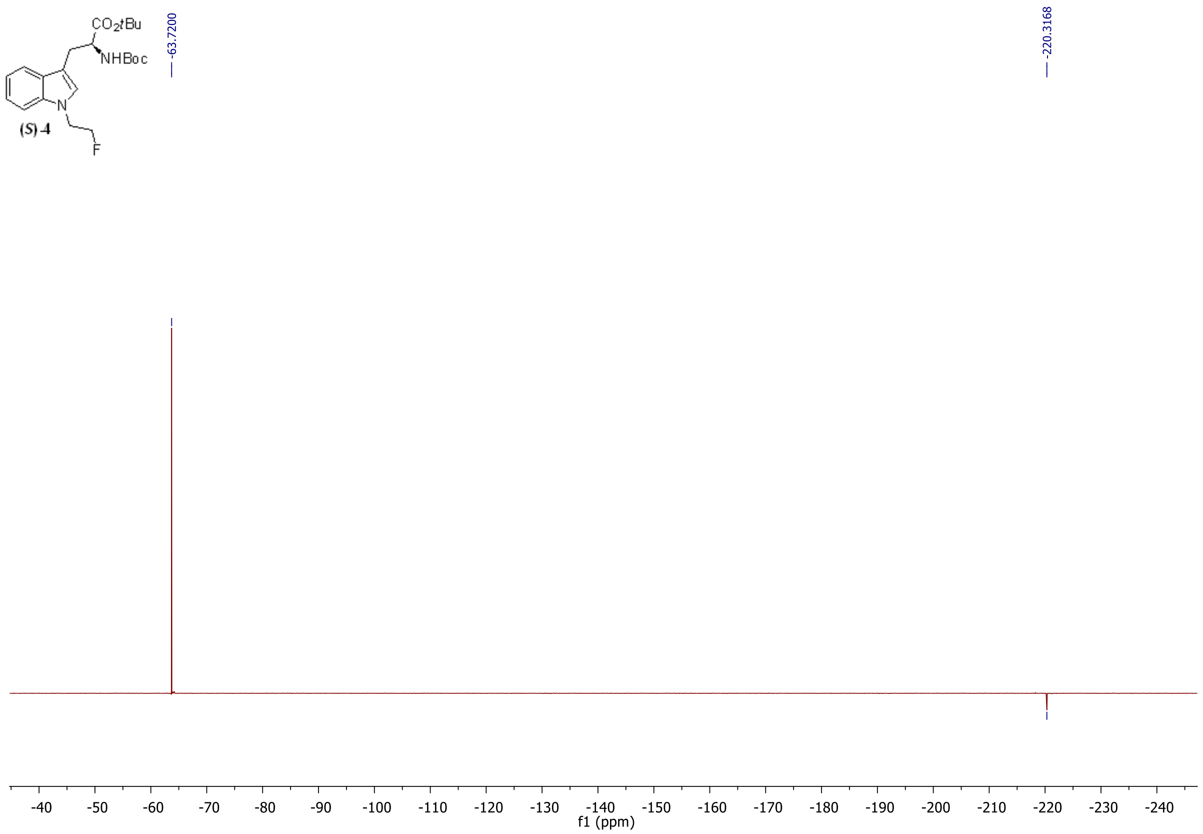

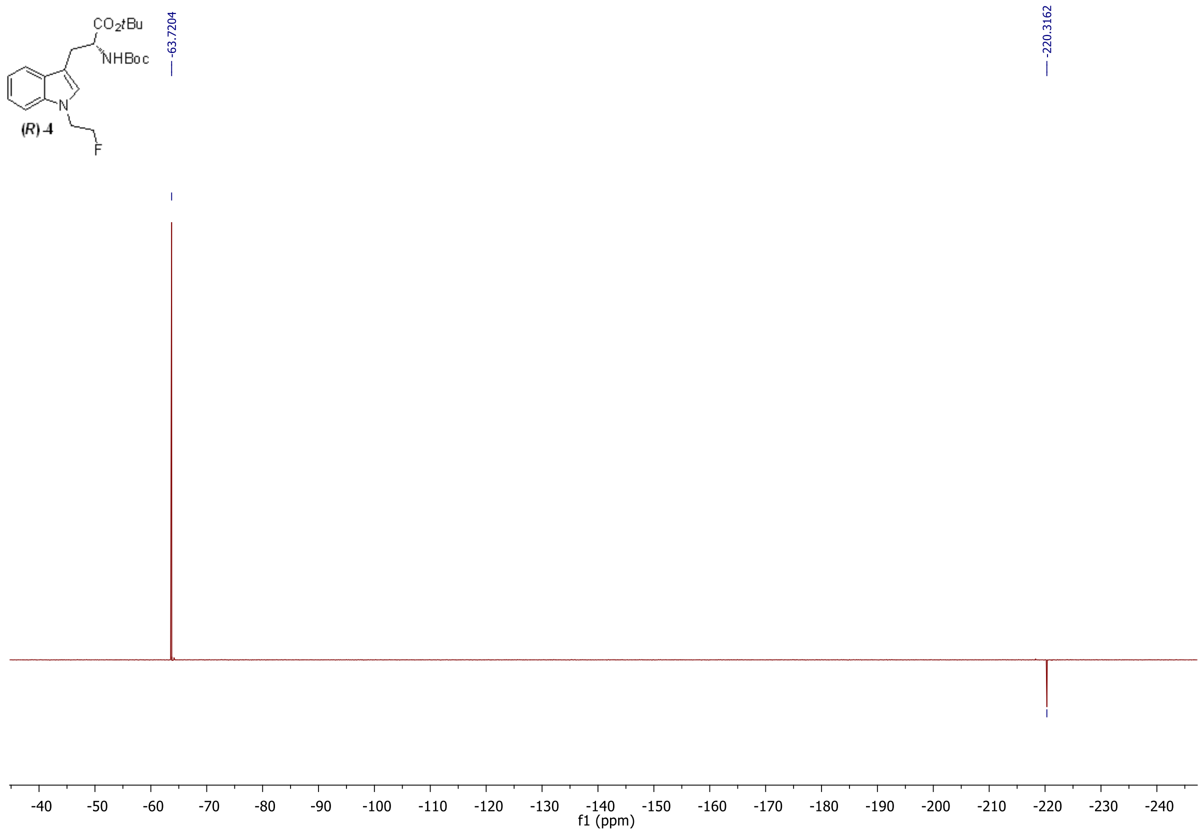

^{1}H (top) and ^{13}C (down) NMR spectra of compound **(*S*)-6** in $CDCl_3$

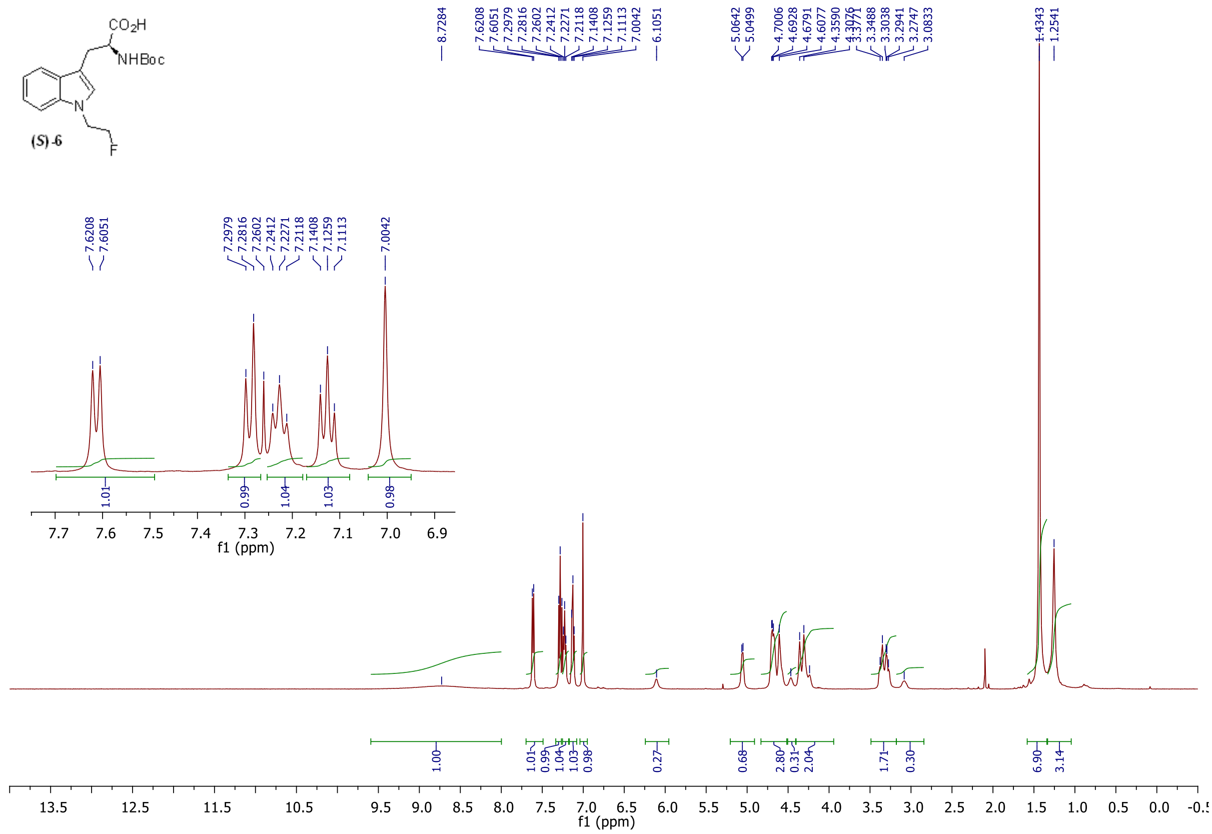

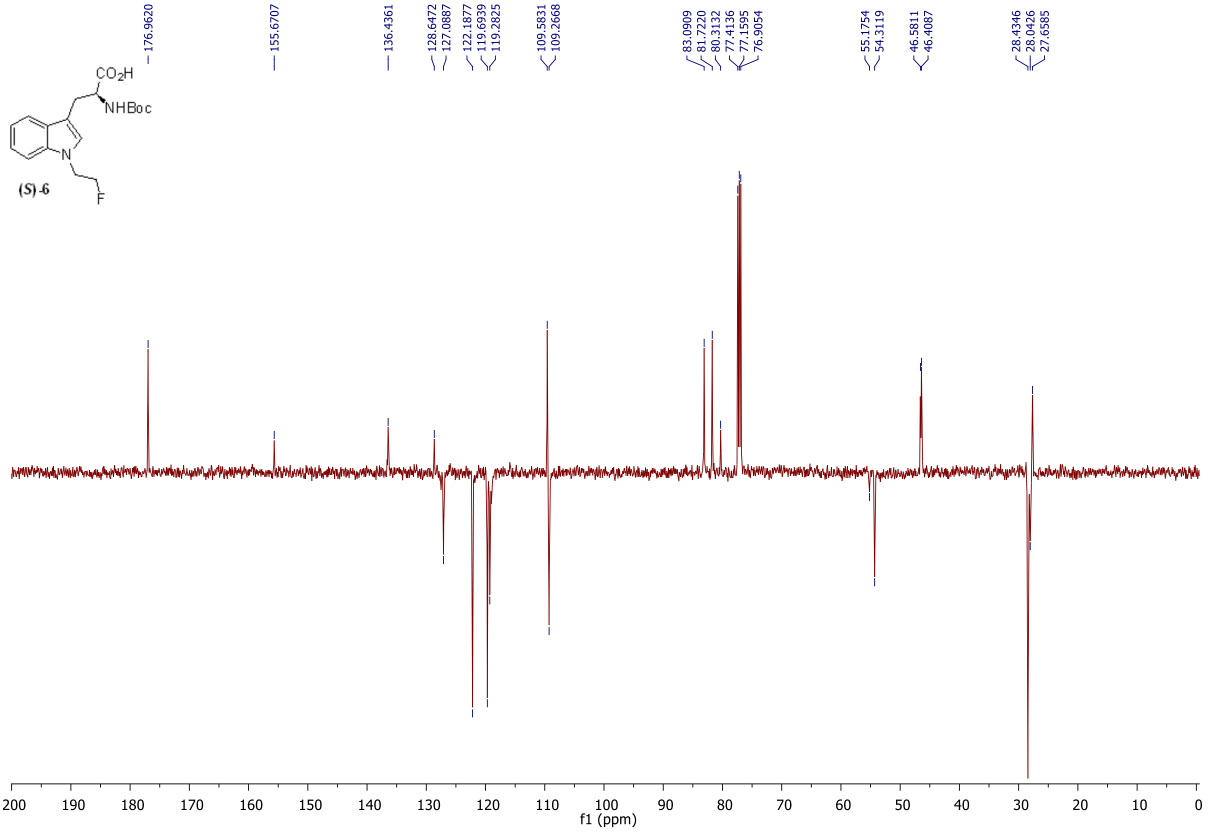

^{1}H (top) and ^{13}C (down) NMR spectra of compound **(*R*)-6** in $CDCl_3$

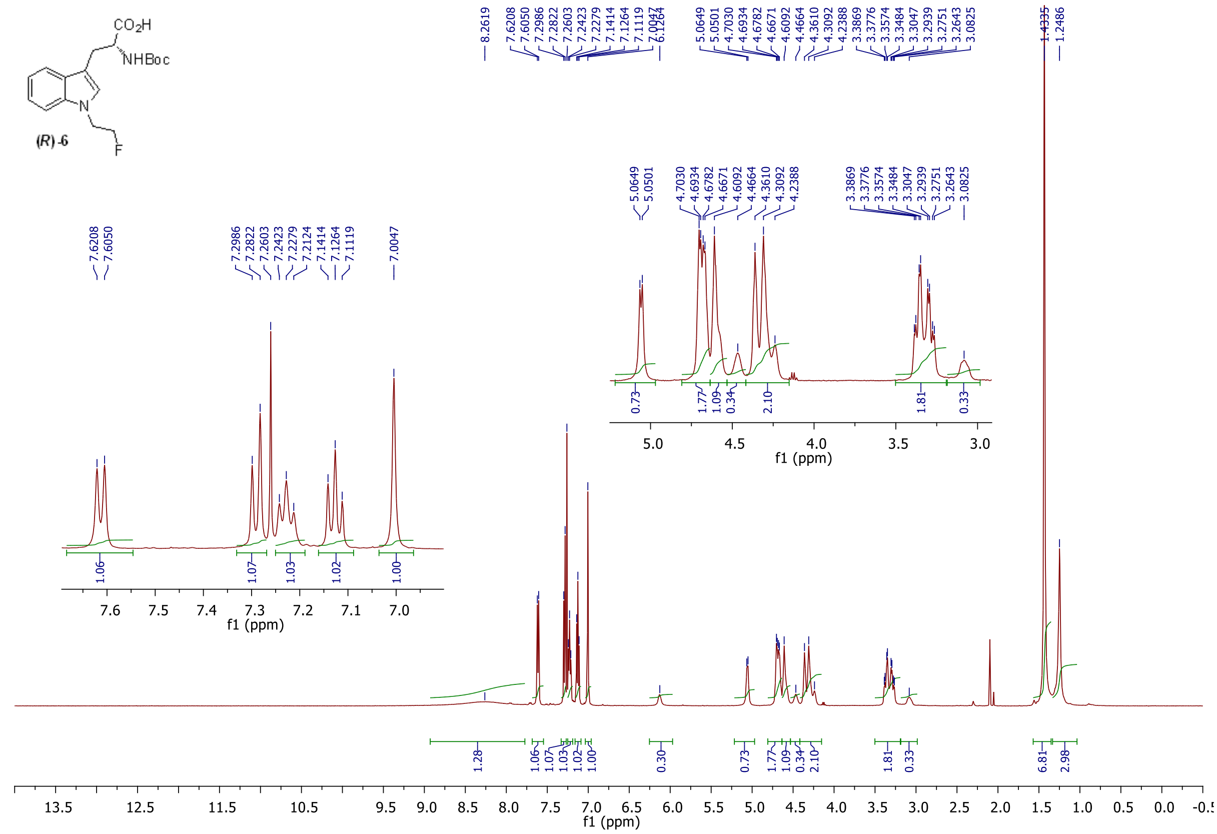

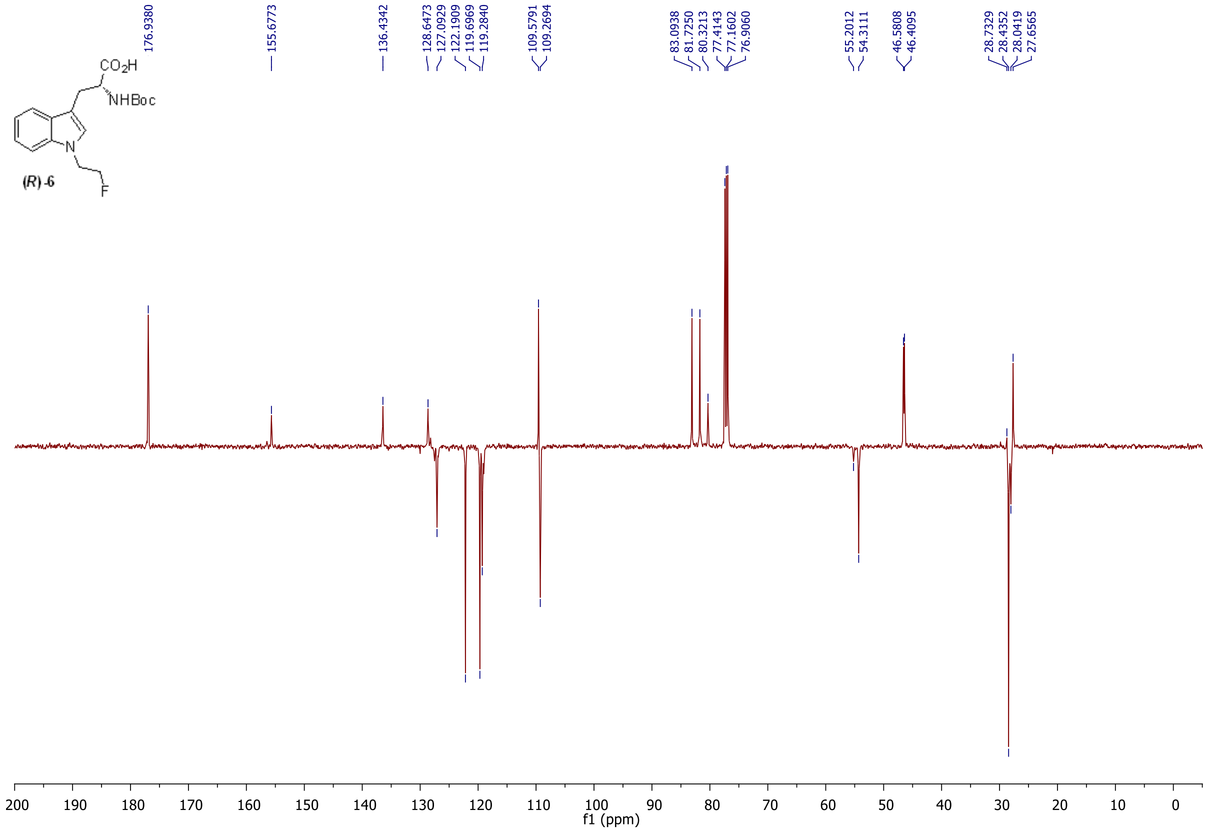

^{19}F NMR spectra of compounds **(*S*)-6** (top) and **(*R*)-6** (down) in $CDCl_3$ using trifluorotoluene as internal reference

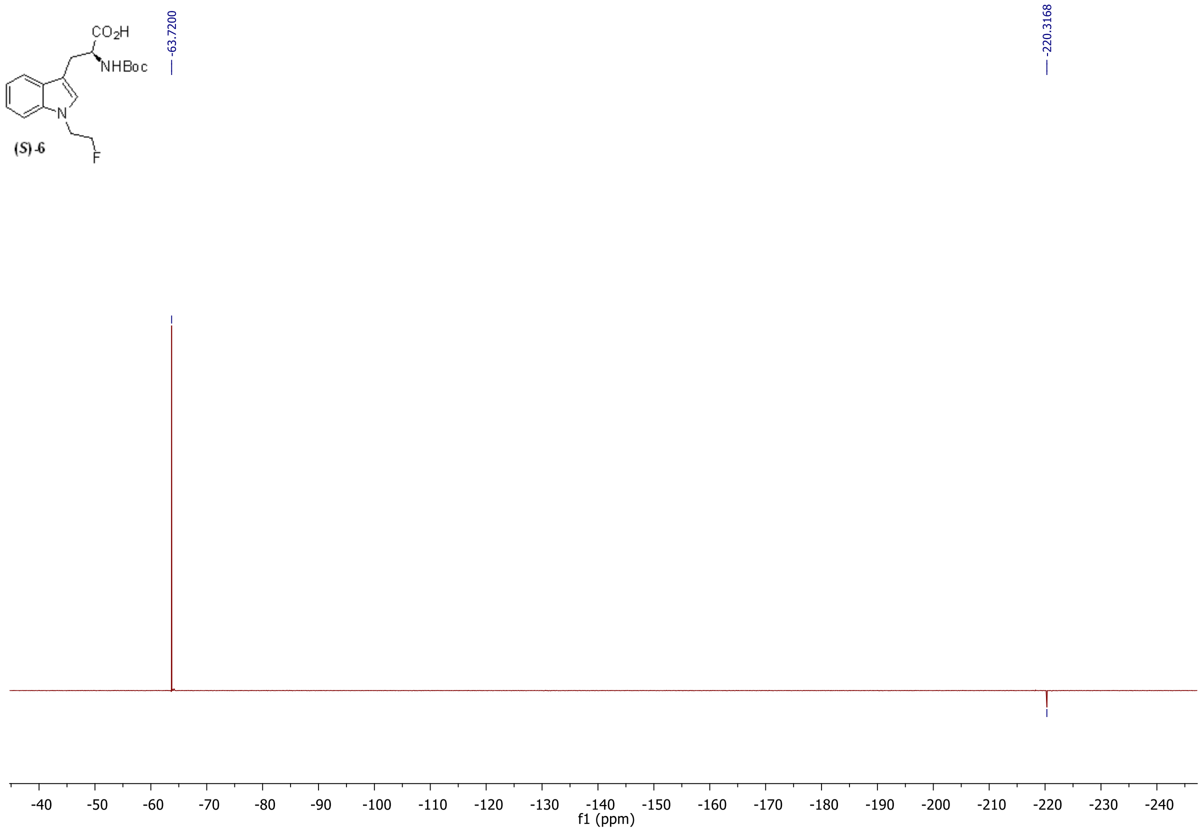

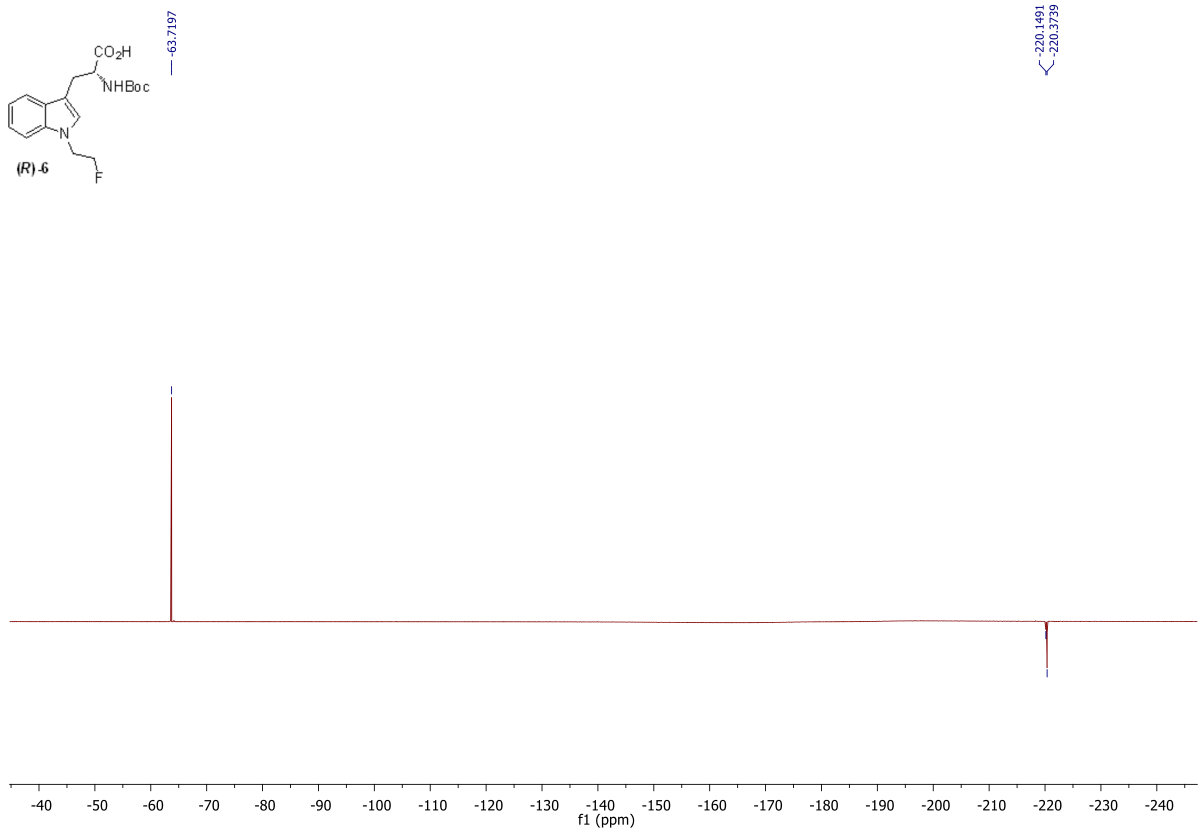

^{1}H (top) and ^{13}C (down) NMR spectra of compound **(*S*)-FETrp** in D_2O

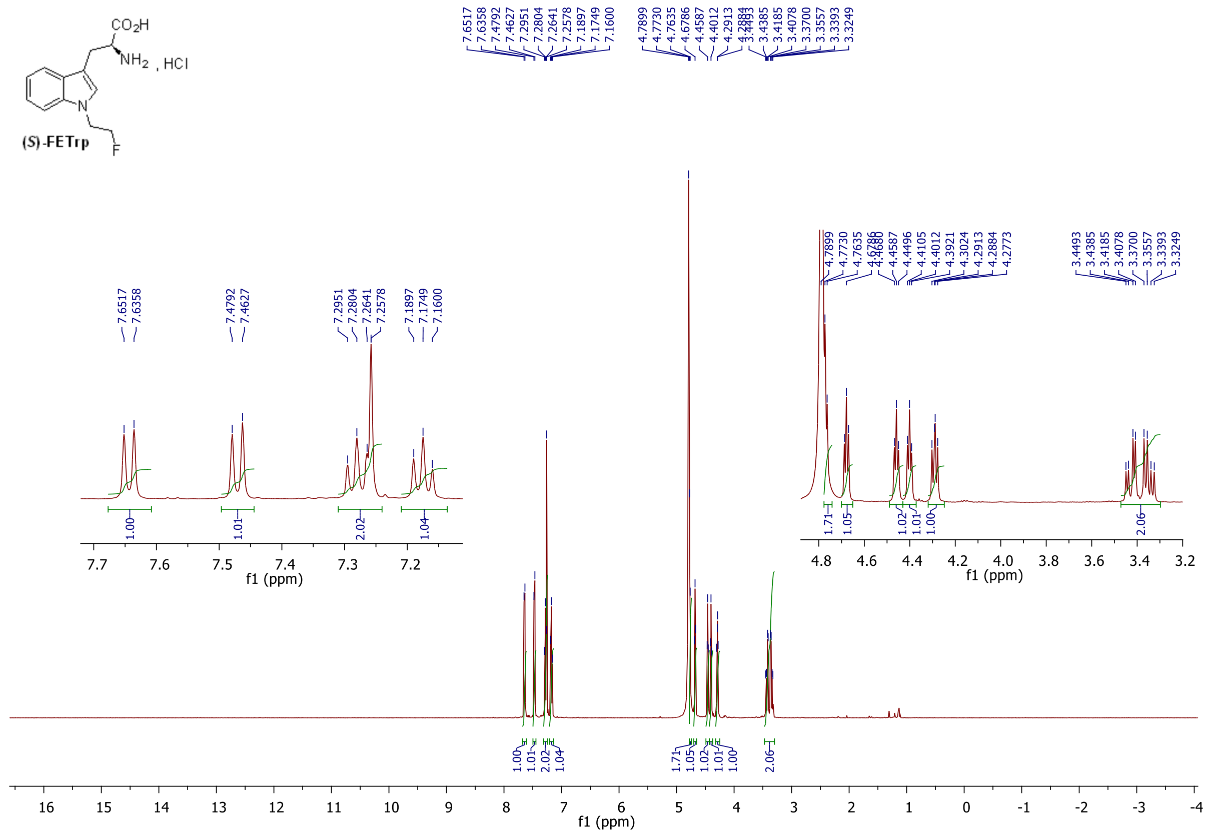

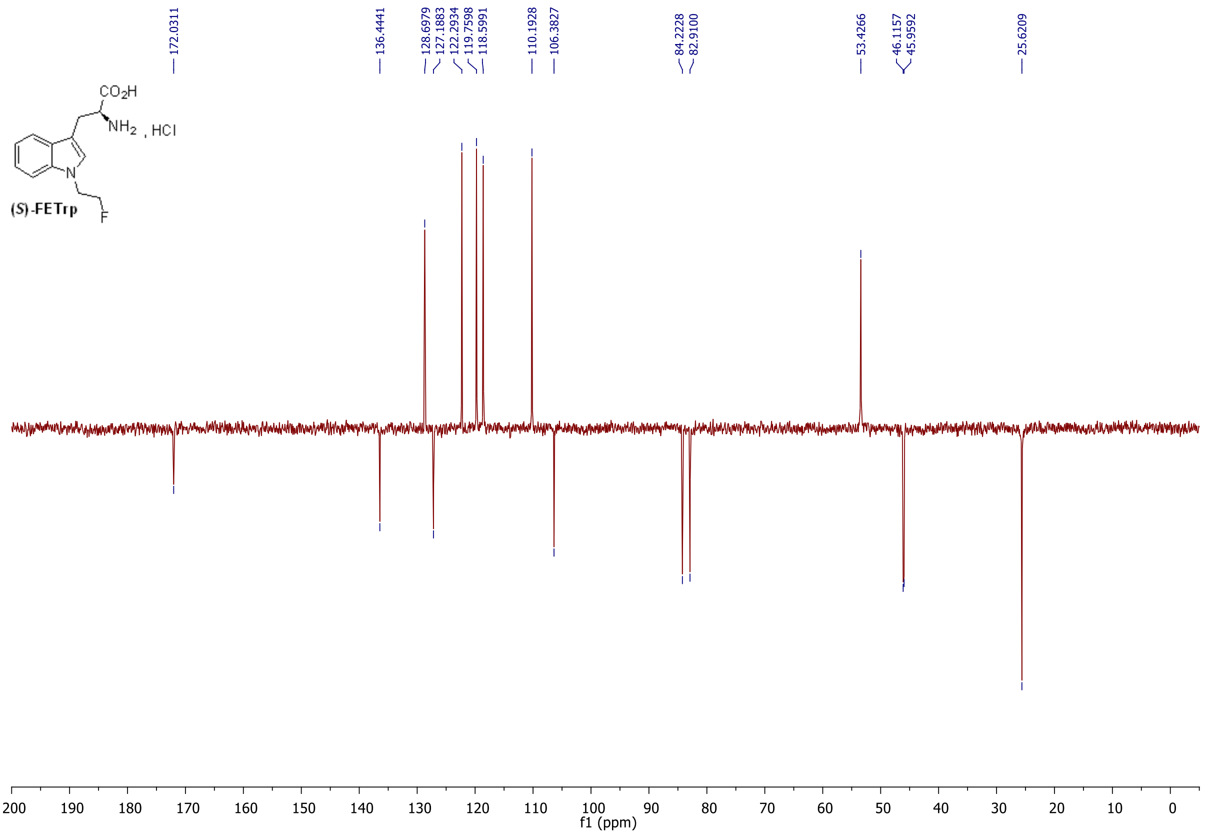

^{1}H (top) and ^{13}C (down) NMR spectra of compound **(*R*)-FETrp** in D_2O

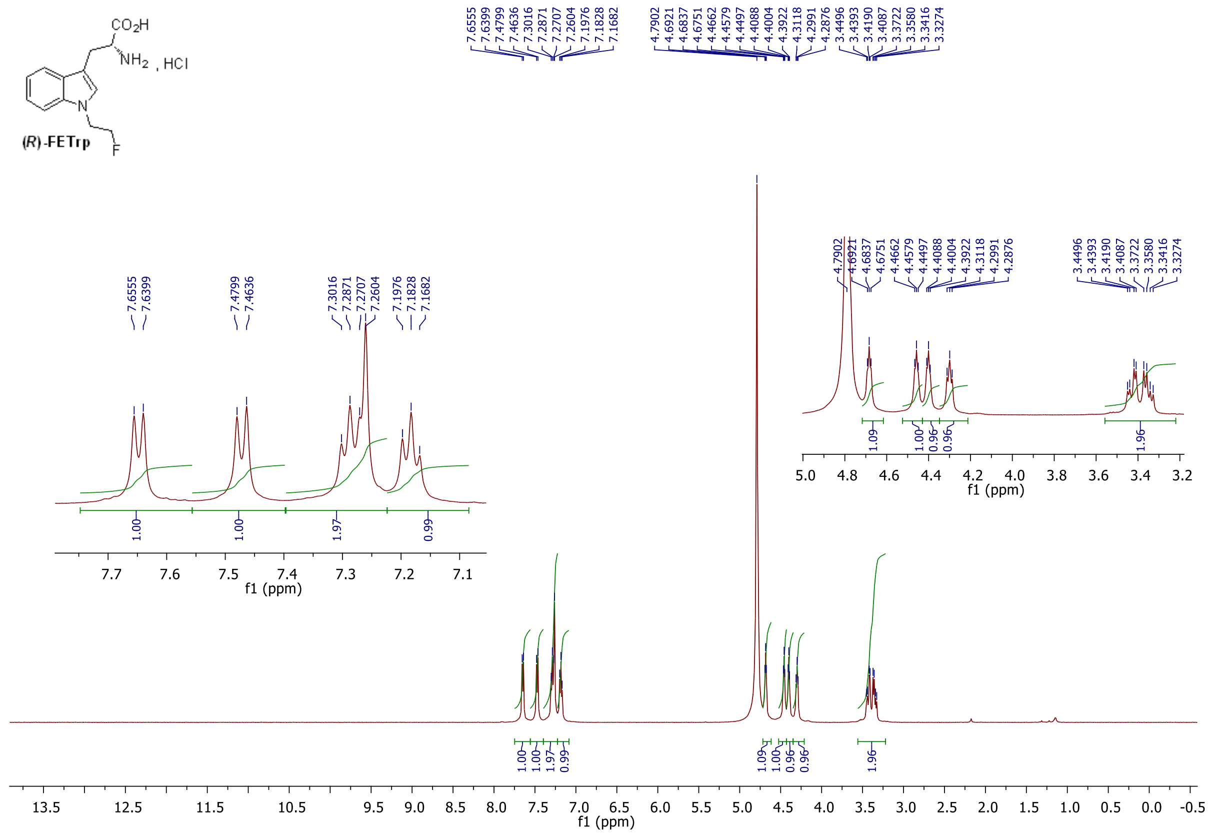

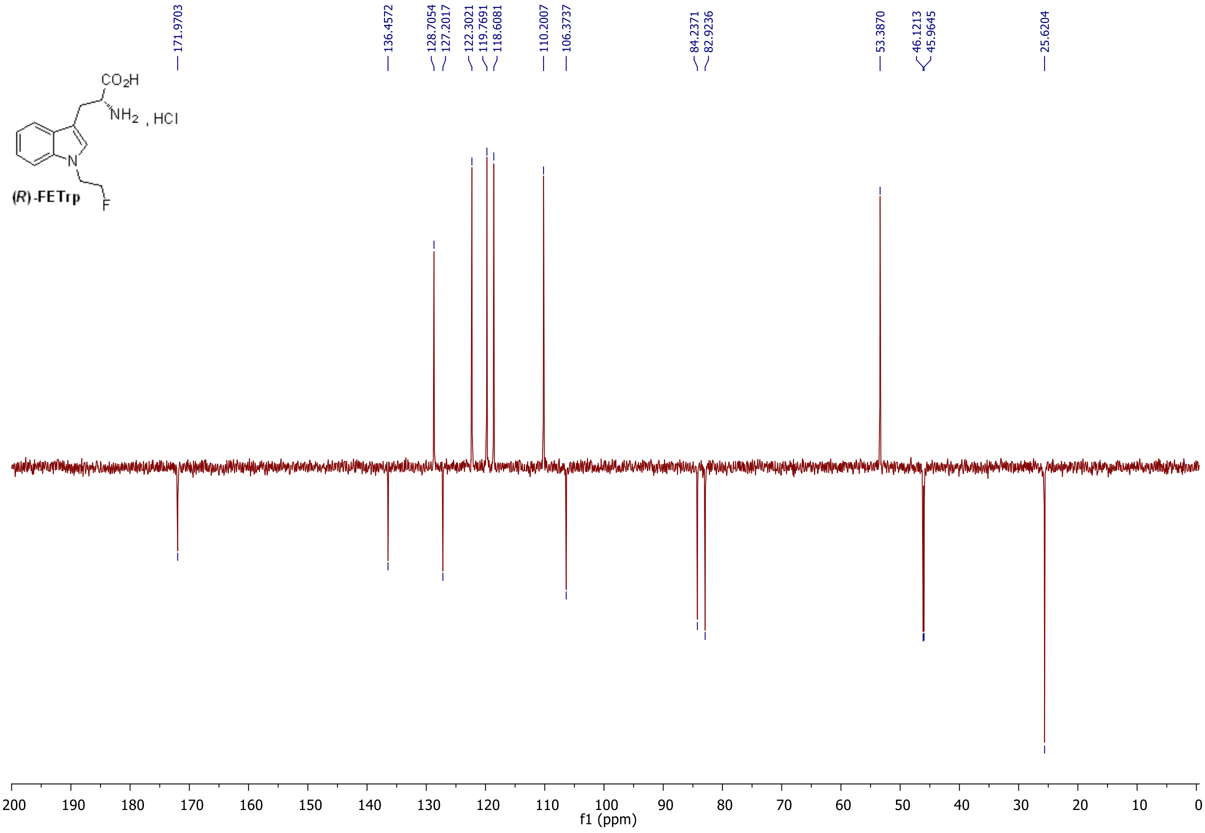

^{19}F NMR spectra of compounds **(*S*)-FETrp** (top) and **(*R*)-FETrp** (down) in D_2O using trifluorotoluene as internal reference

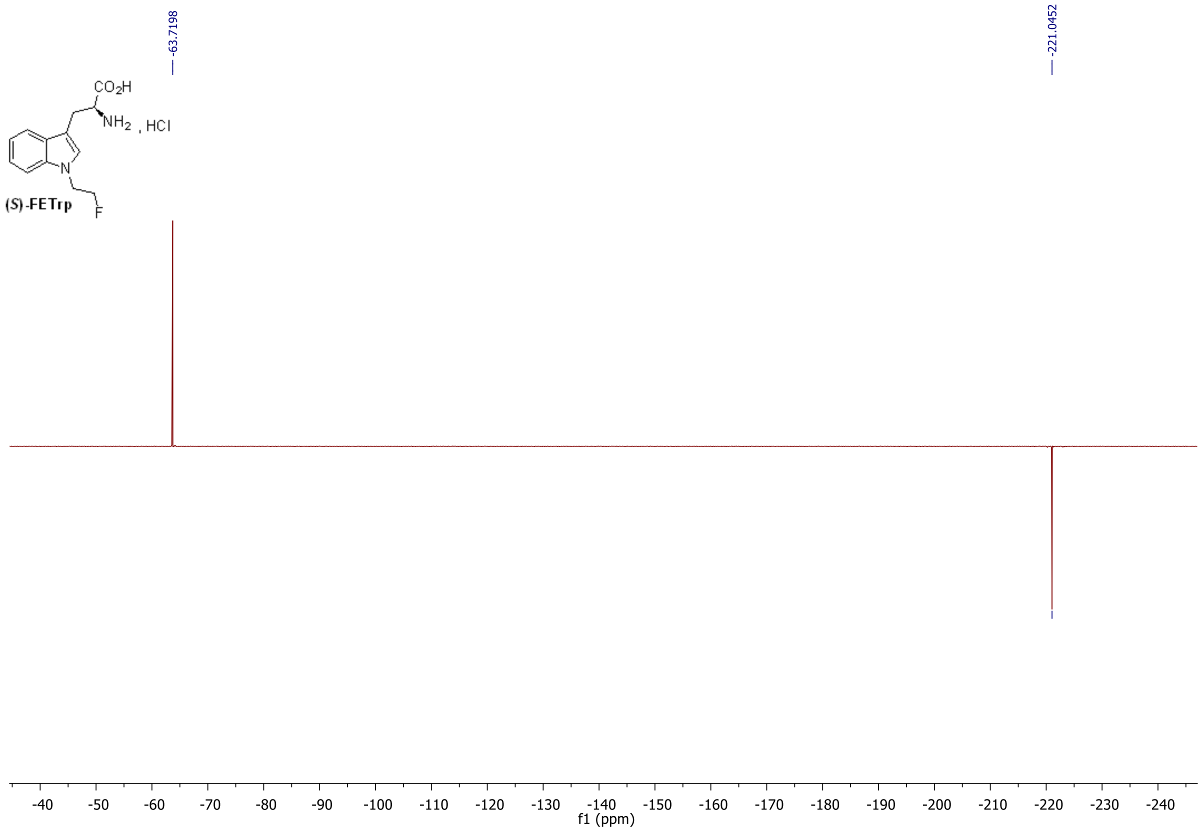

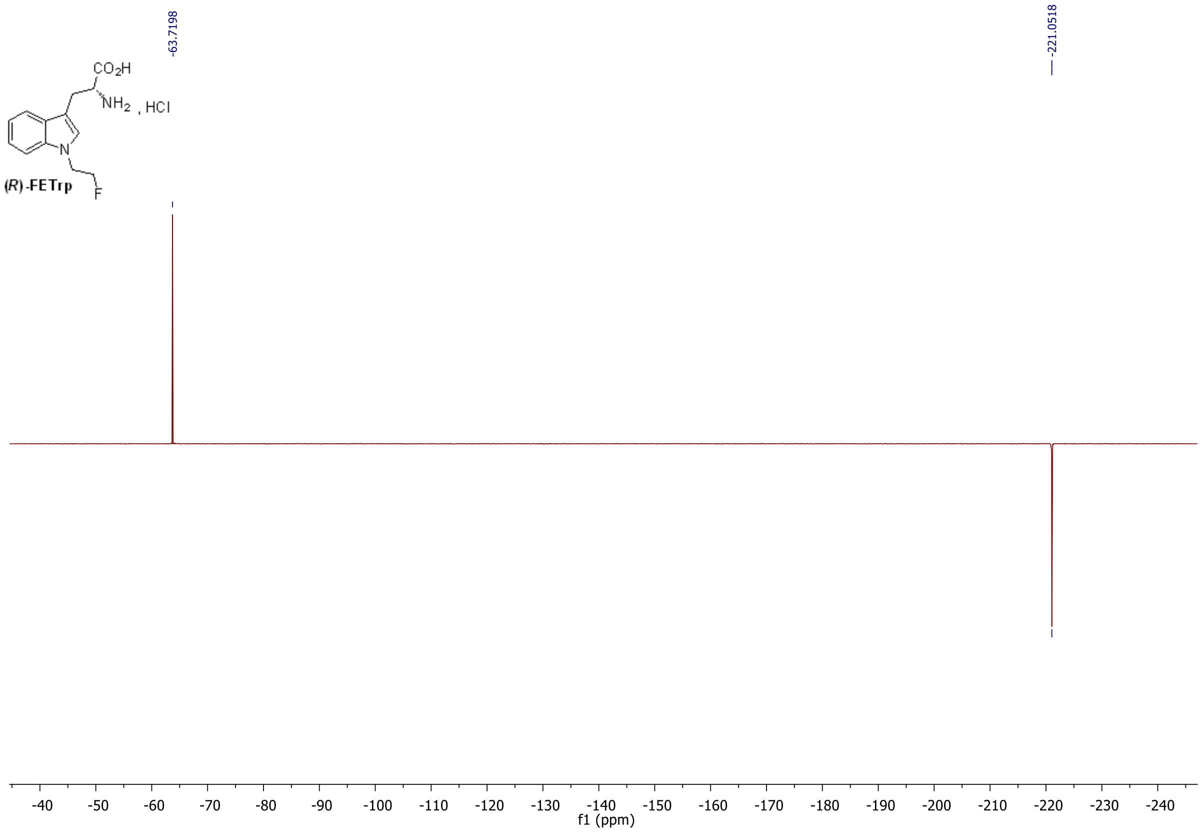

^{1}H (top) and ^{13}C (down) NMR spectra of compound **(*S*)-7** in $CDCl_3$

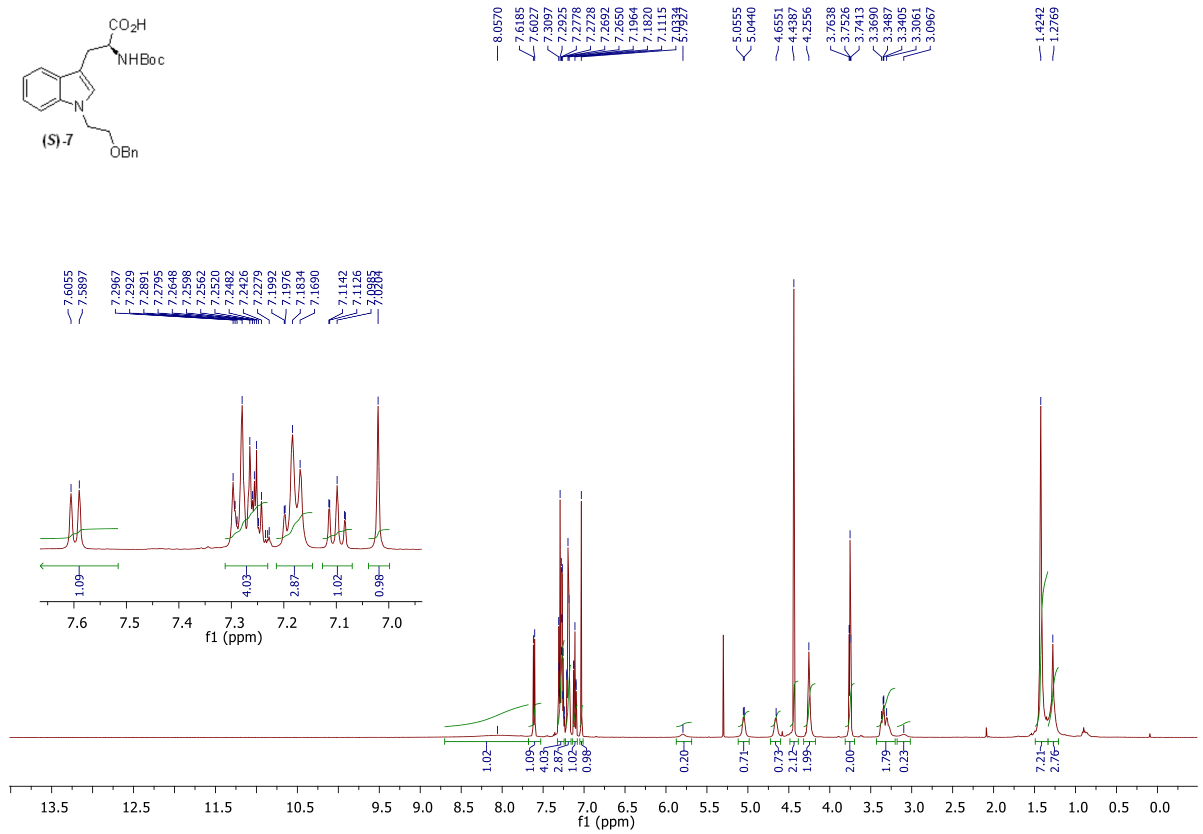

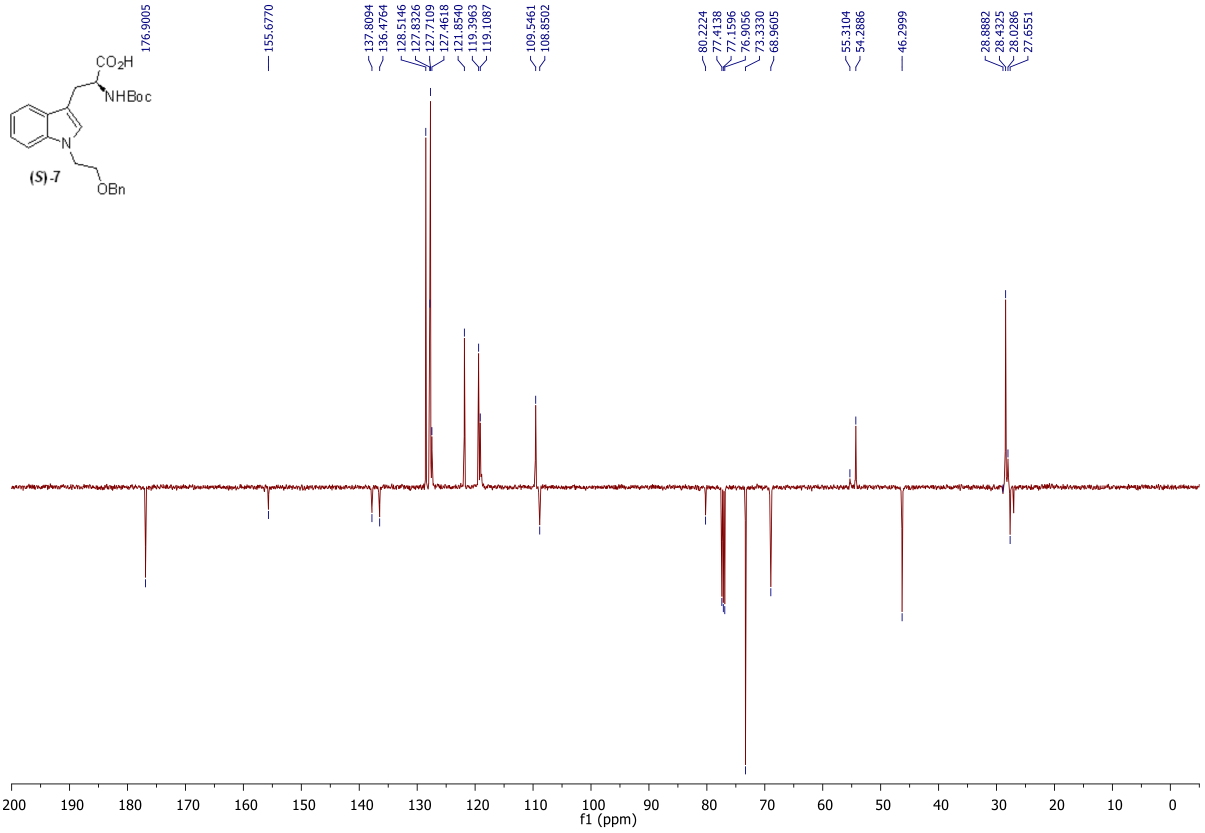

^{1}H (top) and ^{13}C (down) NMR spectra of compound ***(R)*-7** in $CDCl_3$

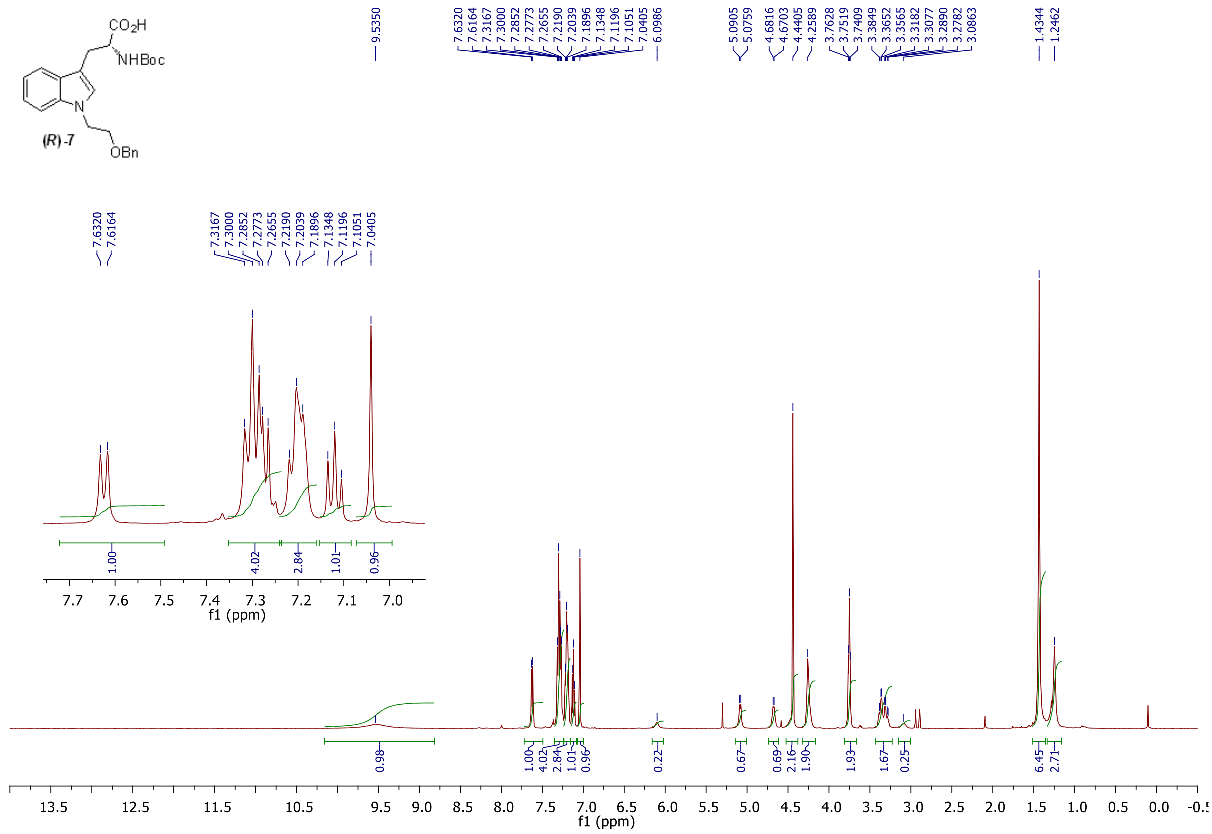

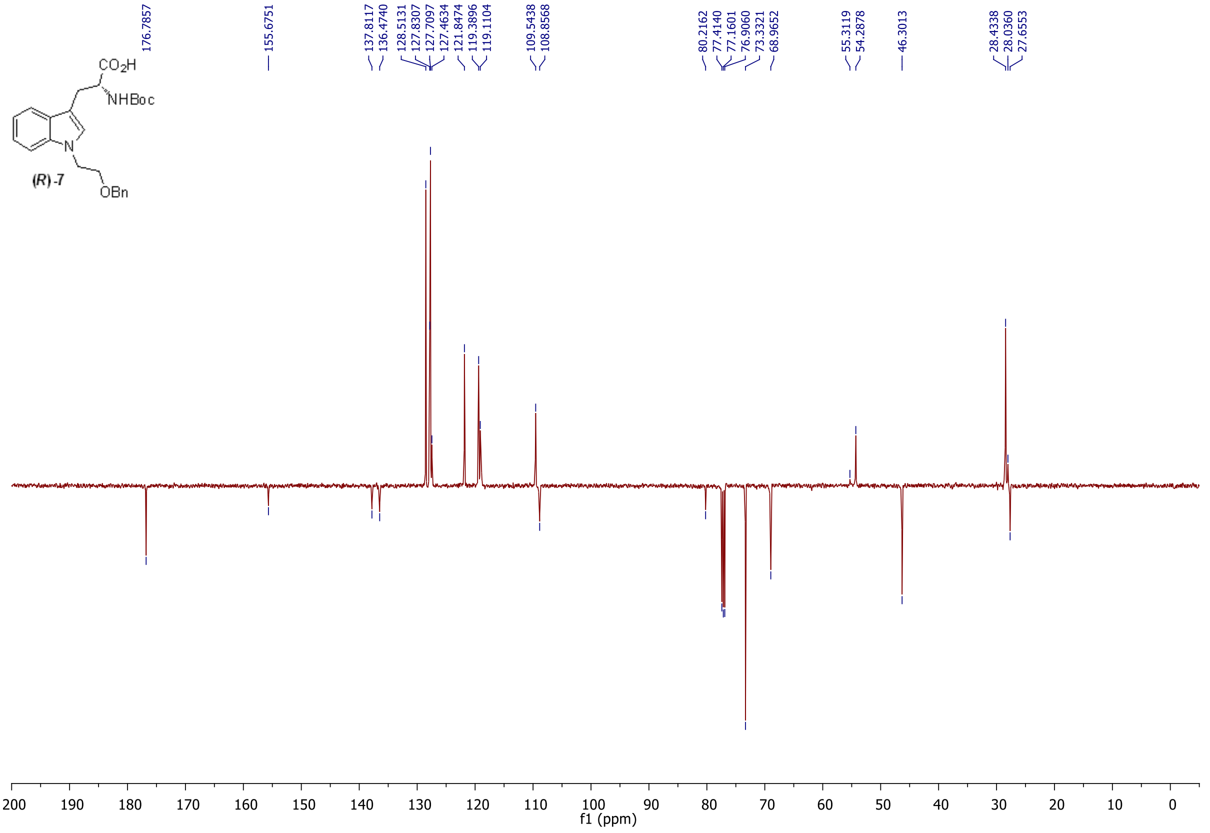

^{1}H (top) and ^{13}C (down) NMR spectra of compound **(*S*)-8** in $CDCl_3$

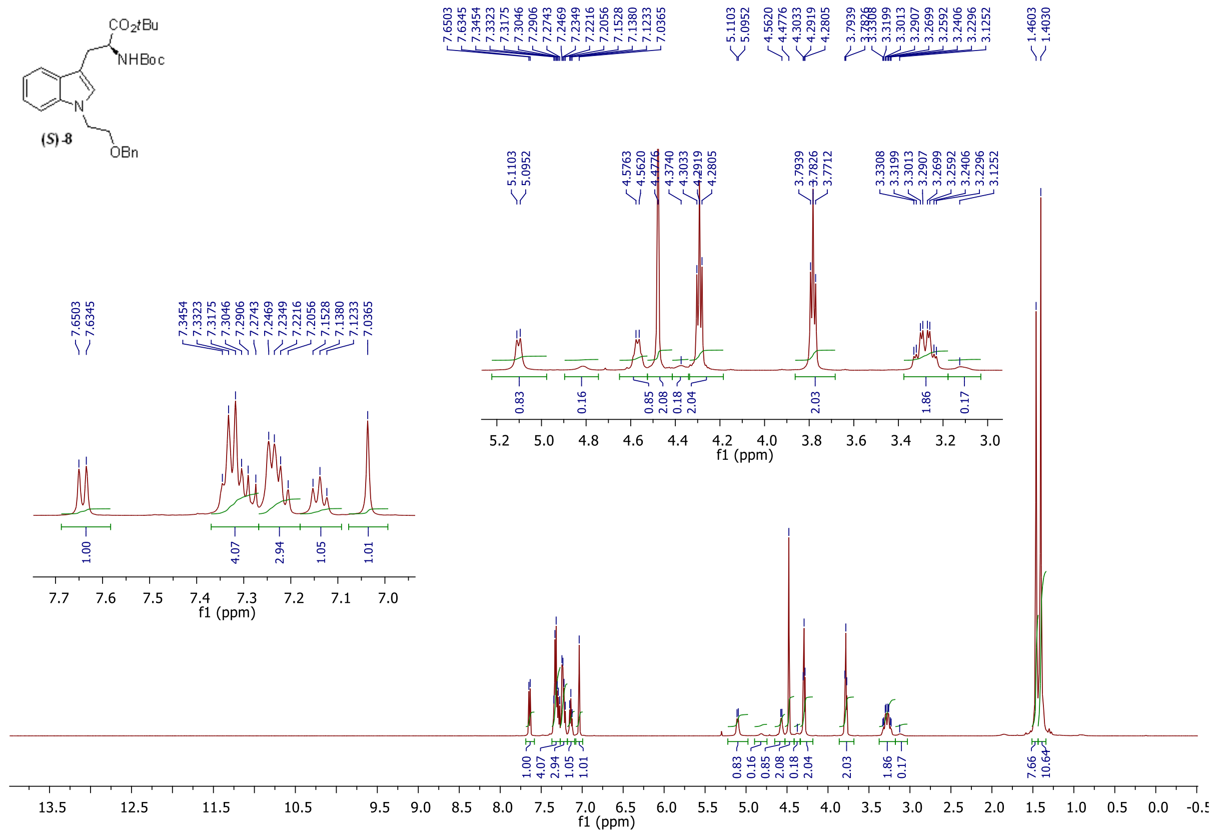

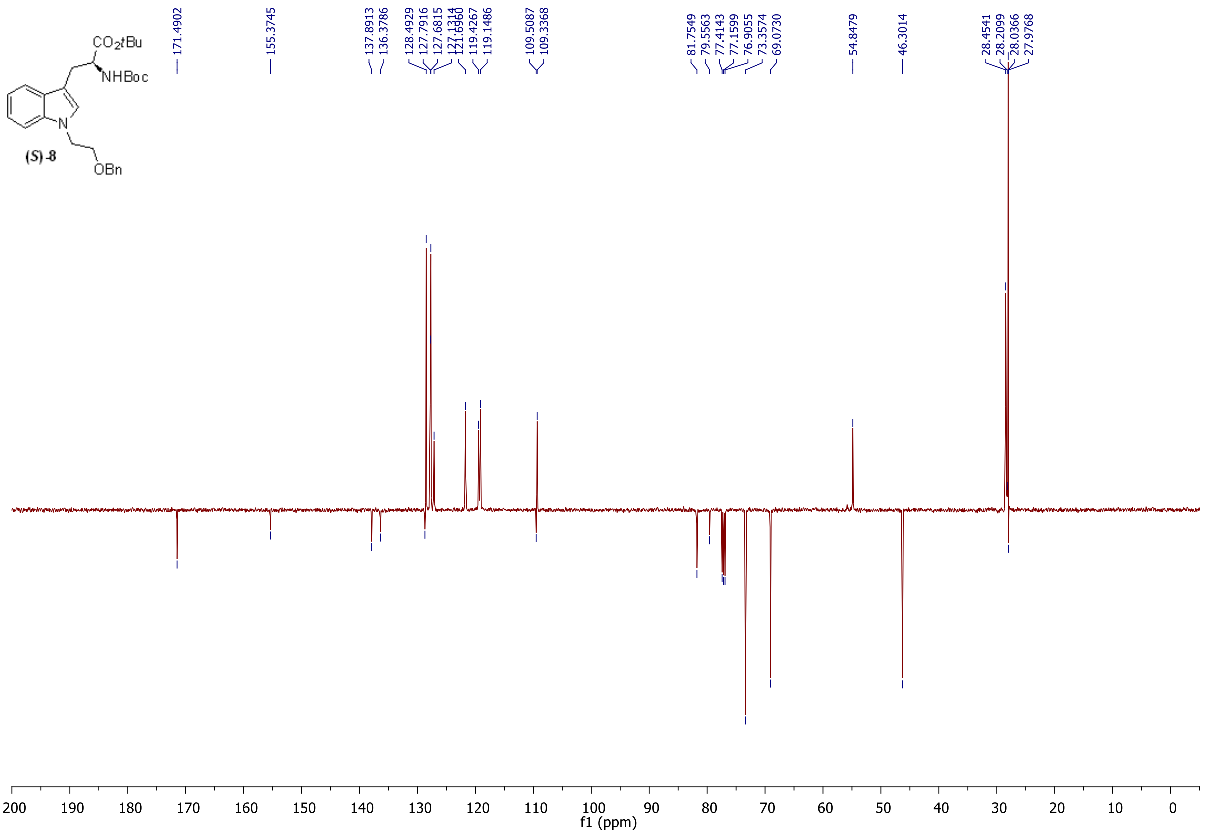

^{1}H (top) and ^{13}C (down) NMR spectra of compound **(*R*)-8** in $CDCl_3$

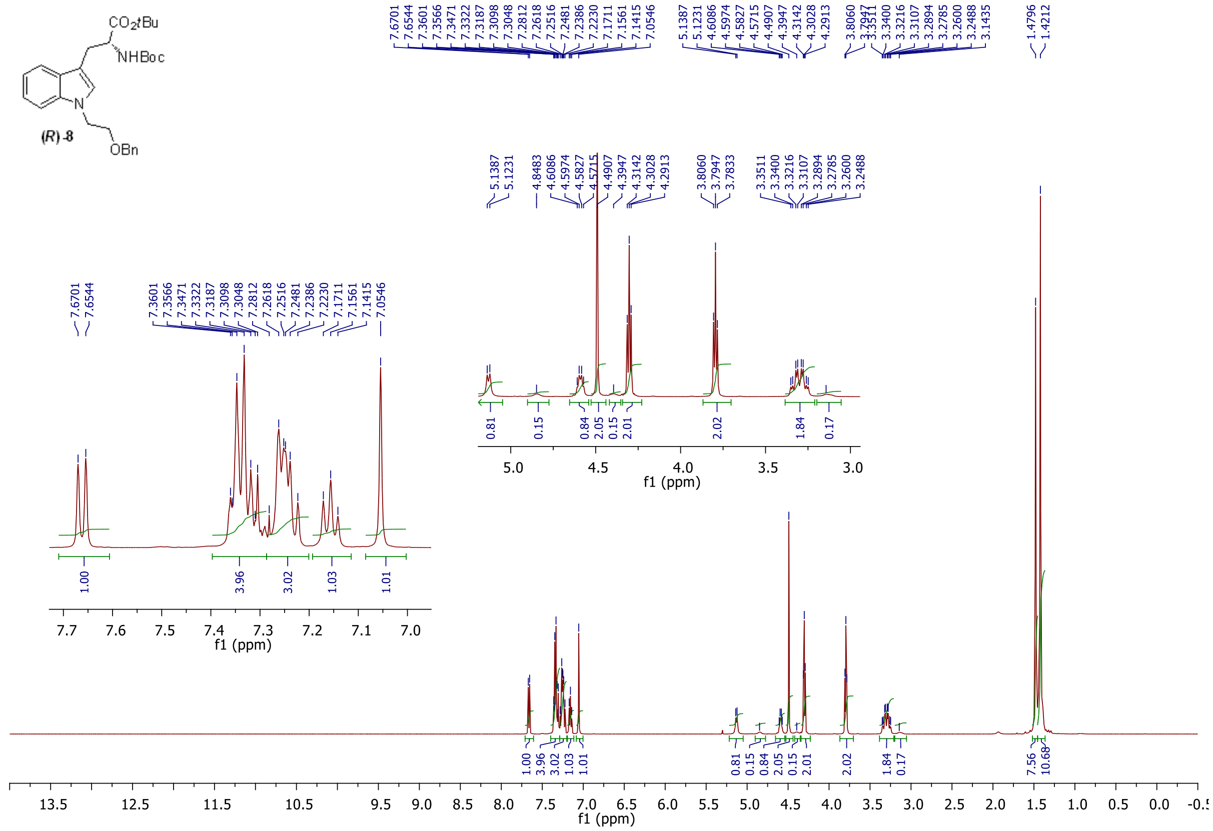

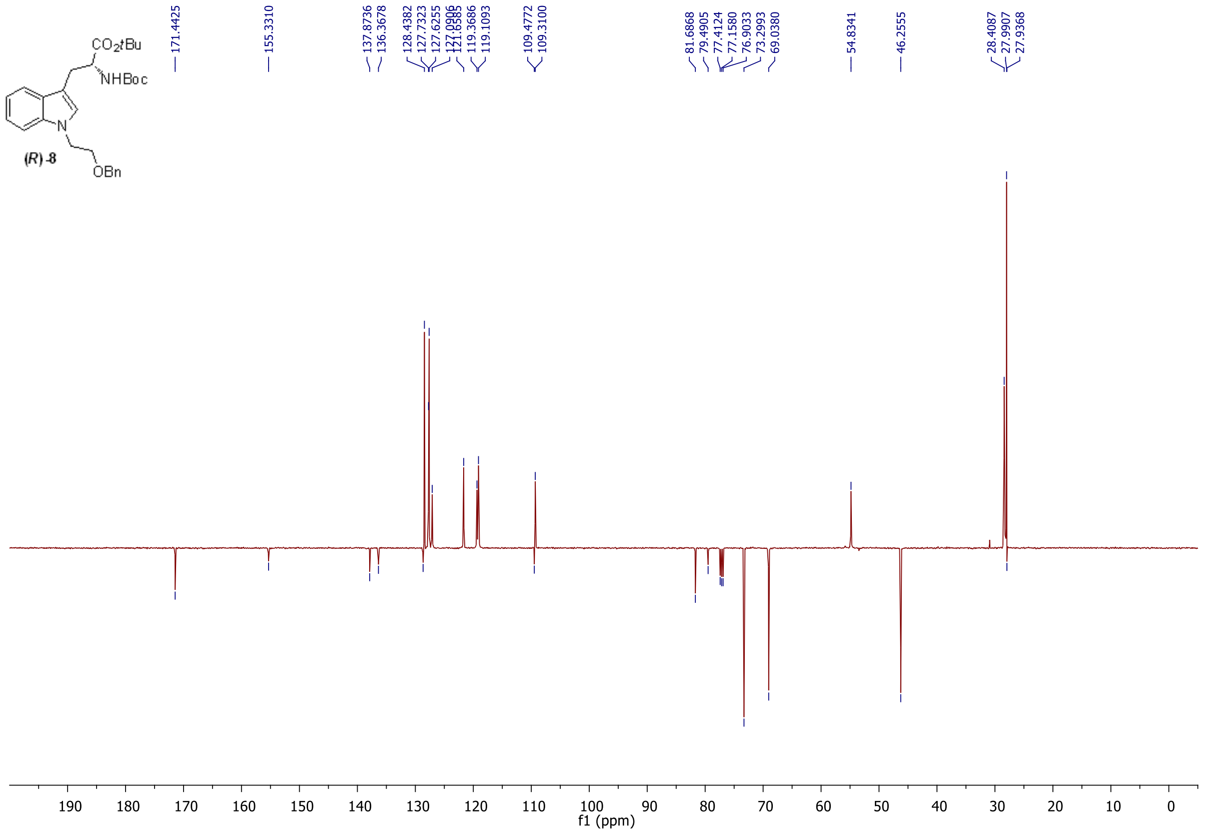

^{1}H (top) and ^{13}C (down) NMR spectra of compound **(*S*)-9** in CDCl$_3$

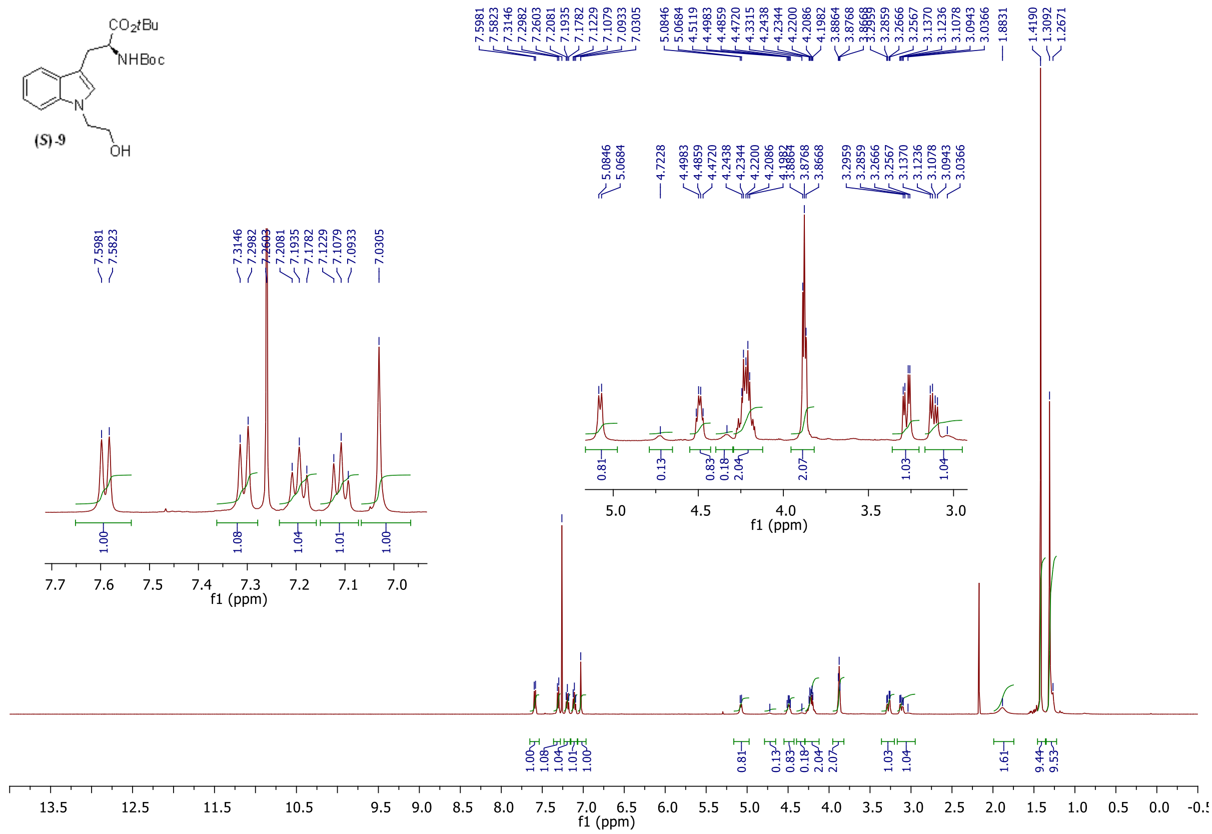

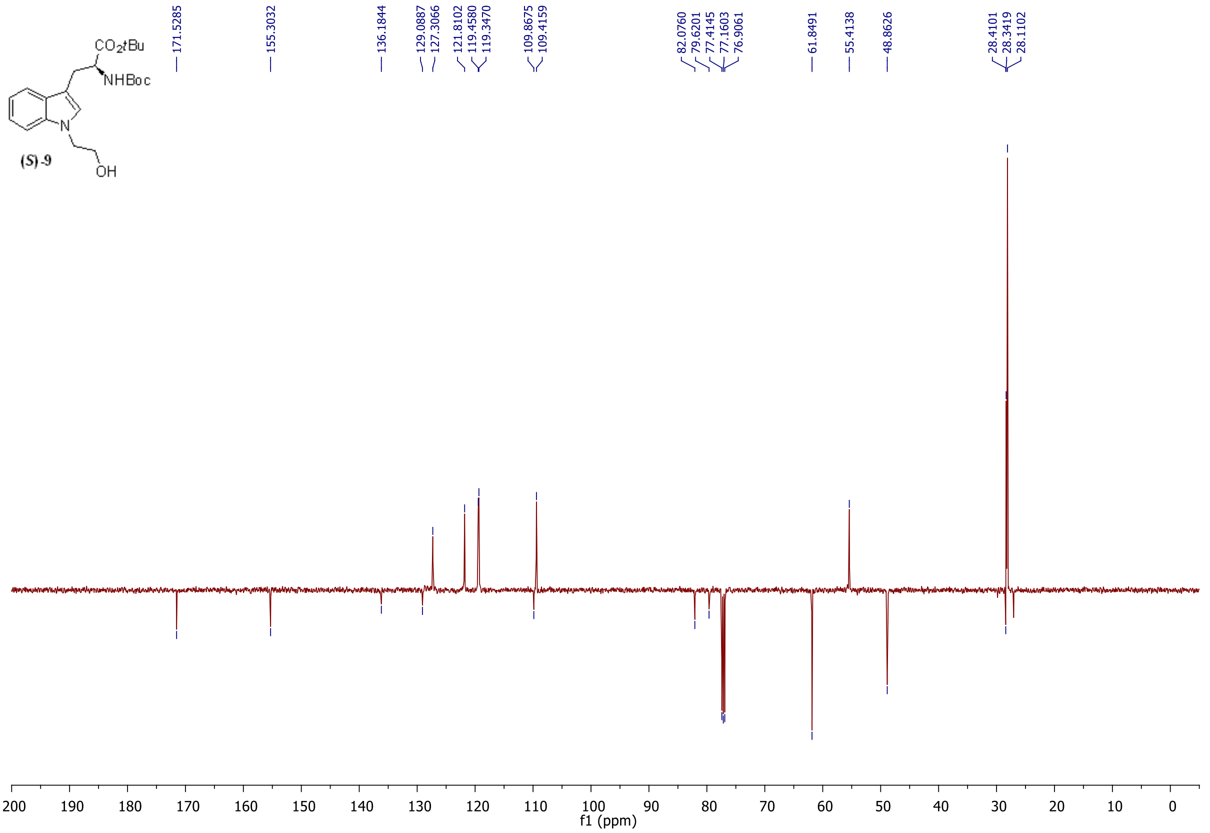

^{1}H (top) and ^{13}C (down) NMR spectra of compound **(*R*)-9** in $CDCl_3$

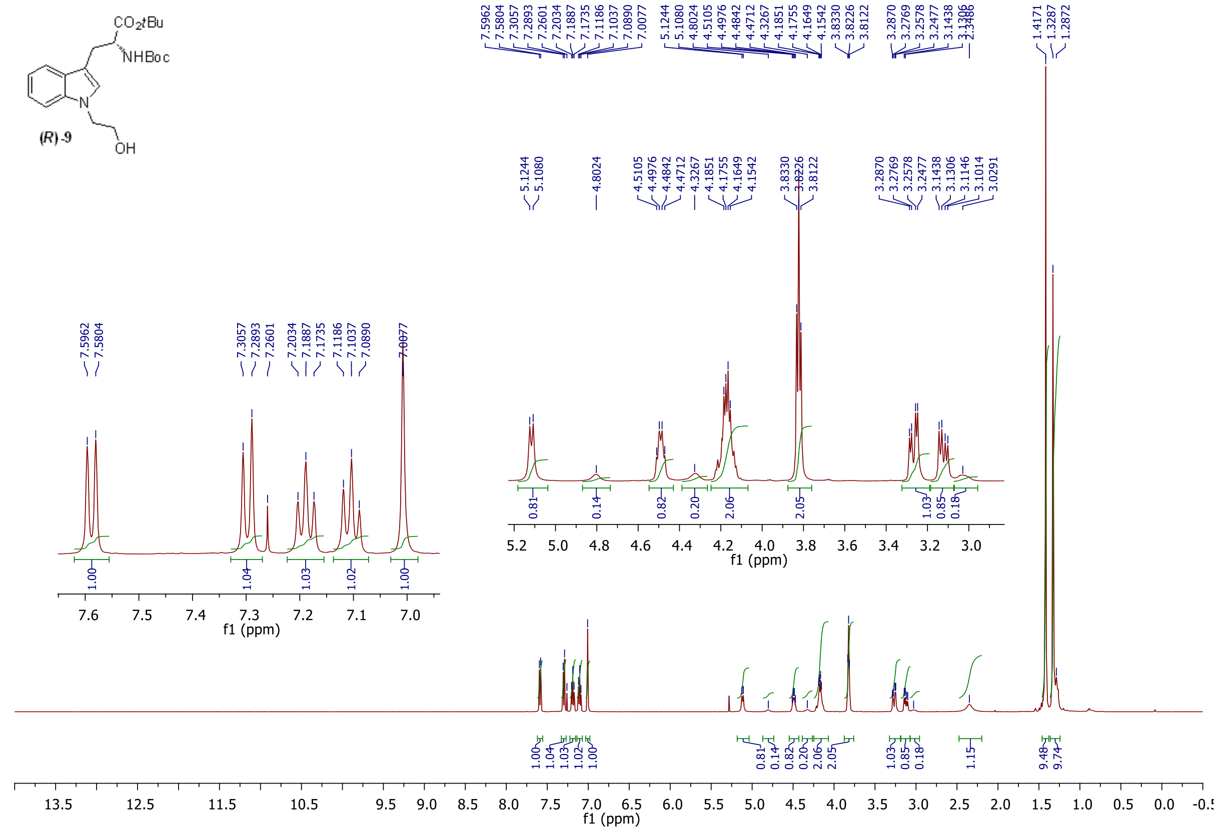

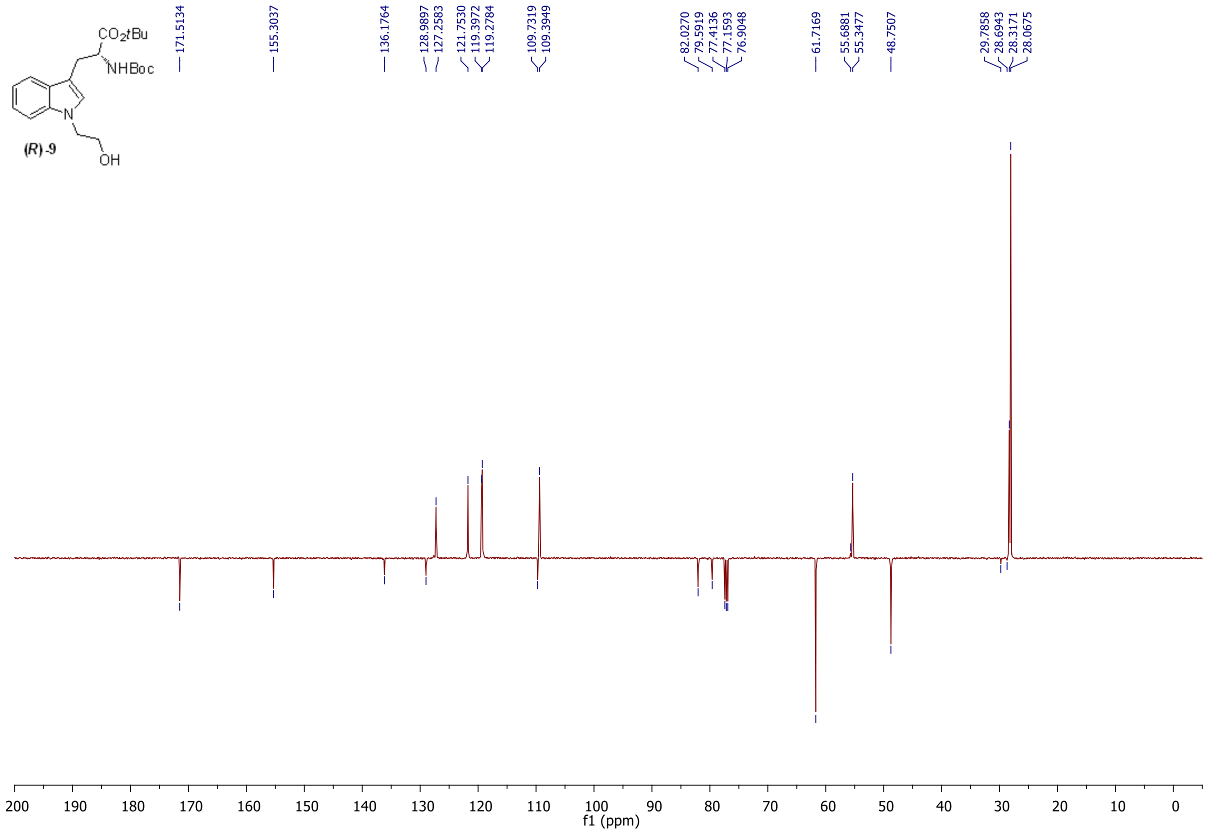

^{1}H (top) and ^{13}C (down) NMR spectra of compound **(*S*)-3** in $CDCl_3$

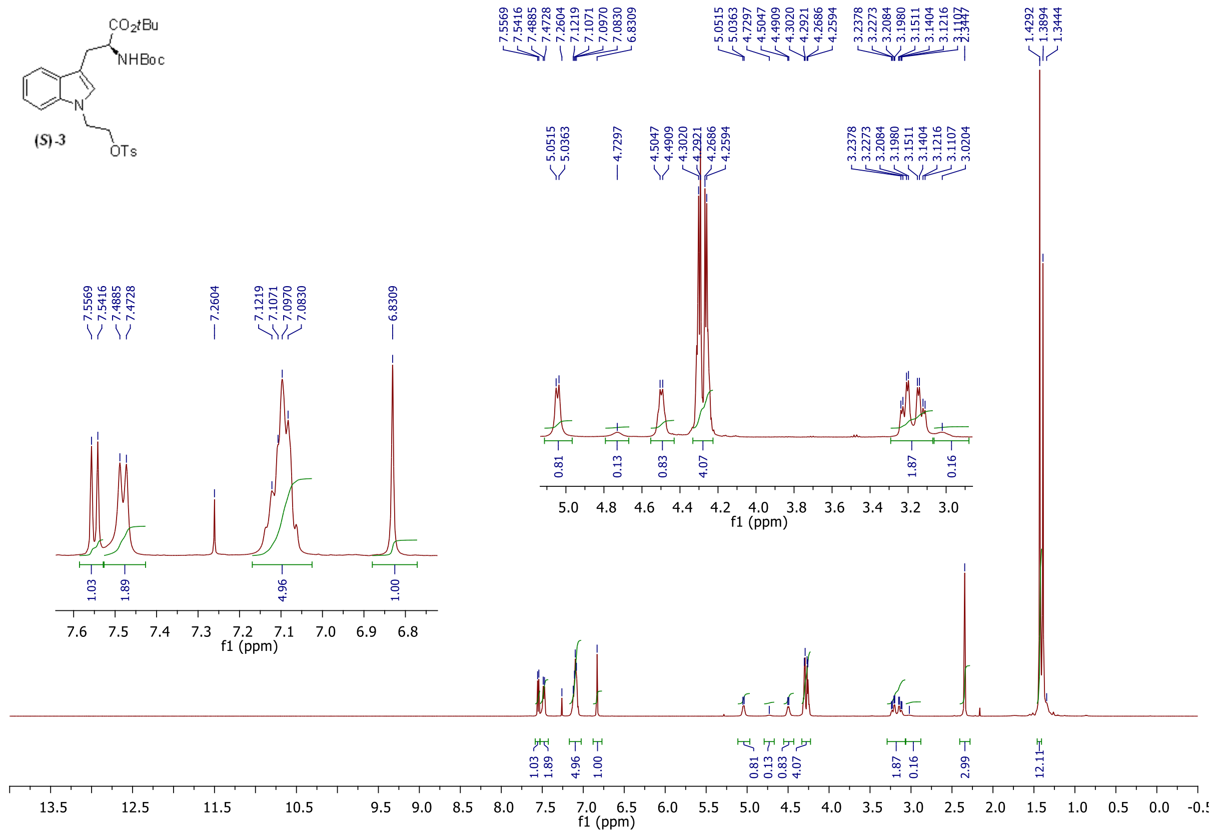

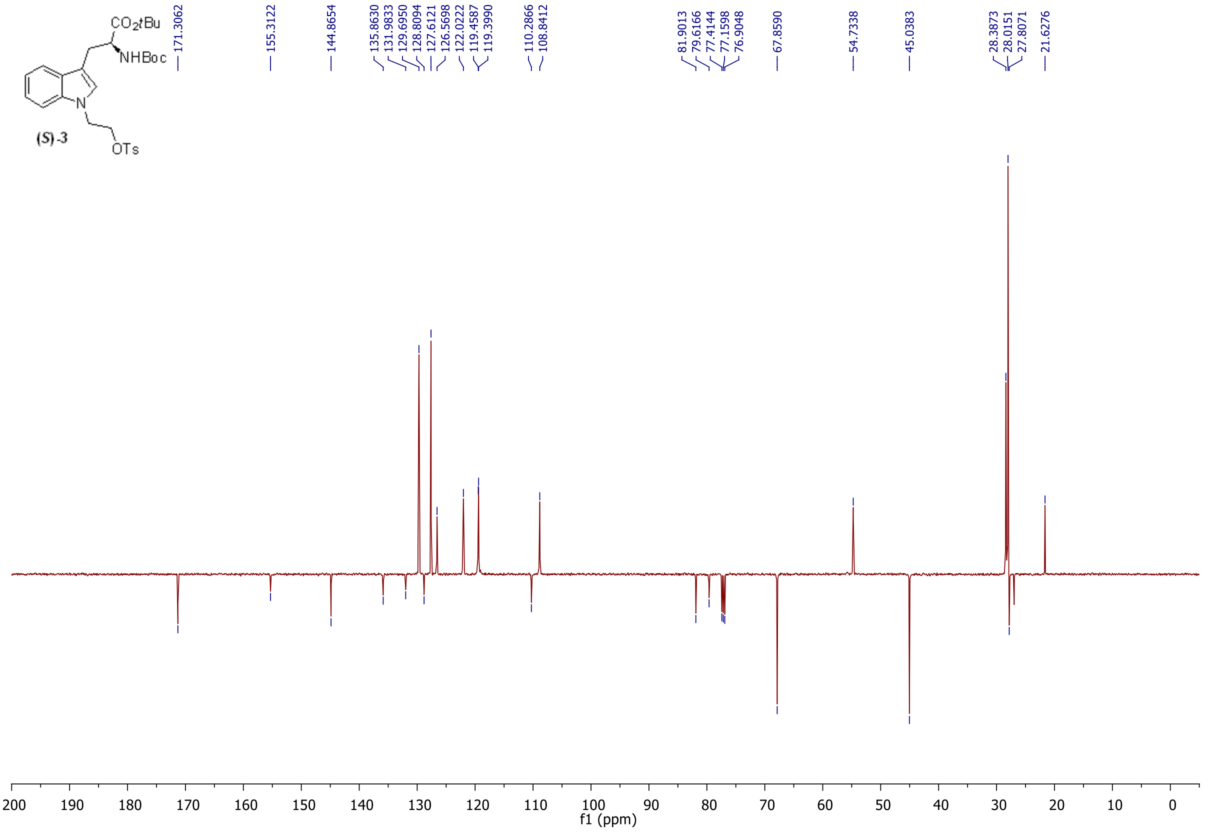

^{1}H (top) and ^{13}C (down) NMR spectra of compound **(*R*)-3** in $CDCl_3$

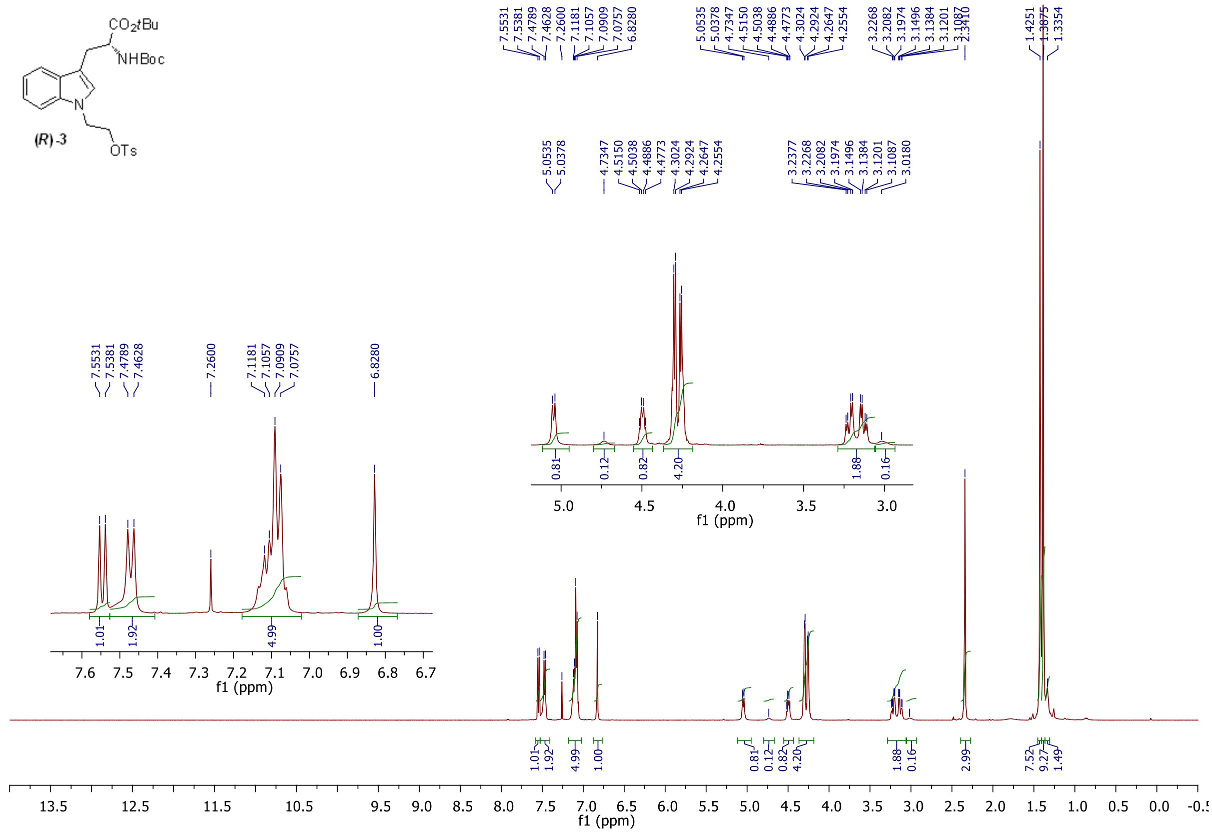

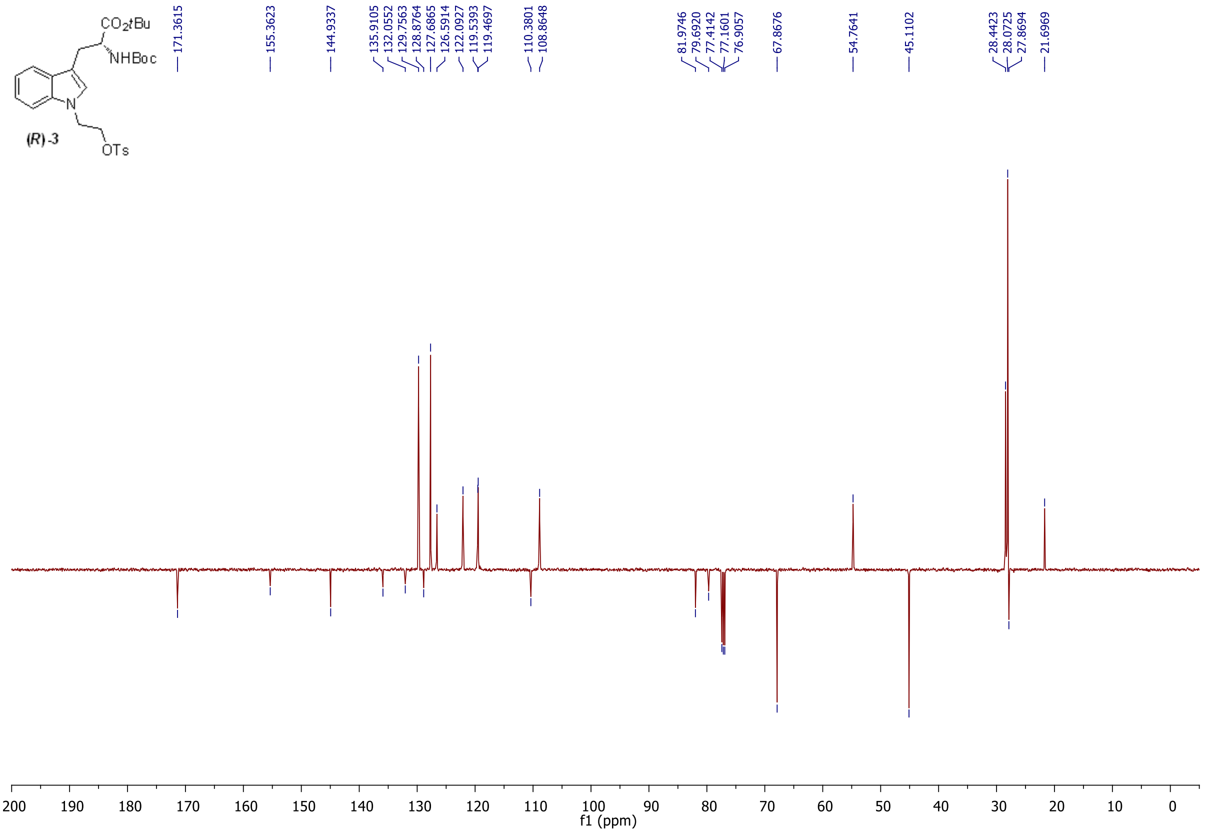

4. References

Henrottin J, Zervosen A, Lemaire C, Sapunaric F, Laurent S, Van den Eynde B et al. *N*[1]-Fluoroalkyltryptophan analogues: synthesis and in vitro study as potential substrates for indoleamine 2,3-dioxygenase. ACS Med Chem Lett. 2015;6(3):260-5.

Henrottin J, Lemaire C, Egrise D, Zervosen A, Van den Eynde B, Plenevaux A et al. Fully automated radiosynthesis of *N*[1]-[^{18}F]fluoroethyl-tryptophan and study of its biological activity as a new potential substrate for indoleamine 2,3-dioxygenase PET imaging. Nucl Med Biol. 2016;43(6):379-89.

Wadsworth HJ, O'Shea D, Passmore J, Trigg W, Ewan A, Shan B. Tricyclic indole derivates as PBR ligands. 2010, Patent WO2010109007.

Xin Y, Cai H. Improved radiosynthesis and biological evaluations of L- and D-1-[^{18}F]fluoroethyl-tryptophan for PET imaging of IDO-mediated kynurenine pathway of tryptophan metabolism. Mol Imaging Biol. 2017;19(4):589-98.
